# Supplementary material for: Characterizing and predicting person-specific, day-to-day, fluctuations in walking behavior
Source: PLoS One. 2021 May 14;16(5):e0251659. doi: 10.1371/journal.pone.0251659 (PMC8121346; doi:10.1371/journal.pone.0251659)
Supplement: S1 Fig — (PDF) [file pone.0251659.s001.pdf]

Participants plots for gains and losses over time

Dario Baretta & Guillaume Chevance

8/7/2020

```
## [[1]]
```

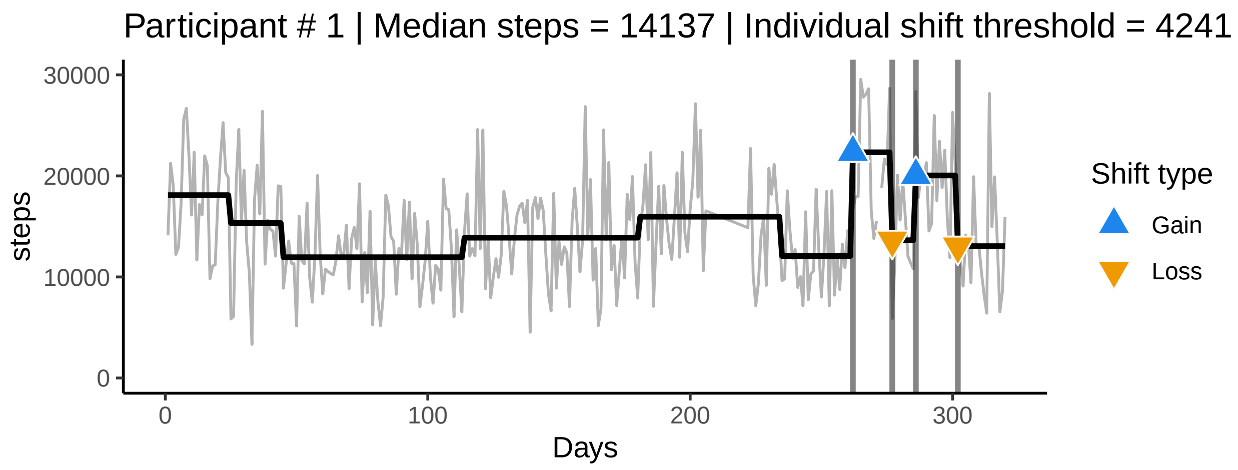

Participant # 1 | Median steps = 14137 | Individual shift threshold = 4241
steps
30000
20000
10000
0
0
100
200
300
Days
Shift type
Gain
Loss


```
##
## [[2]]
```

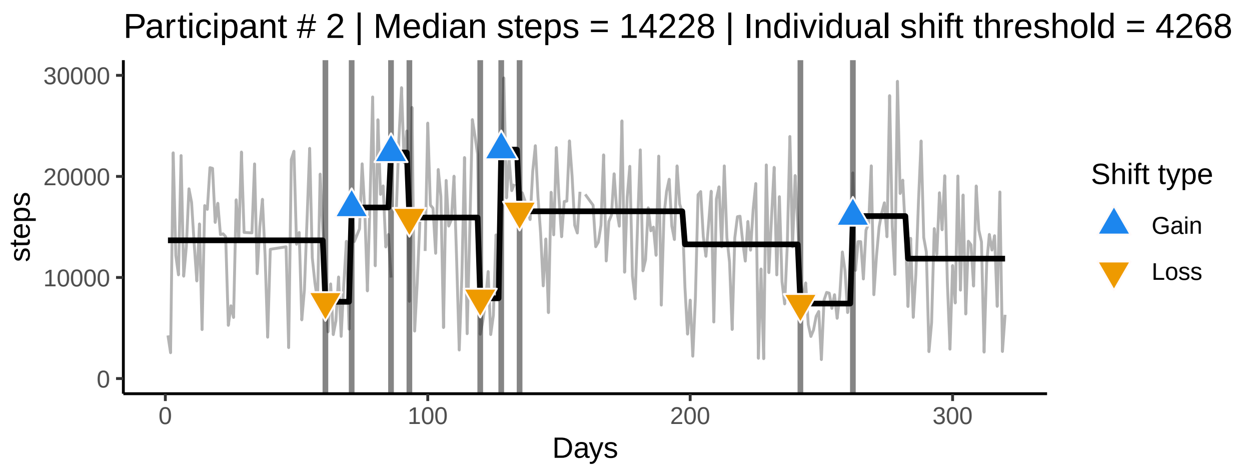

Participant # 2 | Median steps = 14228 | Individual shift threshold = 4268
steps
30000
20000
10000
0
0
100
200
300
Days
Shift type
Gain
Loss


```
##
## [[3]]
```

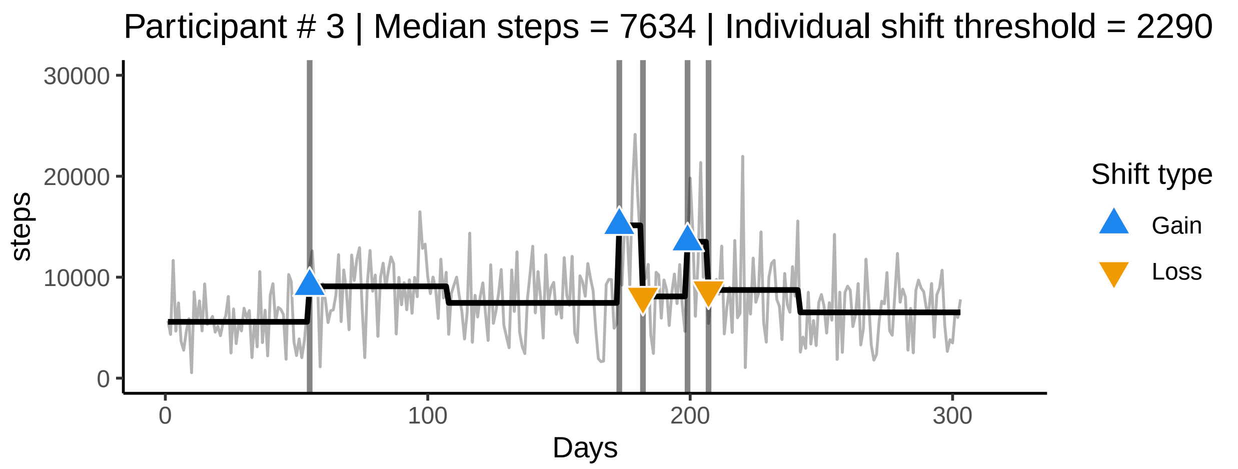

Participant # 3 | Median steps = 7634 | Individual shift threshold = 2290
steps
30000
20000
10000
0
0
100
200
300
Days
Shift type
Gain
Loss

```
##
## [[4]]
```

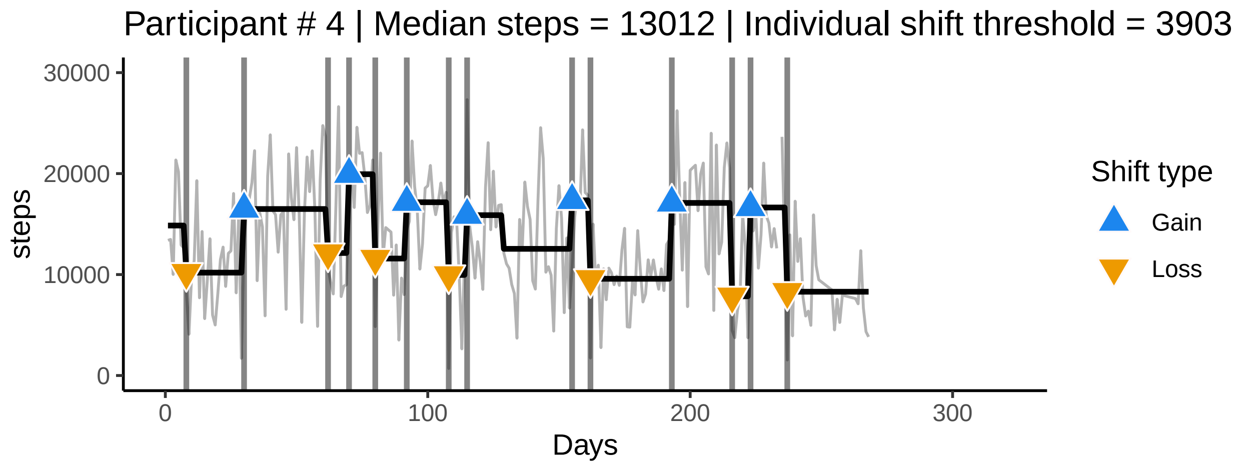

Participant # 4 | Median steps = 13012 | Individual shift threshold = 3903
30000
20000
10000
0
steps
0
100
200
300
Days
Shift type
Gain
Loss


```
##
## [[5]]
```

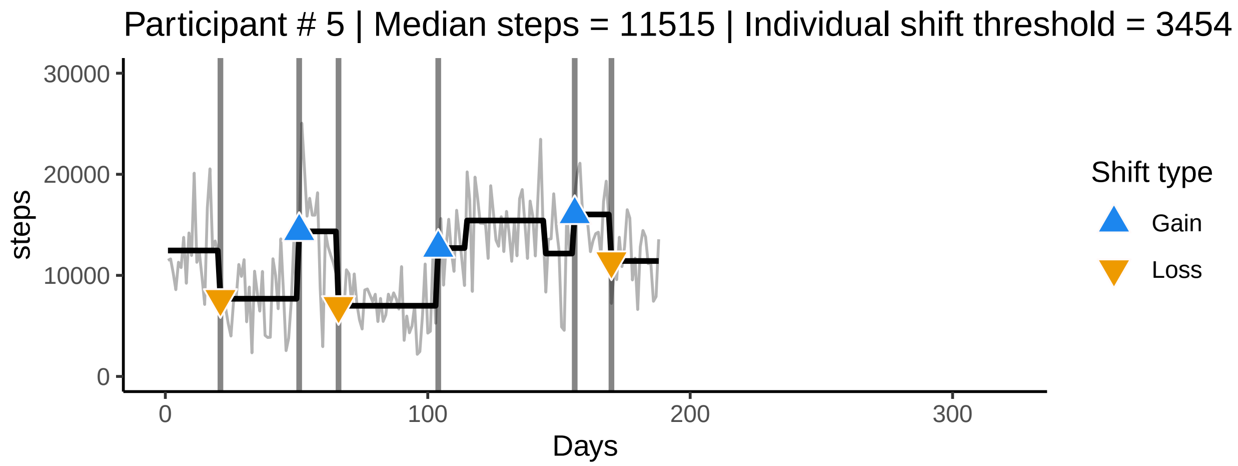

Participant # 5 | Median steps = 11515 | Individual shift threshold = 3454
30000
20000
10000
0
steps
0
100
200
300
Days
Shift type
Gain
Loss


```
##
## [[6]]
```

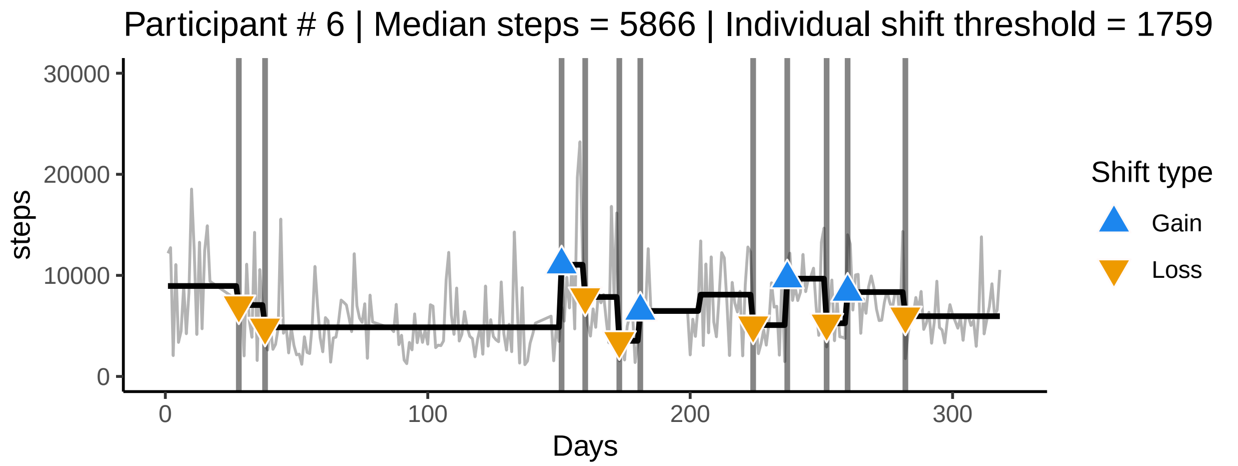

Participant # 6 | Median steps = 5866 | Individual shift threshold = 1759
30000
20000
10000
0
steps
0
100
200
300
Days
Shift type
Gain
Loss

```
##
## [[7]]
```

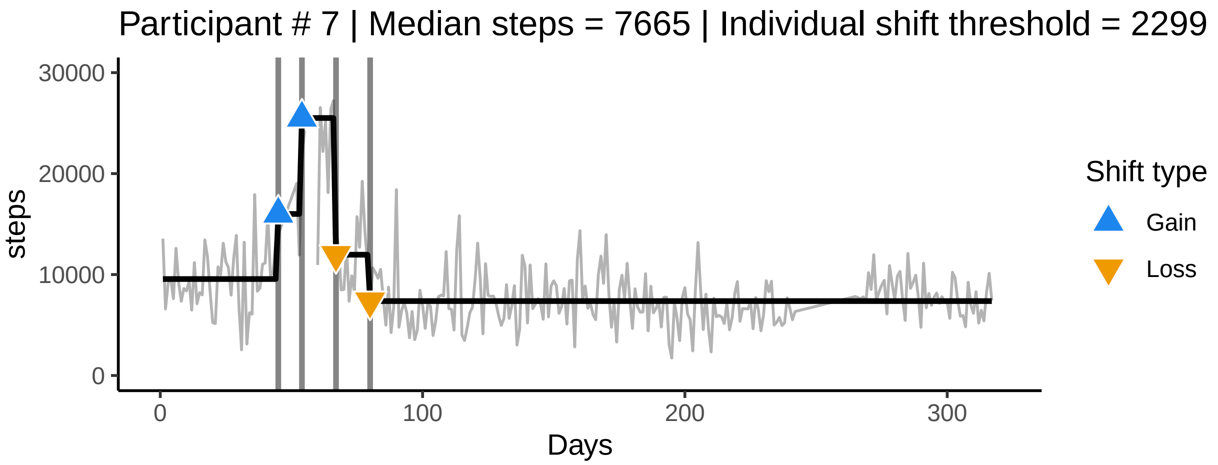

Participant # 7 | Median steps = 7665 | Individual shift threshold = 2299
steps
30000
20000
10000
0
0
100
200
300
Days
Shift type
Gain
Loss


```
##
## [[8]]
```

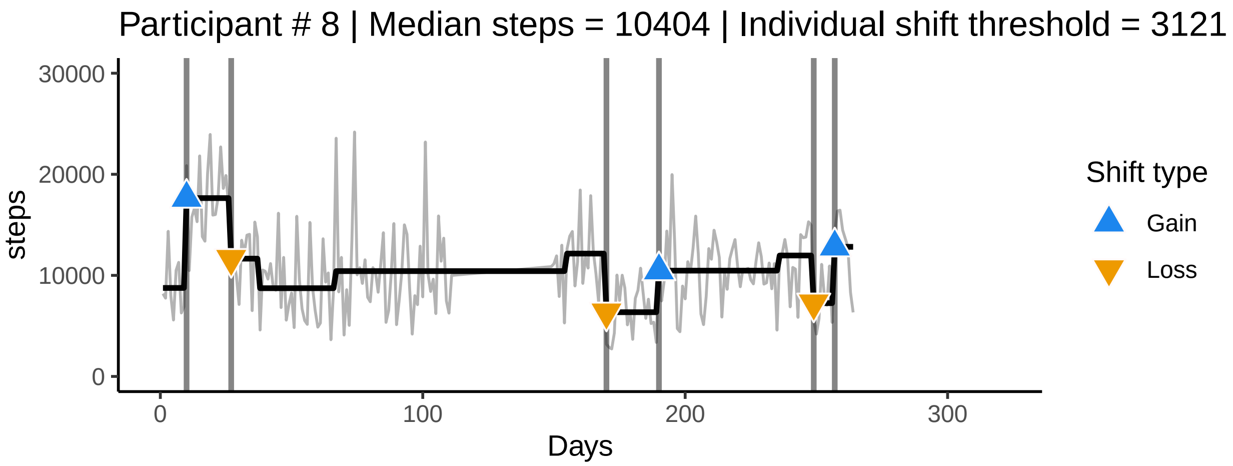

Participant # 8 | Median steps = 10404 | Individual shift threshold = 3121
steps
30000
20000
10000
0
0
100
200
300
Days
Shift type
Gain
Loss


```
##
## [[9]]
```

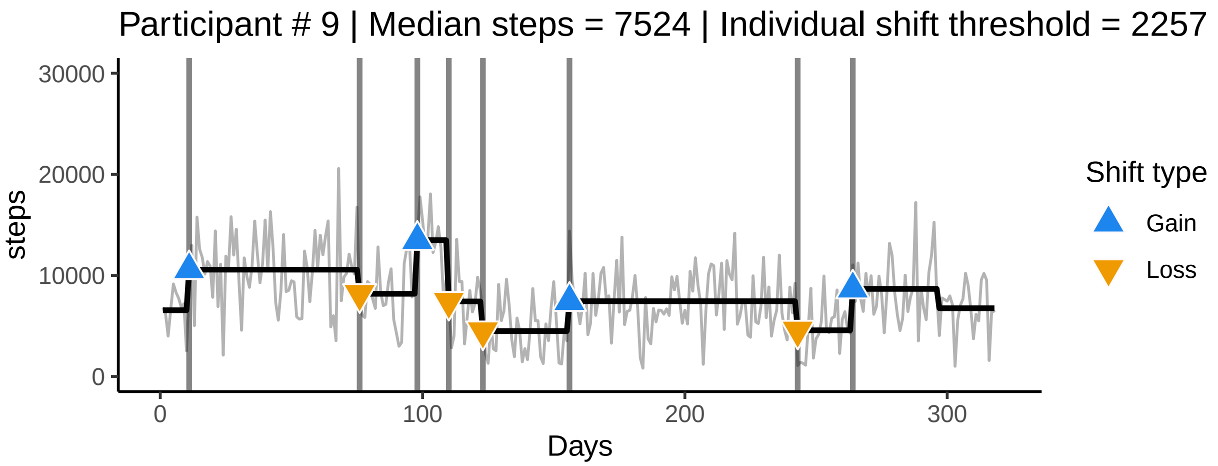

Participant # 9 | Median steps = 7524 | Individual shift threshold = 2257
steps
30000
20000
10000
0
0
100
200
300
Days
Shift type
Gain
Loss

```
##
## [[10]]
```

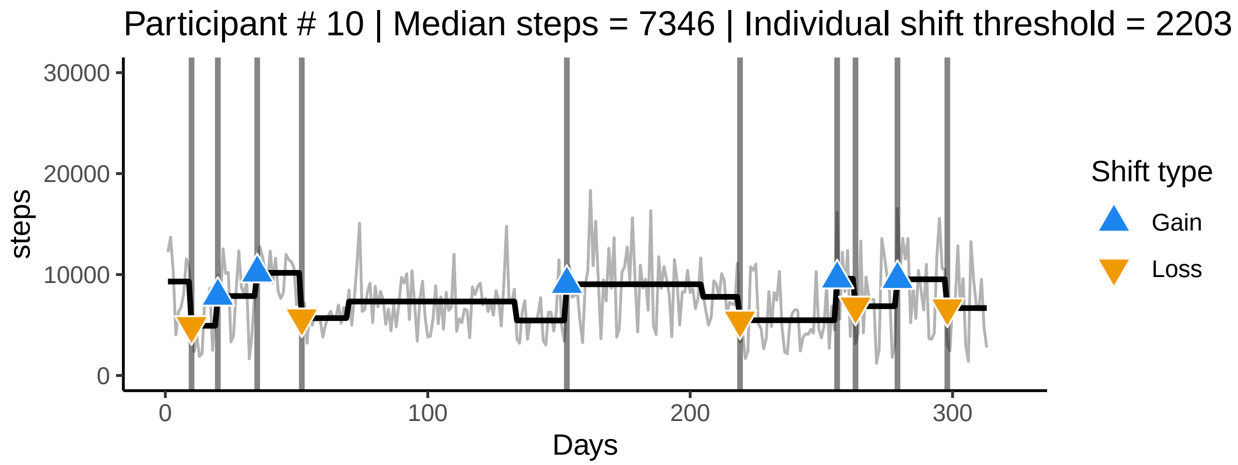

Participant # 10 | Median steps = 7346 | Individual shift threshold = 2203
steps
Days
Shift type
Gain
Loss


```
##
## [[11]]
```

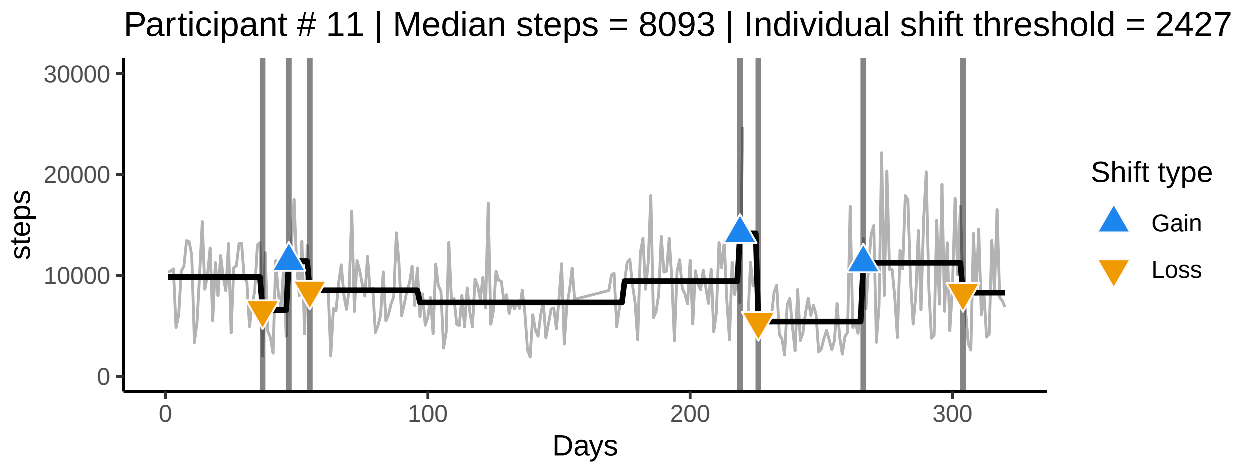

Participant # 11 | Median steps = 8093 | Individual shift threshold = 2427
steps
Days
Shift type
Gain
Loss


```
##
## [[12]]
```

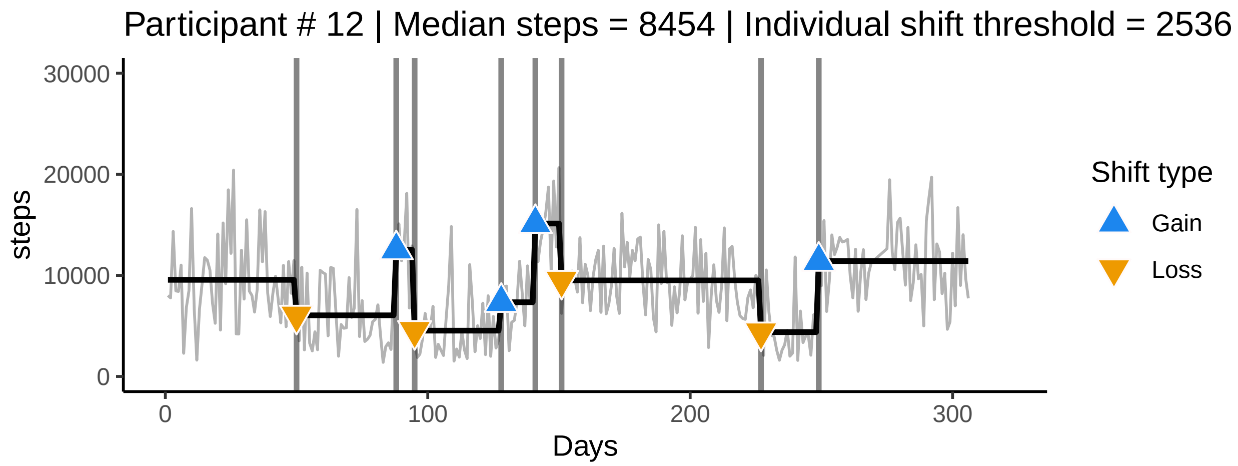

Participant # 12 | Median steps = 8454 | Individual shift threshold = 2536
steps
Days
Shift type
Gain
Loss

```
##
## [[13]]
```

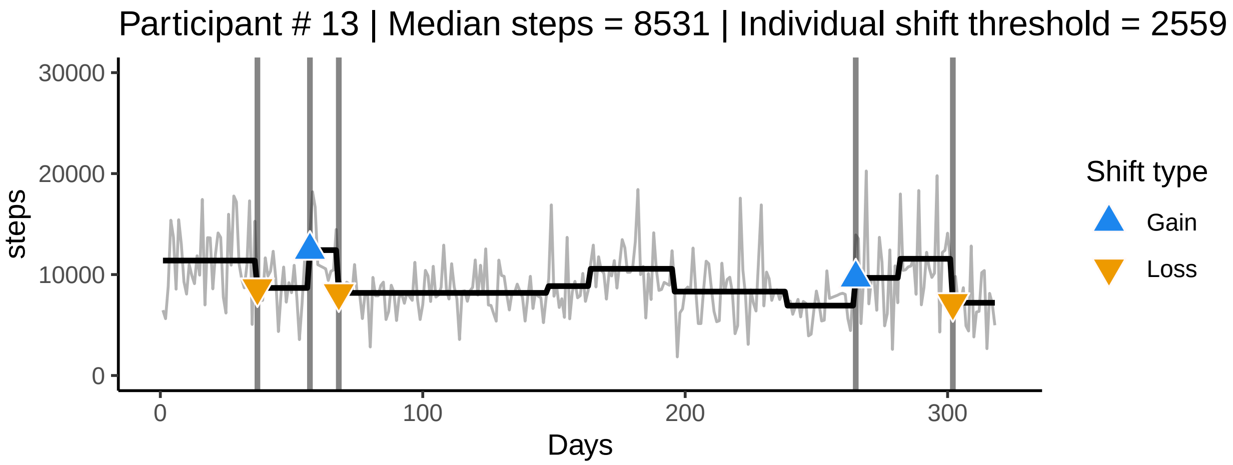

Participant # 13 | Median steps = 8531 | Individual shift threshold = 2559
30000
20000
10000
0
steps
0
100
200
300
Days
Shift type
Gain
Loss


```
##
## [[14]]
```

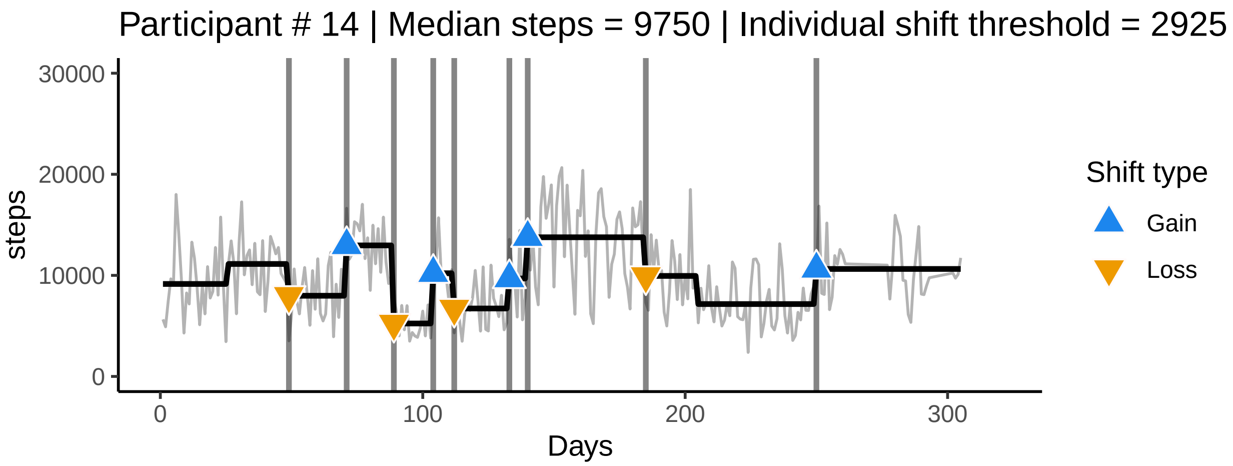

Participant # 14 | Median steps = 9750 | Individual shift threshold = 2925
30000
20000
10000
0
steps
0
100
200
300
Days
Shift type
Gain
Loss


```
##
## [[15]]
```

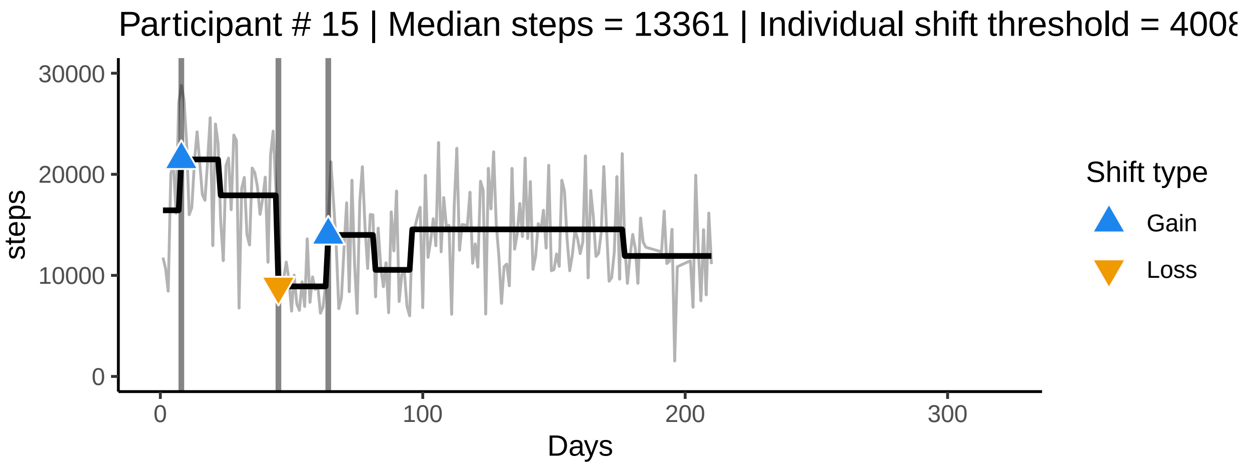

Participant # 15 | Median steps = 13361 | Individual shift threshold = 400
30000
20000
10000
0
steps
0
100
200
300
Days
Shift type
Gain
Loss

```
##
## [[16]]
```

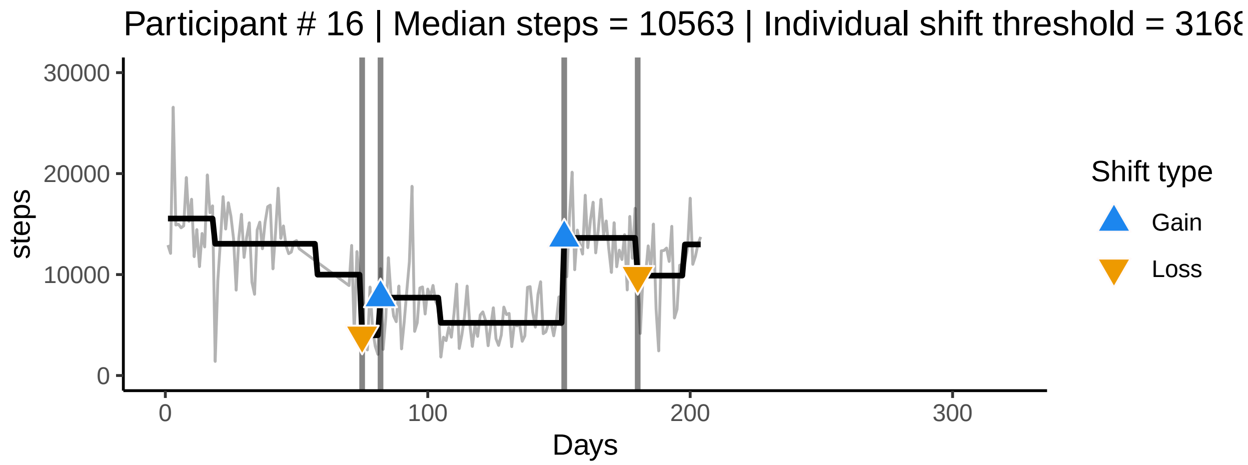

Participant # 16 | Median steps = 10563 | Individual shift threshold = 316
steps
30000
20000
10000
0
0
100
200
300
Days
Shift type
Gain
Loss


```
##
## [[17]]
```

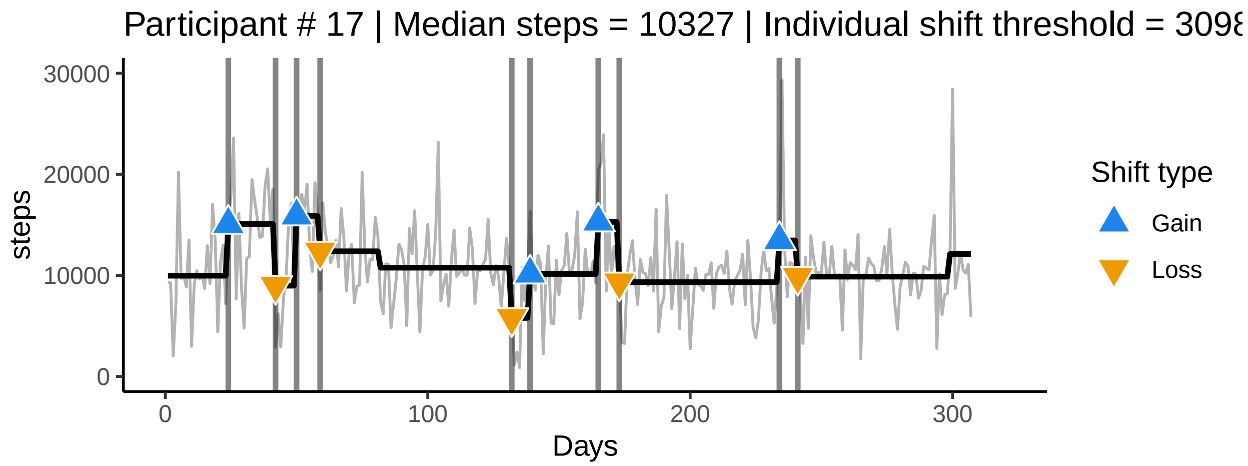

Participant # 17 | Median steps = 10327 | Individual shift threshold = 309
steps
30000
20000
10000
0
0
100
200
300
Days
Shift type
Gain
Loss


```
##
## [[18]]
```

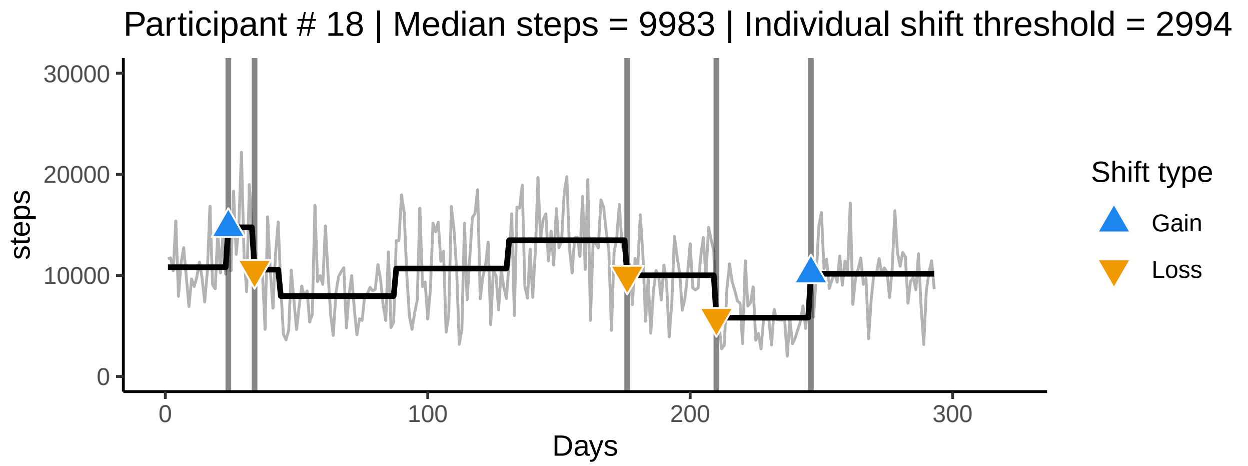

Participant # 18 | Median steps = 9983 | Individual shift threshold = 2994
steps
30000
20000
10000
0
0
100
200
300
Days
Shift type
Gain
Loss

```
##
## [[19]]
```

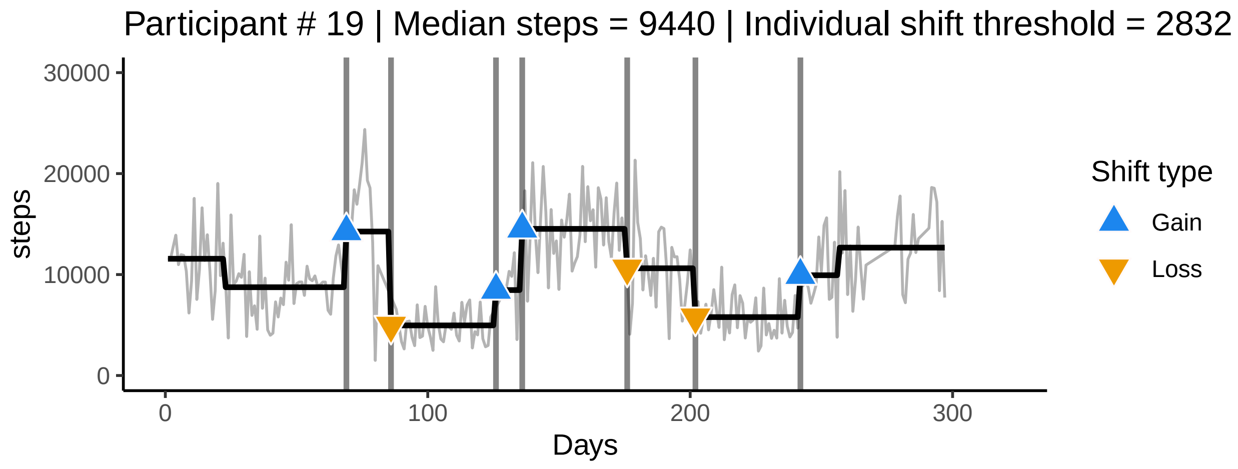
Participant # 19 | Median steps = 9440 | Individual shift threshold = 2832
steps
30000
20000
10000
0
0
100
200
300
Days
Shift type
Gain
Loss

```
##
## [[20]]
```

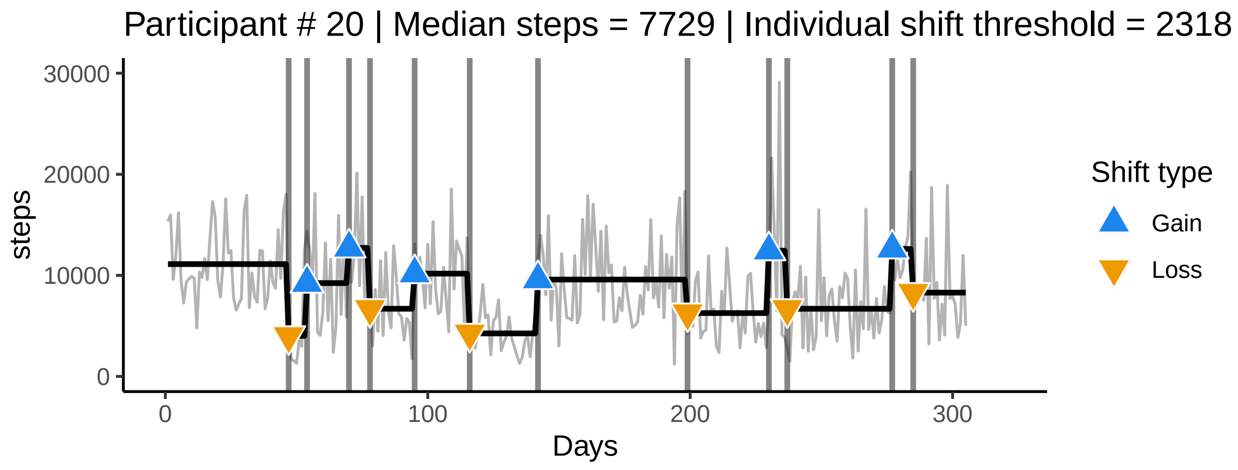
Participant # 20 | Median steps = 7729 | Individual shift threshold = 2318
steps
30000
20000
10000
0
0
100
200
300
Days
Shift type
Gain
Loss

```
##
## [[21]]
```

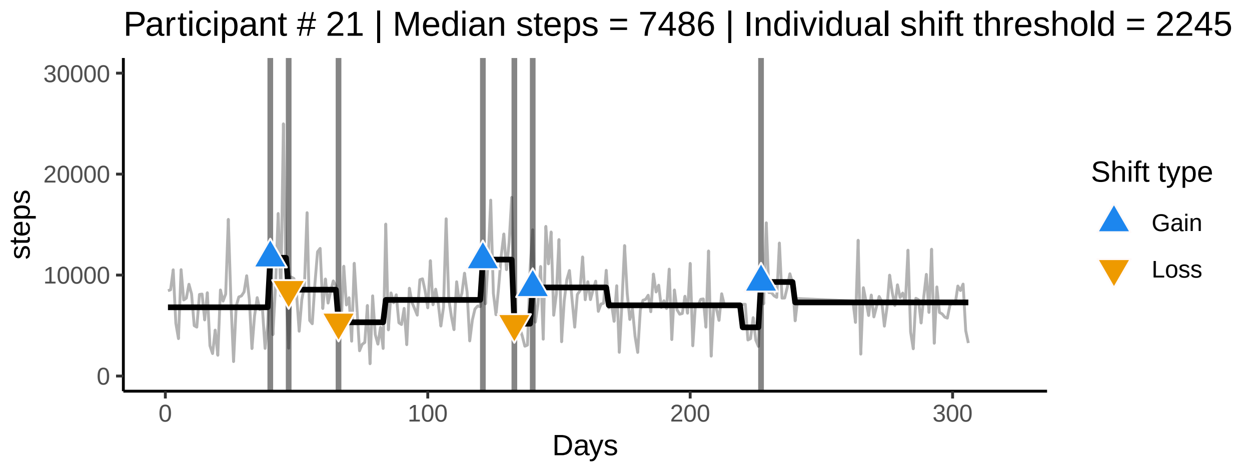
Participant # 21 | Median steps = 7486 | Individual shift threshold = 2245
steps
30000
20000
10000
0
0
100
200
300
Days
Shift type
Gain
Loss

```
##
## [[22]]
```

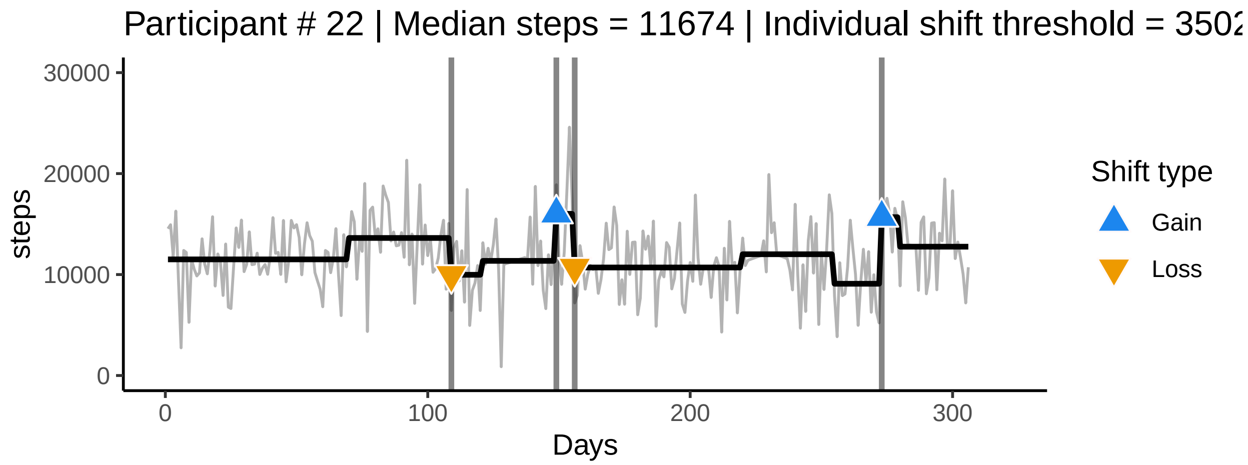

Participant # 22 | Median steps = 11674 | Individual shift threshold = 3502
30000
20000
10000
0
steps
0
100
200
300
Days
Shift type
Gain
Loss


```
##
## [[23]]
```

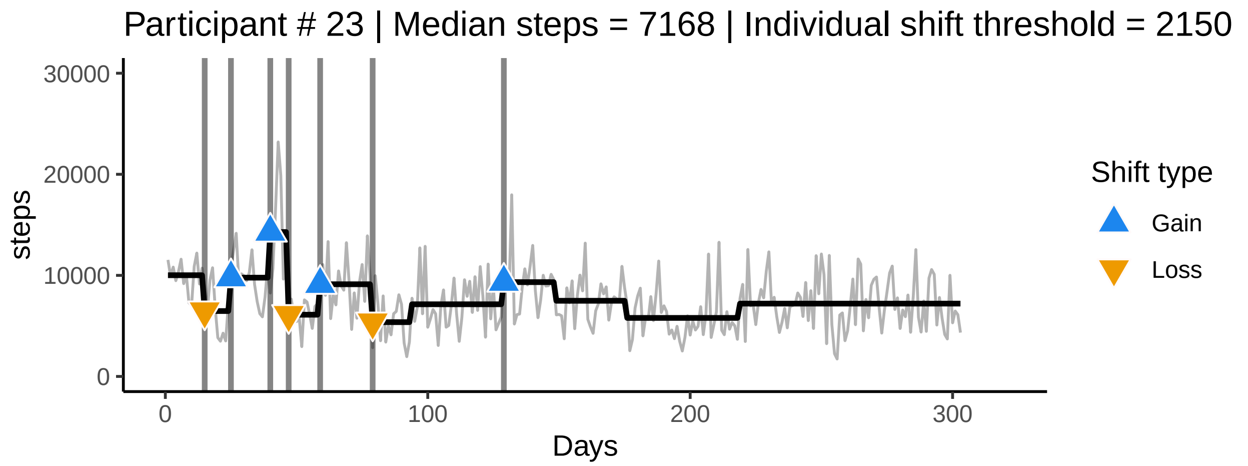

Participant # 23 | Median steps = 7168 | Individual shift threshold = 2150
30000
20000
10000
0
steps
0
100
200
300
Days
Shift type
Gain
Loss


```
##
## [[24]]
```

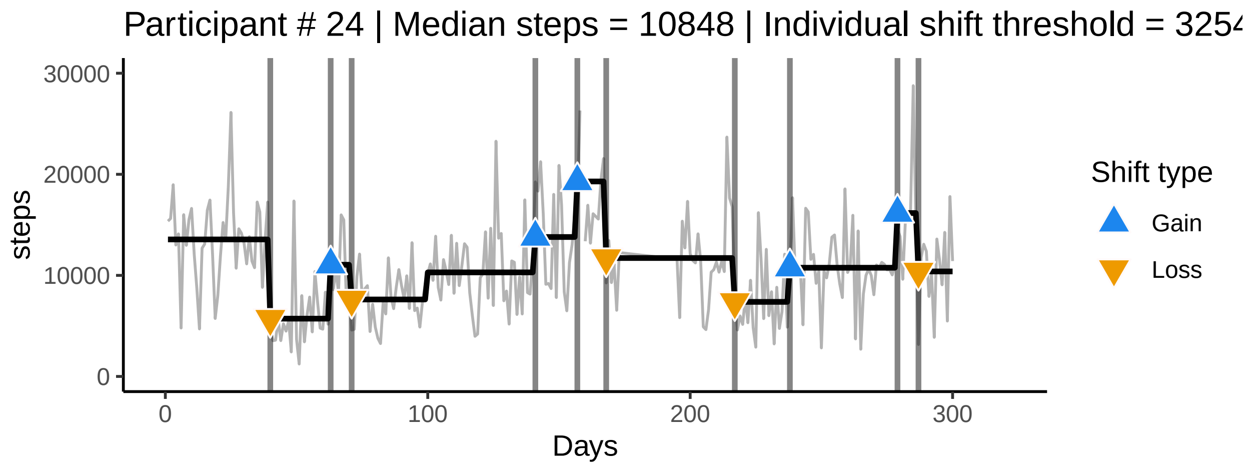

Participant # 24 | Median steps = 10848 | Individual shift threshold = 3254
30000
20000
10000
0
steps
0
100
200
300
Days
Shift type
Gain
Loss

```
##
## [[25]]
```

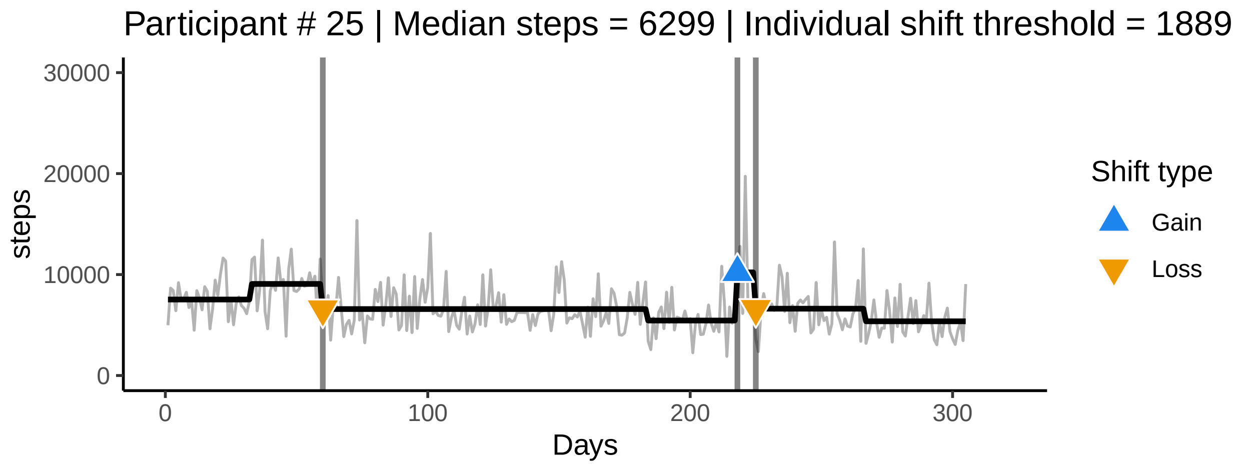
Participant # 25 | Median steps = 6299 | Individual shift threshold = 1889
30000
20000
10000
0
steps
0
100
200
300
Days
Shift type
Gain
Loss

```
##
## [[26]]
```

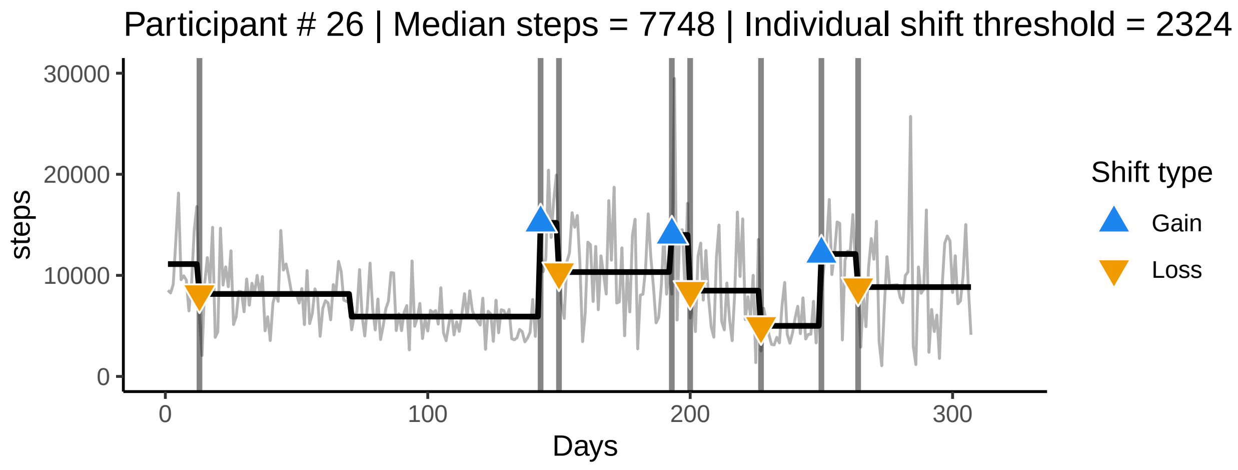
Participant # 26 | Median steps = 7748 | Individual shift threshold = 2324
30000
20000
10000
0
steps
0
100
200
300
Days
Shift type
Gain
Loss

```
##
## [[27]]
```

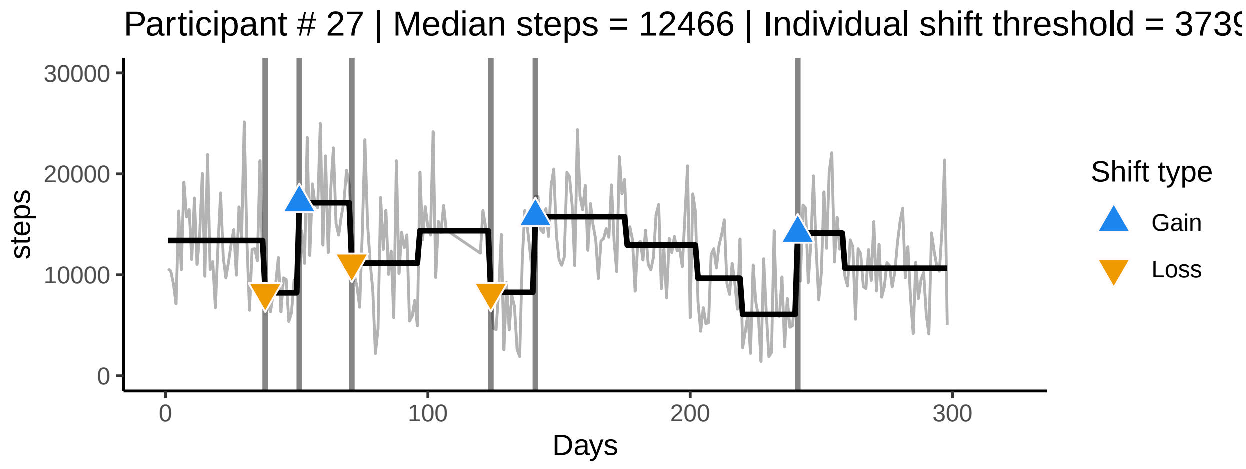
Participant # 27 | Median steps = 12466 | Individual shift threshold = 373
30000
20000
10000
0
steps
0
100
200
300
Days
Shift type
Gain
Loss

```
##
## [[28]]
```

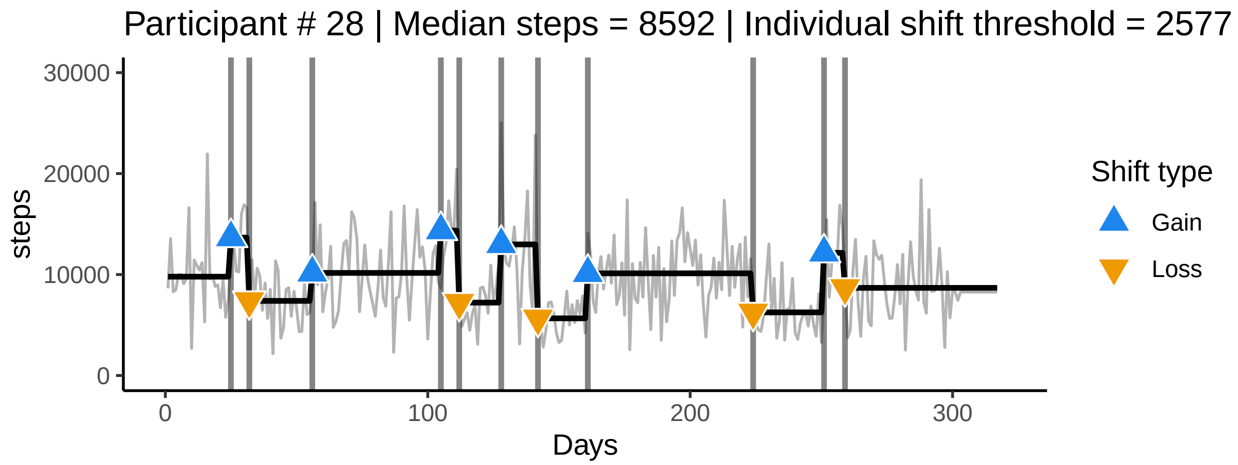

Participant # 28 | Median steps = 8592 | Individual shift threshold = 2577
steps
30000
20000
10000
0
0
100
200
300
Days
Shift type
Gain
Loss


```
##
## [[29]]
```

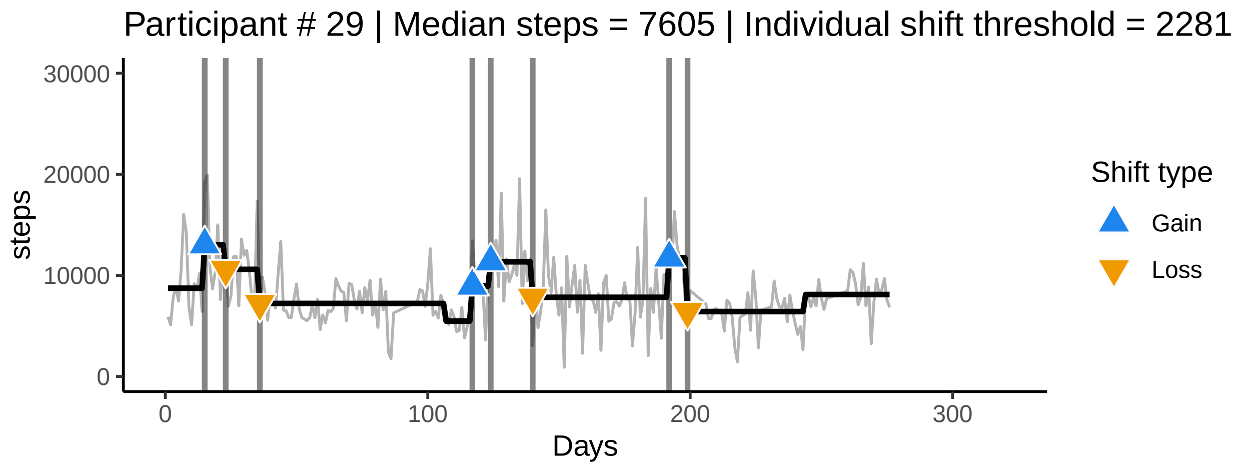

Participant # 29 | Median steps = 7605 | Individual shift threshold = 2281
steps
30000
20000
10000
0
0
100
200
300
Days
Shift type
Gain
Loss


```
##
## [[30]]
```

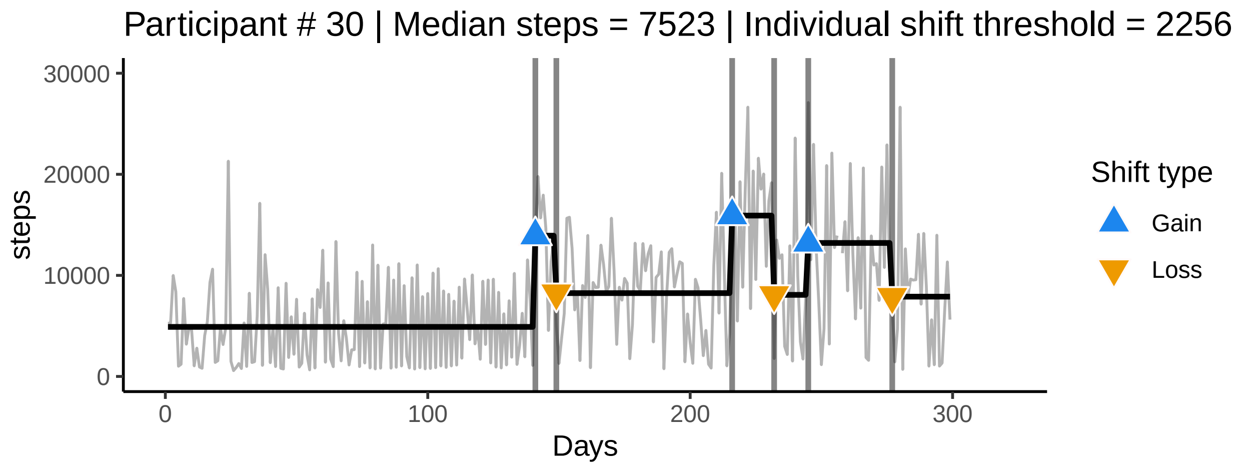

Participant # 30 | Median steps = 7523 | Individual shift threshold = 2256
steps
30000
20000
10000
0
0
100
200
300
Days
Shift type
Gain
Loss

```
##
## [[31]]
```

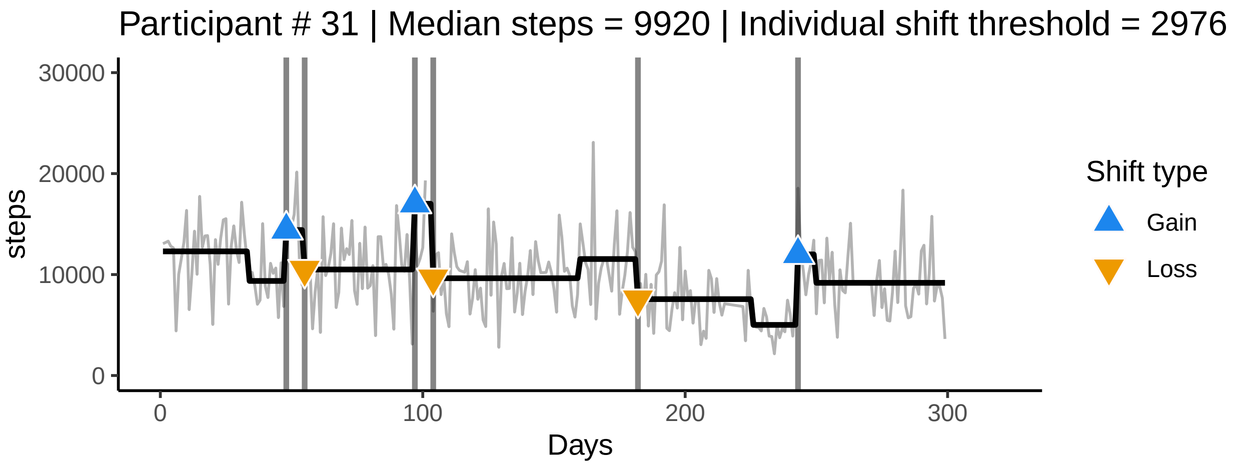
Participant # 31 | Median steps = 9920 | Individual shift threshold = 2976
steps
30000
20000
10000
0
0
100
200
300
Days
Shift type
Gain
Loss

```
##
## [[32]]
```

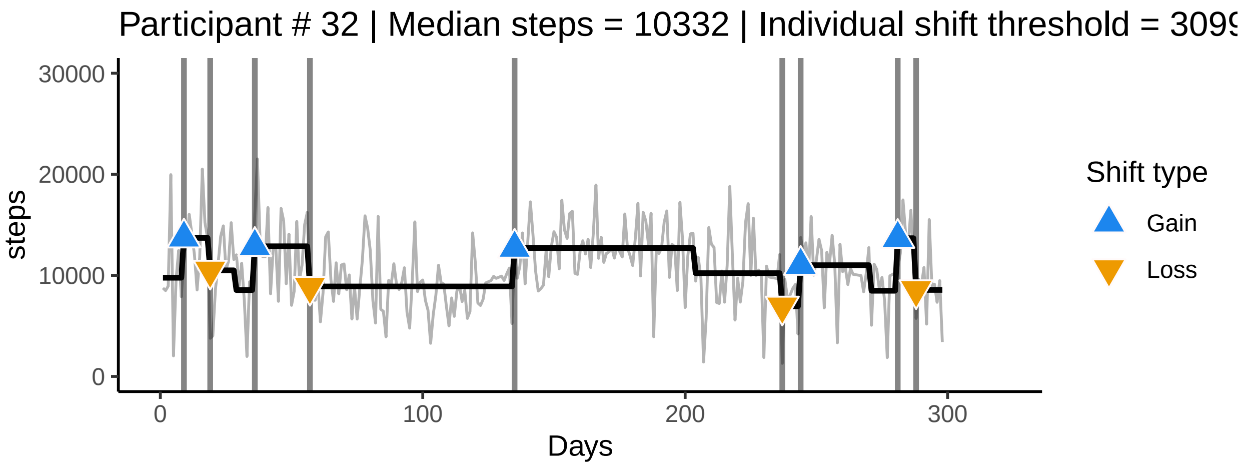
Participant # 32 | Median steps = 10332 | Individual shift threshold = 3099
steps
30000
20000
10000
0
0
100
200
300
Days
Shift type
Gain
Loss

```
##
## [[33]]
```

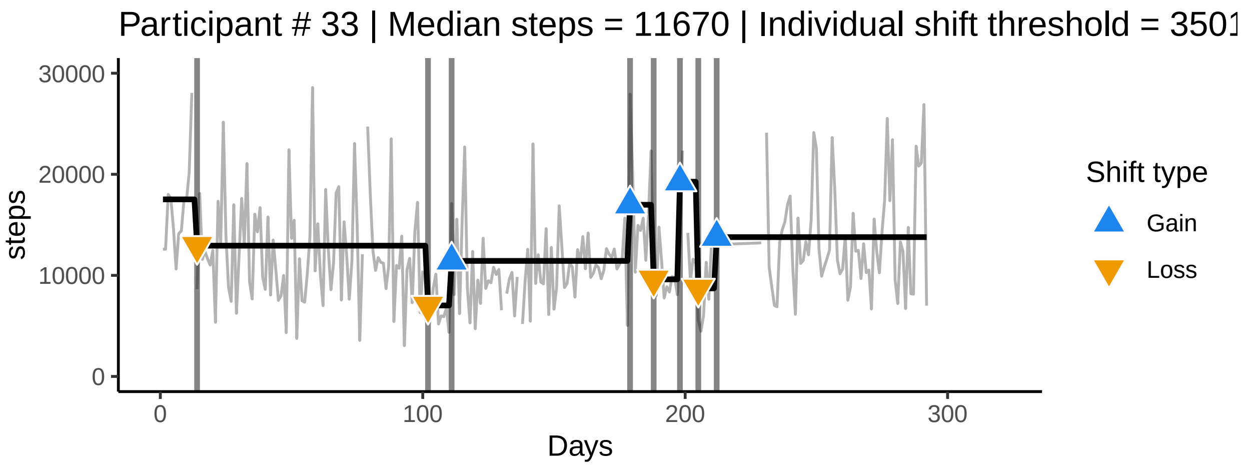
Participant # 33 | Median steps = 11670 | Individual shift threshold = 3501
steps
30000
20000
10000
0
0
100
200
300
Days
Shift type
Gain
Loss

```
##
## [[34]]
```

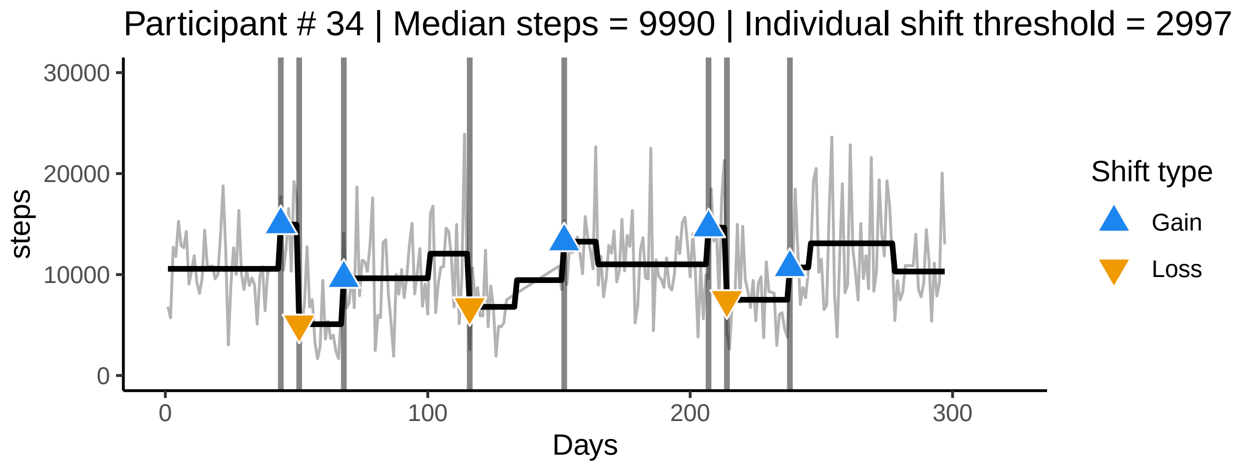
Participant # 34 | Median steps = 9990 | Individual shift threshold = 2997
30000
20000
10000
0
steps
0
100
200
300
Days
Shift type
Gain
Loss

```
##
## [[35]]
```

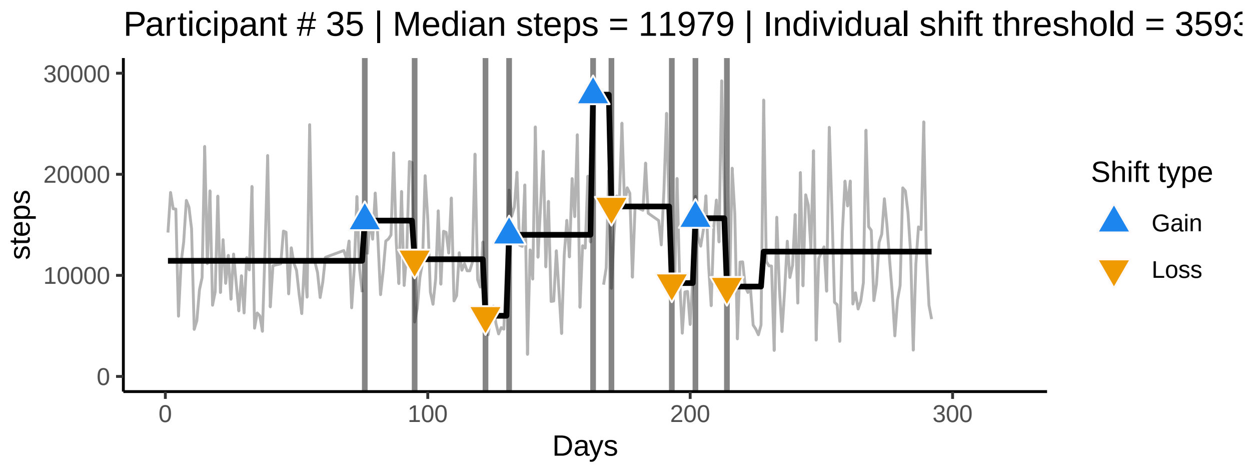
Participant # 35 | Median steps = 11979 | Individual shift threshold = 3593
30000
20000
10000
0
steps
0
100
200
300
Days
Shift type
Gain
Loss

```
##
## [[36]]
```

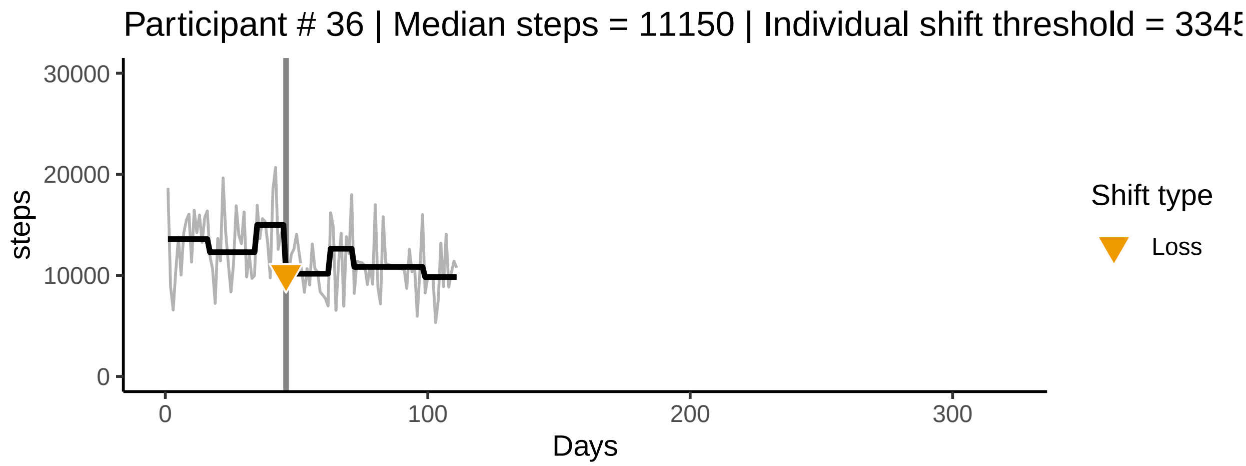
Participant # 36 | Median steps = 11150 | Individual shift threshold = 3345
30000
20000
10000
0
steps
0
100
200
300
Days
Shift type
Loss

```
##
## [[37]]
```

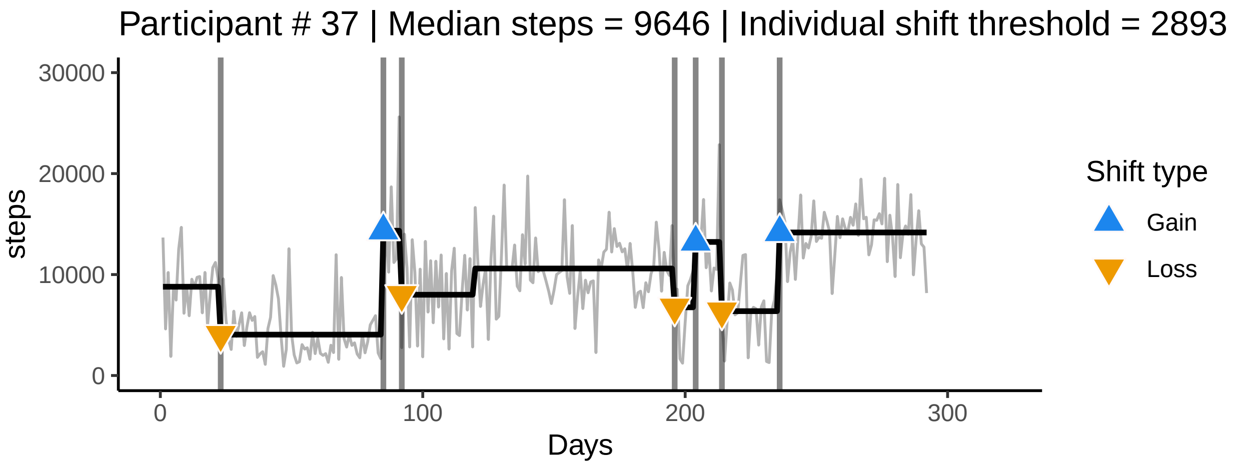

Participant # 37 | Median steps = 9646 | Individual shift threshold = 2893
30000
20000
10000
0
steps
0
100
200
300
Days
Shift type
Gain
Loss


```
##
## [[38]]
```

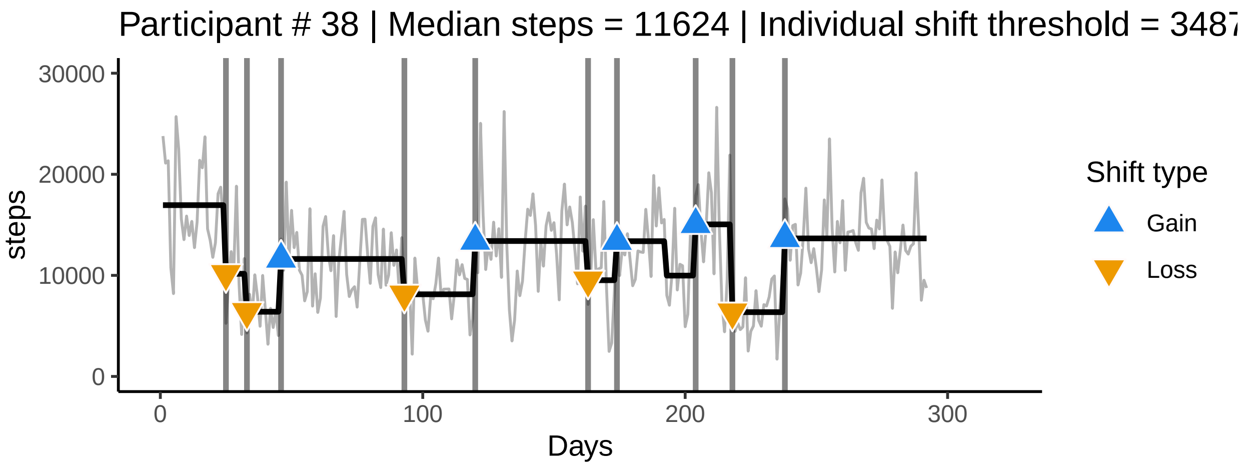

Participant # 38 | Median steps = 11624 | Individual shift threshold = 3487
30000
20000
10000
0
steps
0
100
200
300
Days
Shift type
Gain
Loss


```
##
## [[39]]
```

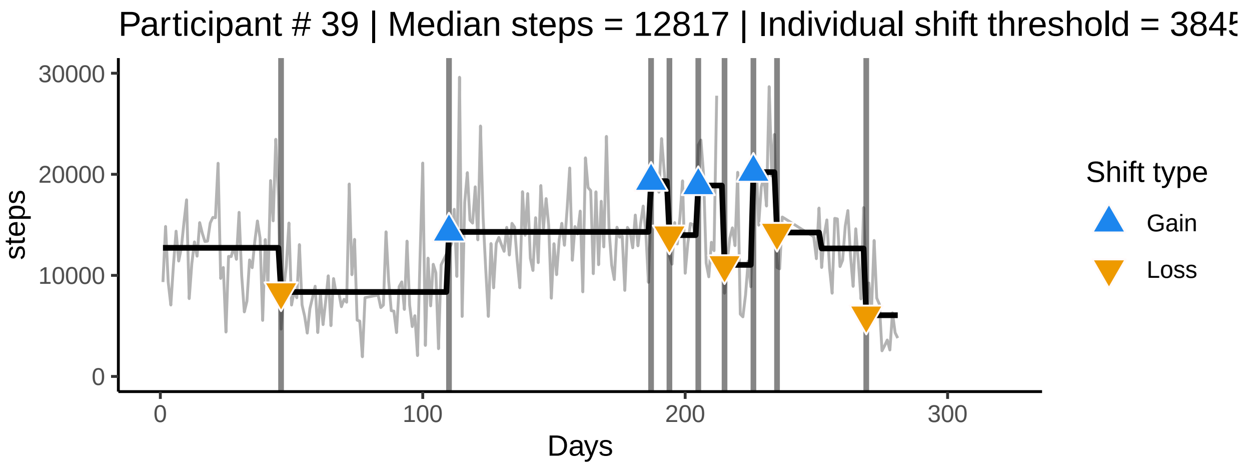

Participant # 39 | Median steps = 12817 | Individual shift threshold = 3845
30000
20000
10000
0
steps
0
100
200
300
Days
Shift type
Gain
Loss

```
##
## [[40]]
```

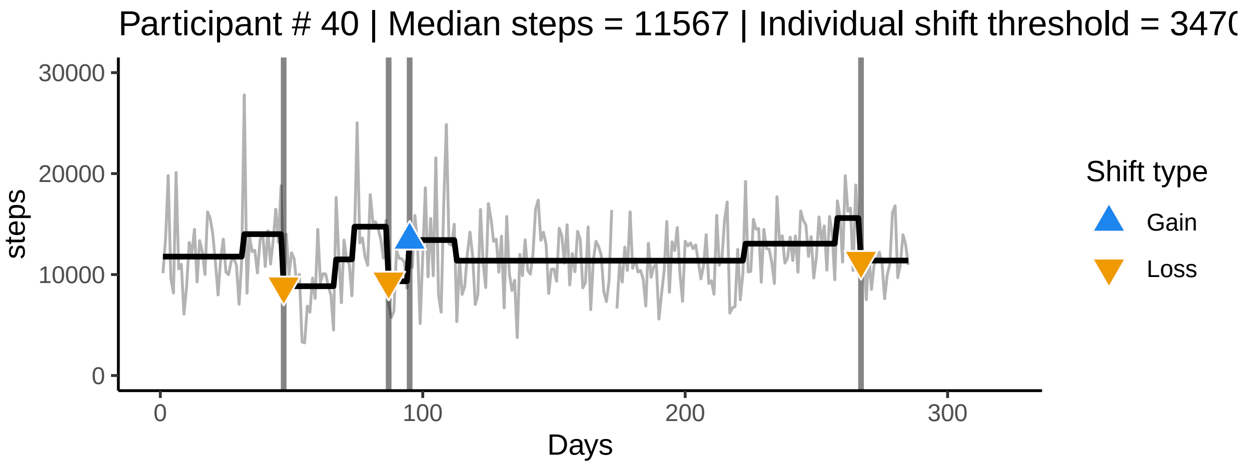
Participant # 40 | Median steps = 11567 | Individual shift threshold = 347(
30000
20000
10000
0
steps
0
100
200
300
Days
Shift type
Gain
Loss

```
##
## [[41]]
```

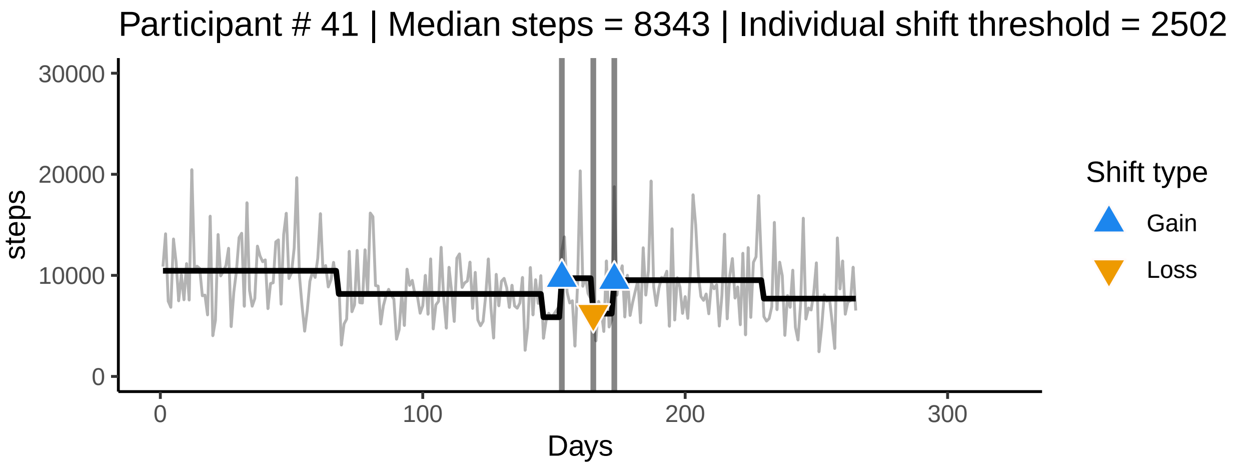
Participant # 41 | Median steps = 8343 | Individual shift threshold = 2502
30000
20000
10000
0
steps
0
100
200
300
Days
Shift type
Gain
Loss

```
##
## [[42]]
```

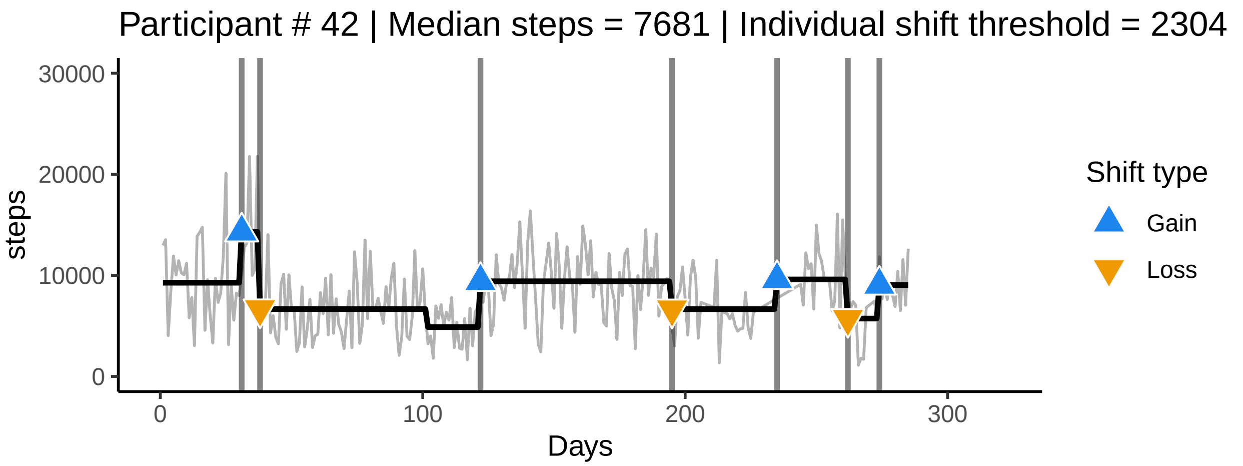
Participant # 42 | Median steps = 7681 | Individual shift threshold = 2304
30000
20000
10000
0
steps
0
100
200
300
Days
Shift type
Gain
Loss

```
##
## [[43]]
```

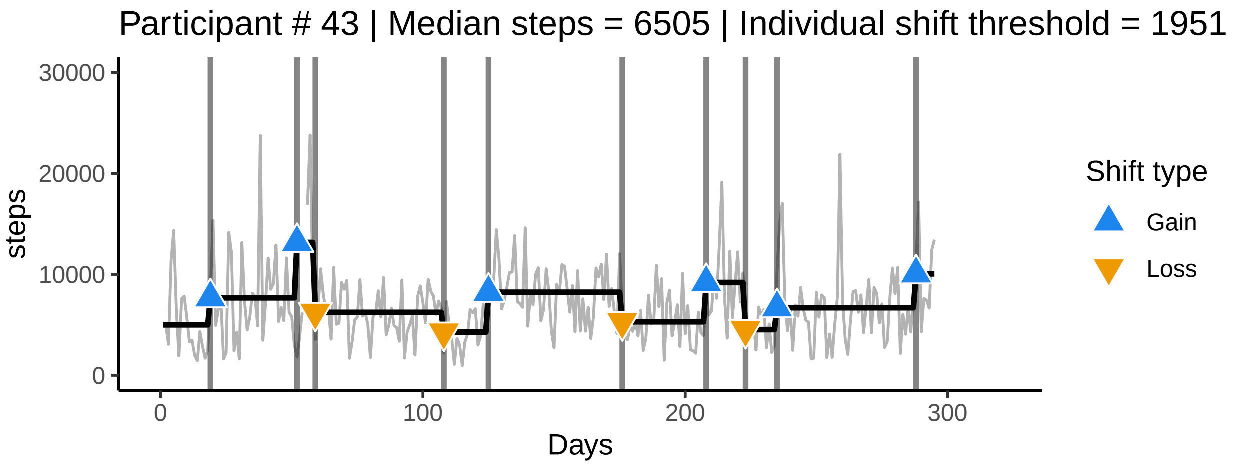

Participant # 43 | Median steps = 6505 | Individual shift threshold = 1951
steps
30000
20000
10000
0
0
100
200
300
Days
Shift type
Gain
Loss


```
##
## [[44]]
```

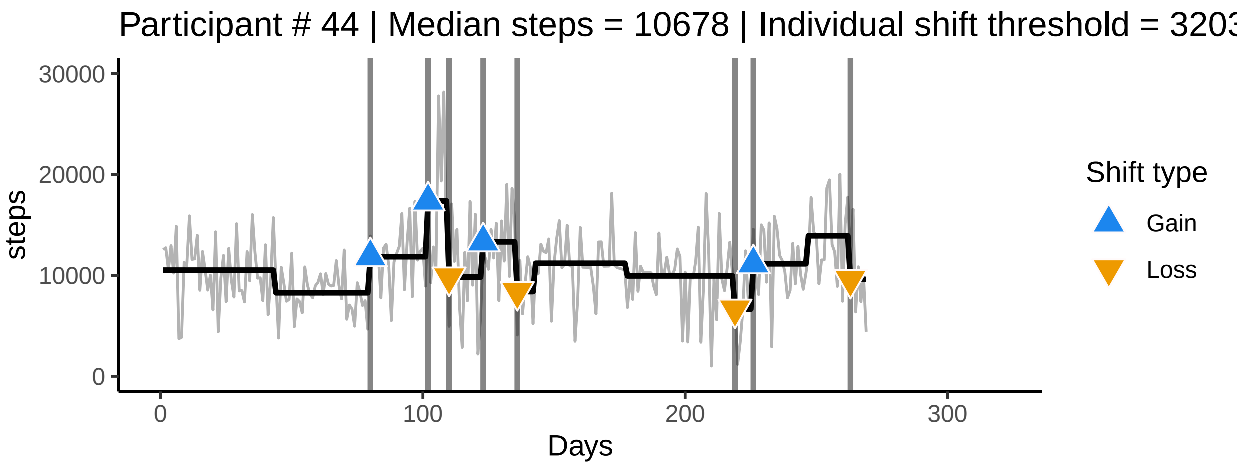

Participant # 44 | Median steps = 10678 | Individual shift threshold = 320
steps
30000
20000
10000
0
0
100
200
300
Days
Shift type
Gain
Loss


```
##
## [[45]]
```

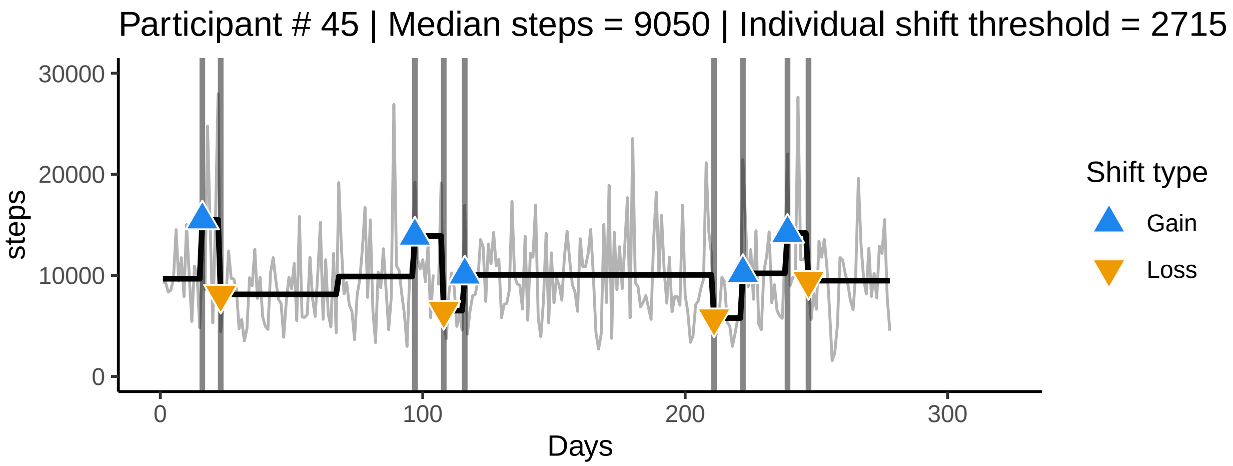

Participant # 45 | Median steps = 9050 | Individual shift threshold = 2715
steps
30000
20000
10000
0
0
100
200
300
Days
Shift type
Gain
Loss

```
##
## [[46]]
```

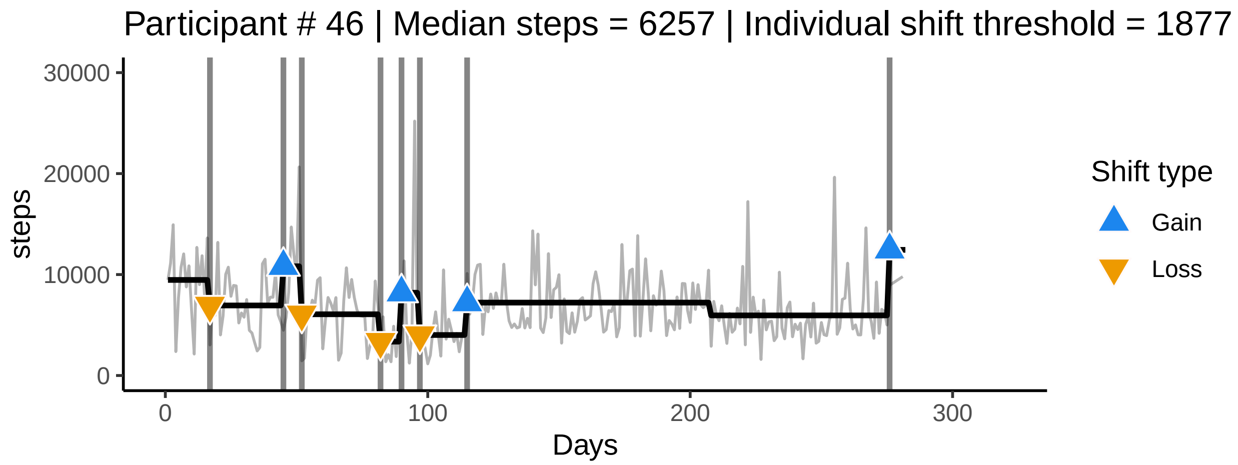
Participant # 46 | Median steps = 6257 | Individual shift threshold = 1877
30000
20000
10000
0
steps
0
100
200
300
Days
Shift type
Gain
Loss

```
##
## [[47]]
```

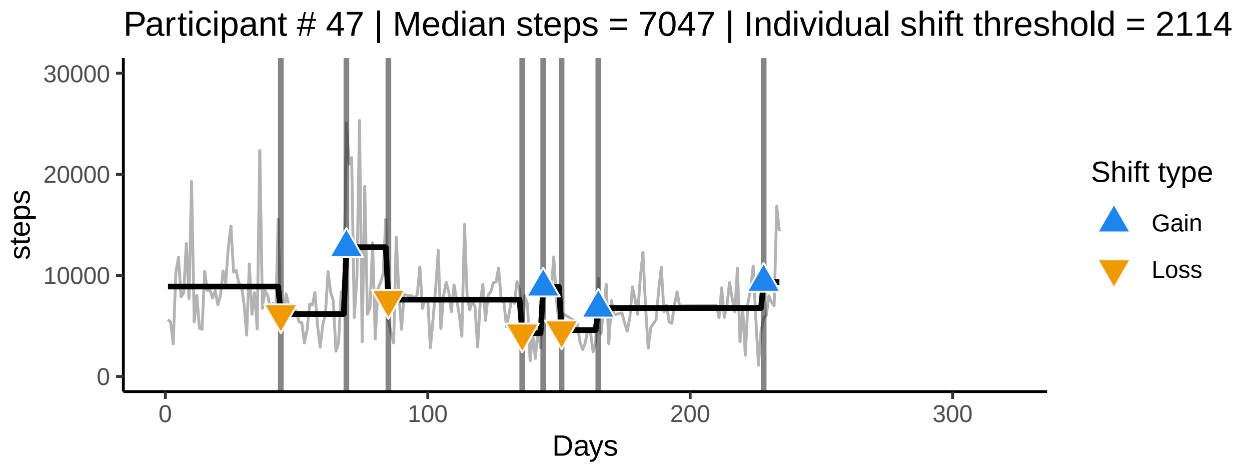
Participant # 47 | Median steps = 7047 | Individual shift threshold = 2114
30000
20000
10000
0
steps
0
100
200
300
Days
Shift type
Gain
Loss

```
##
## [[48]]
```

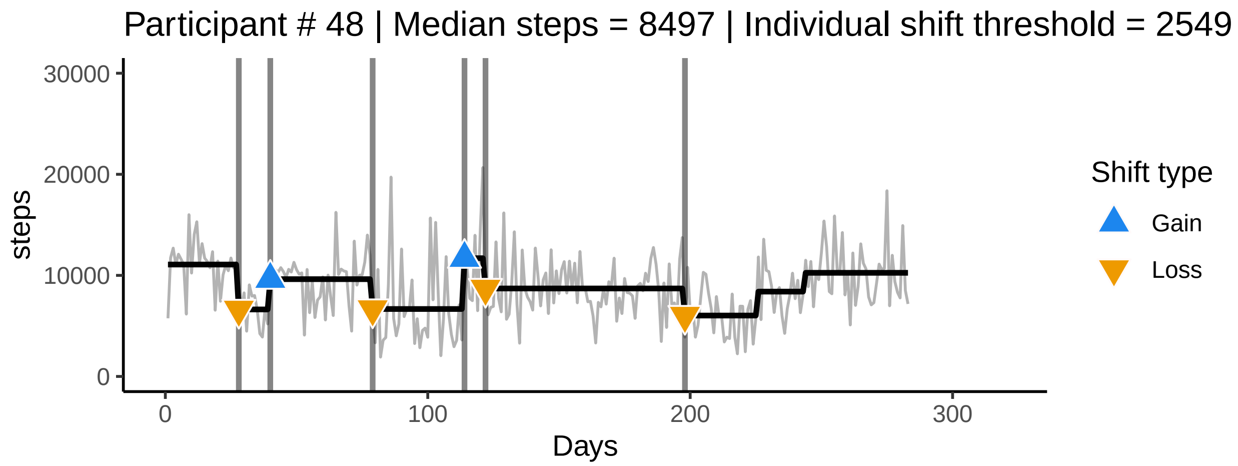
Participant # 48 | Median steps = 8497 | Individual shift threshold = 2549
30000
20000
10000
0
steps
0
100
200
300
Days
Shift type
Gain
Loss

```
##
## [[49]]
```

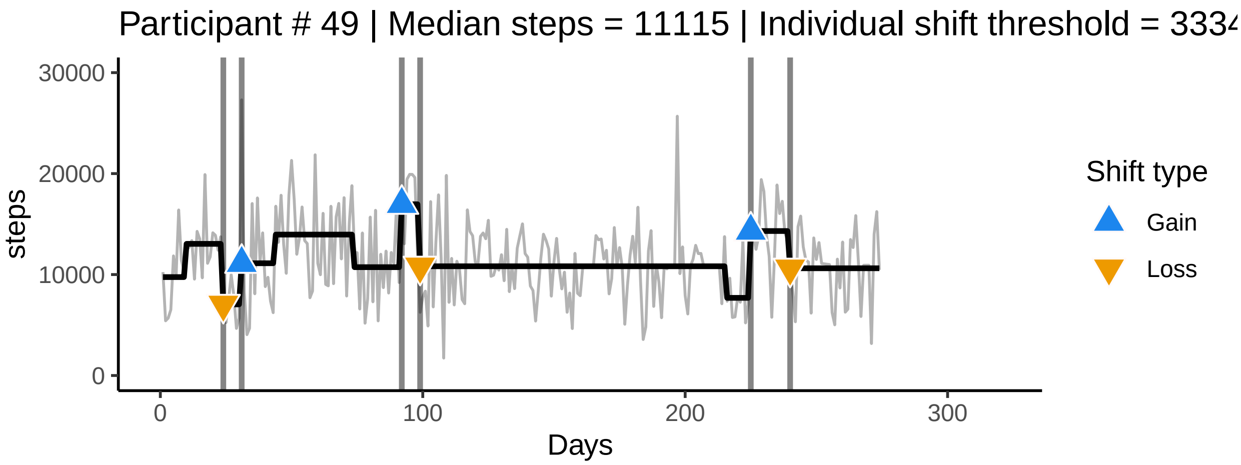

Participant # 49 | Median steps = 11115 | Individual shift threshold = 333
30000
20000
10000
0
steps
0
100
200
300
Days
Shift type
Gain
Loss


```
##
## [[50]]
```

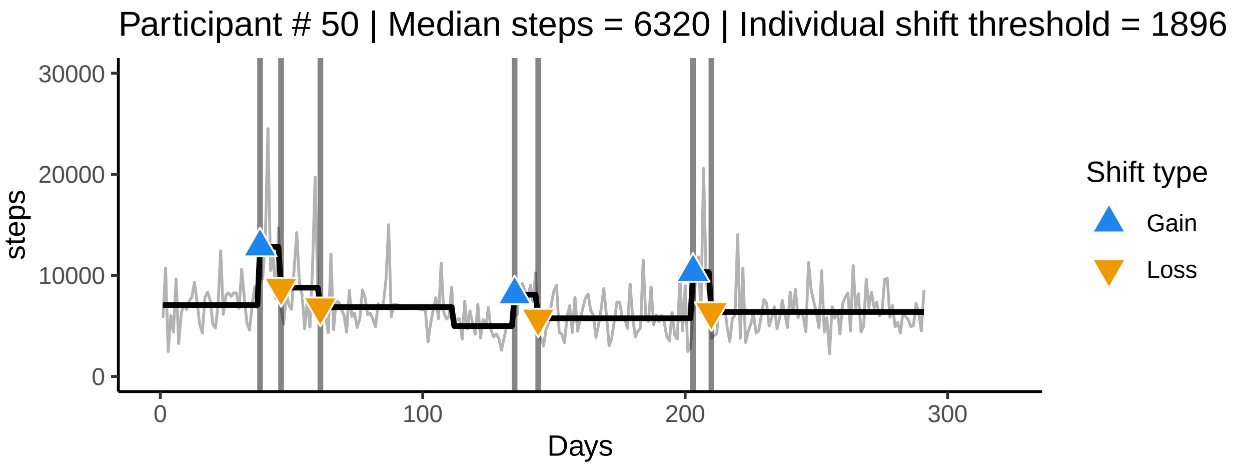

Participant # 50 | Median steps = 6320 | Individual shift threshold = 1896
30000
20000
10000
0
steps
0
100
200
300
Days
Shift type
Gain
Loss


```
##
## [[51]]
```

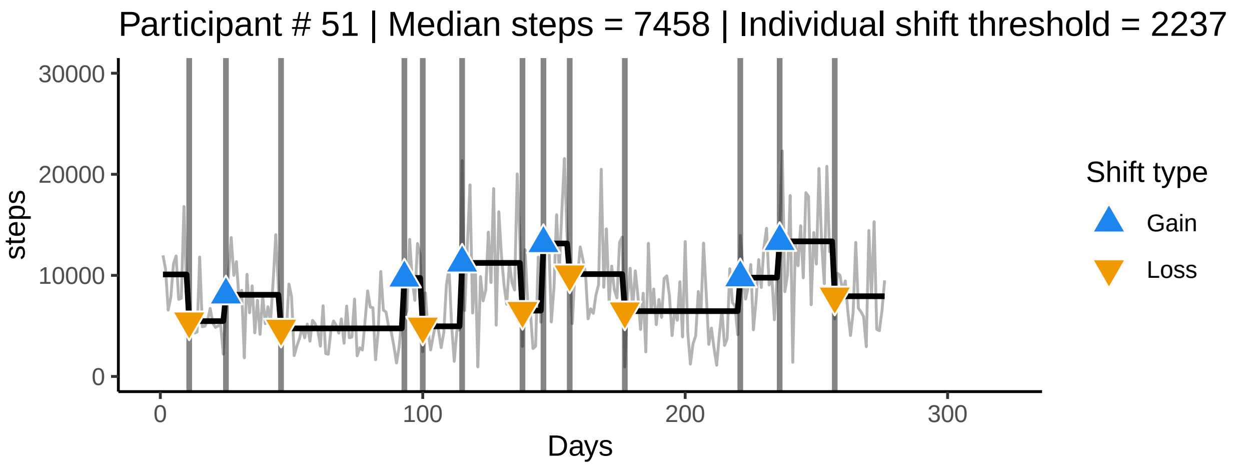

Participant # 51 | Median steps = 7458 | Individual shift threshold = 2237
30000
20000
10000
0
steps
0
100
200
300
Days
Shift type
Gain
Loss

```
##
## [[52]]
```

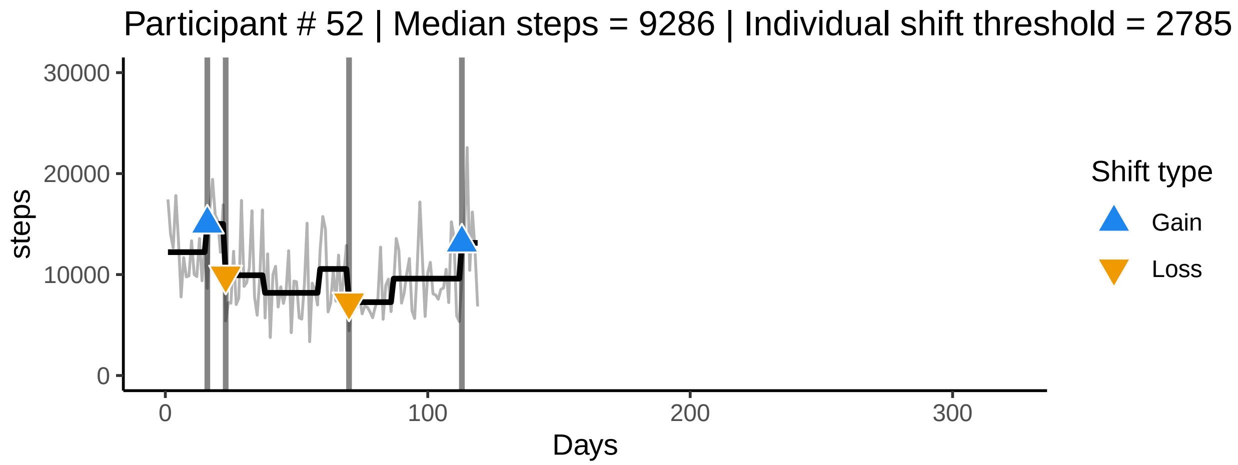

Participant # 52 | Median steps = 9286 | Individual shift threshold = 2785
steps
30000
20000
10000
0
0
100
200
300
Days
Shift type
Gain
Loss


```
##
## [[53]]
```

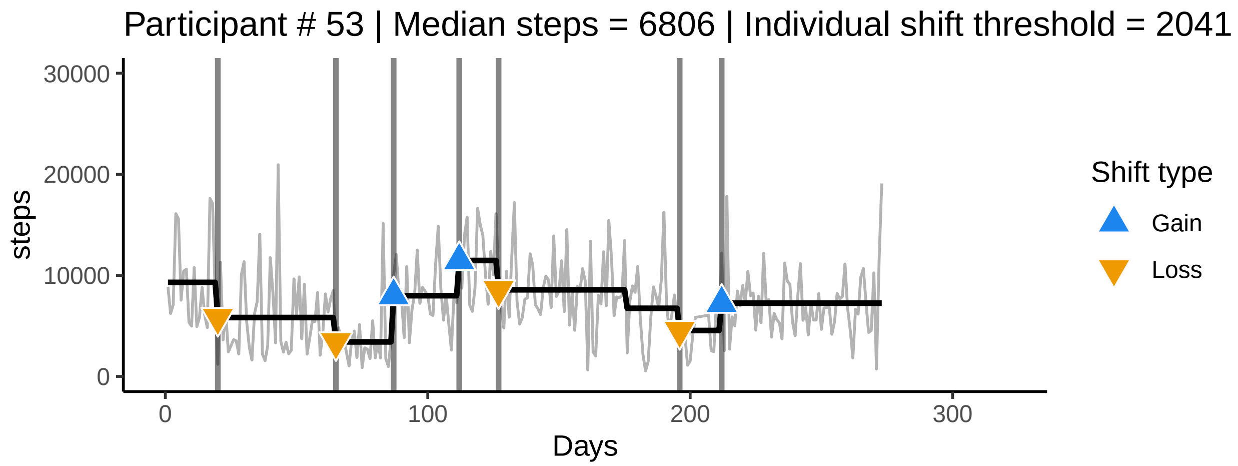

Participant # 53 | Median steps = 6806 | Individual shift threshold = 2041
steps
30000
20000
10000
0
0
100
200
300
Days
Shift type
Gain
Loss


```
##
## [[54]]
```

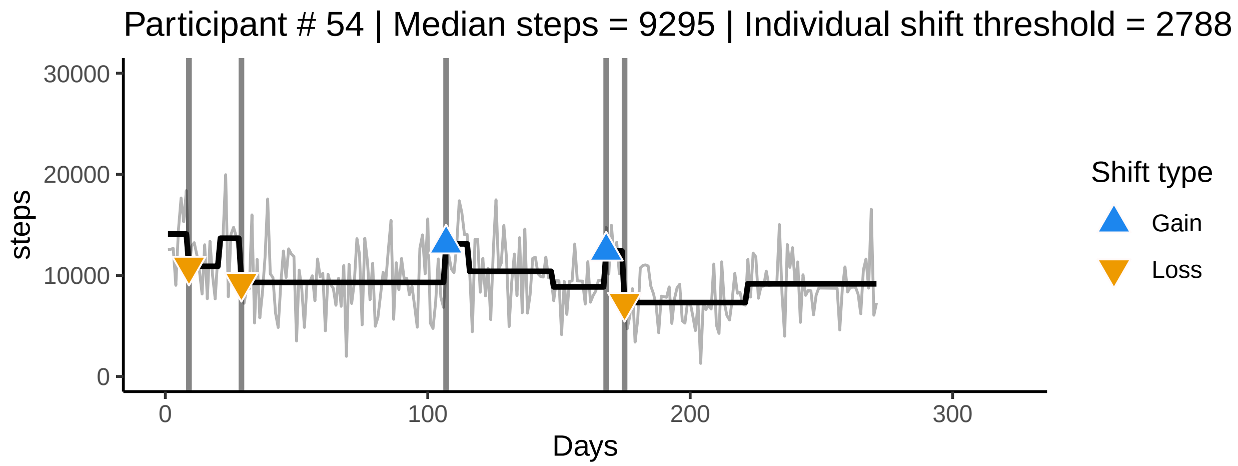

Participant # 54 | Median steps = 9295 | Individual shift threshold = 2788
steps
30000
20000
10000
0
0
100
200
300
Days
Shift type
Gain
Loss

```
##
## [[55]]
```

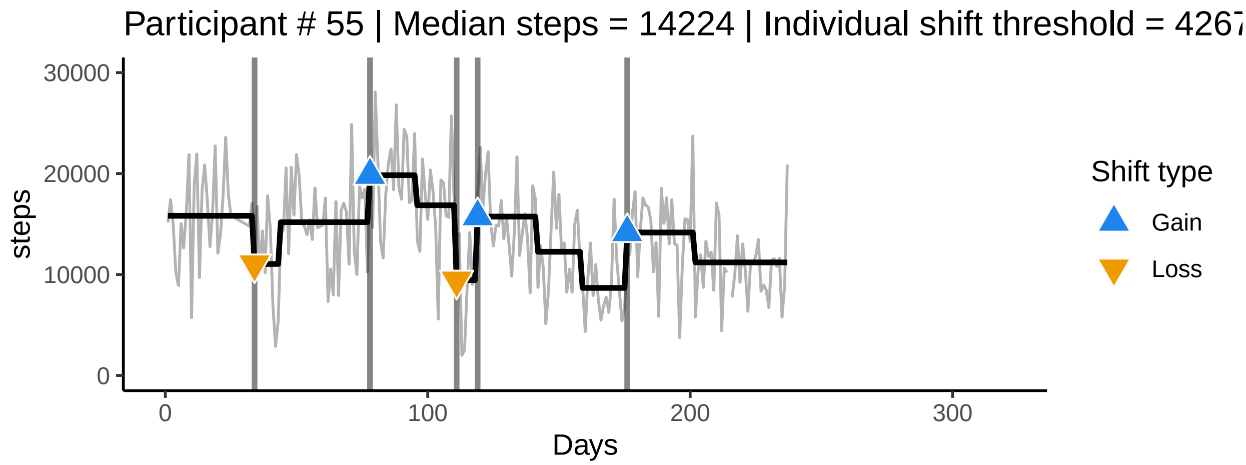

Participant # 55 | Median steps = 14224 | Individual shift threshold = 426
steps
30000
20000
10000
0
0
100
200
300
Days
Shift type
Gain
Loss


```
##
## [[56]]
```

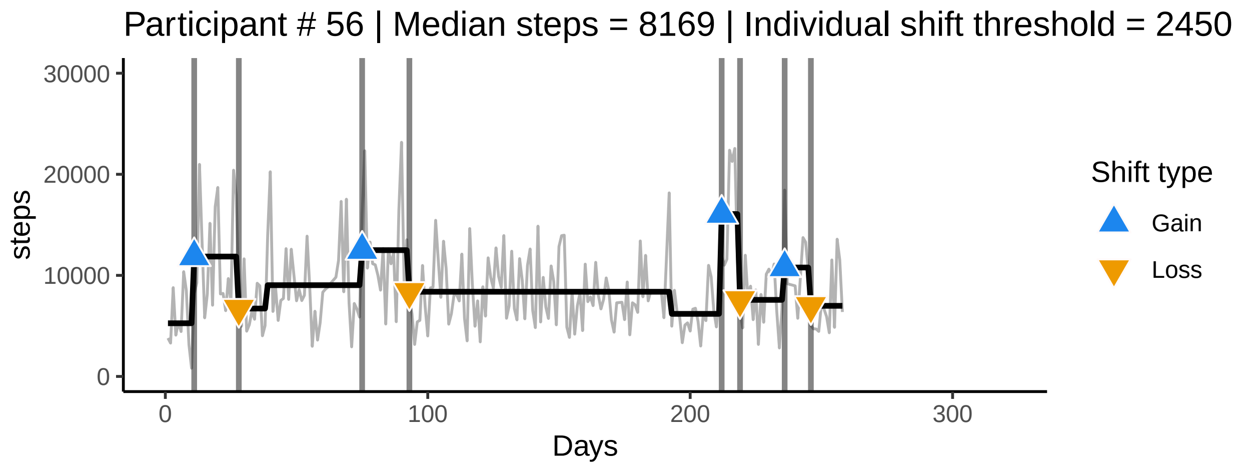

Participant # 56 | Median steps = 8169 | Individual shift threshold = 2450
steps
30000
20000
10000
0
0
100
200
300
Days
Shift type
Gain
Loss


```
##
## [[57]]
```

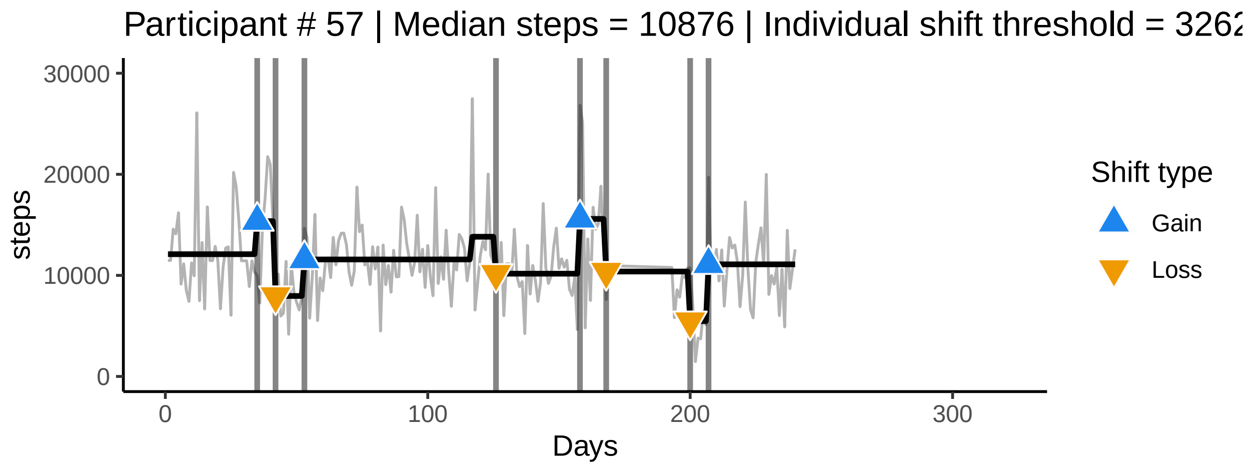

Participant # 57 | Median steps = 10876 | Individual shift threshold = 326
steps
30000
20000
10000
0
0
100
200
300
Days
Shift type
Gain
Loss

```
##
## [[58]]
```

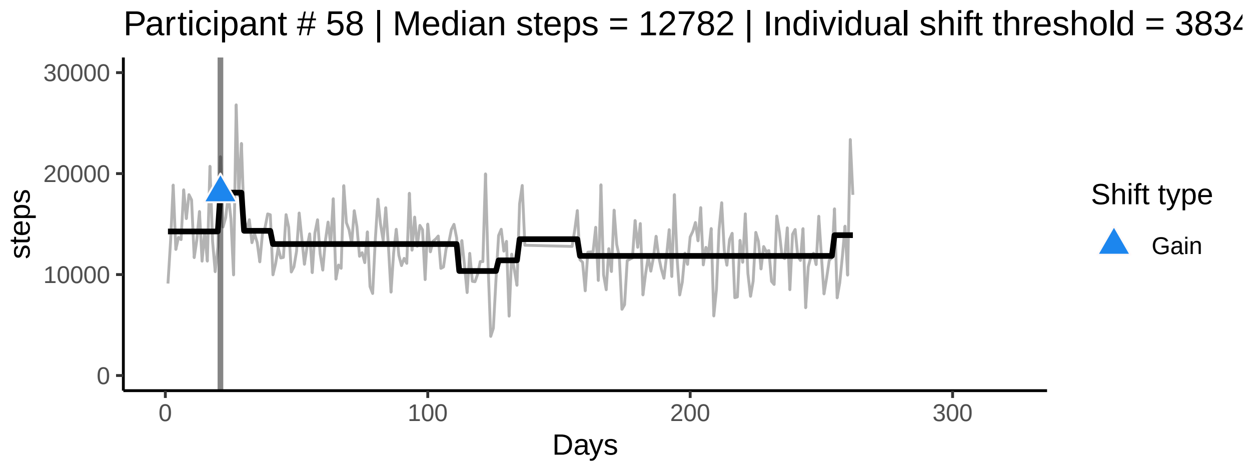
Participant # 58 | Median steps = 12782 | Individual shift threshold = 383
steps
30000
20000
10000
0
0
100
200
300
Days
Shift type
Gain

```
##
## [[59]]
```

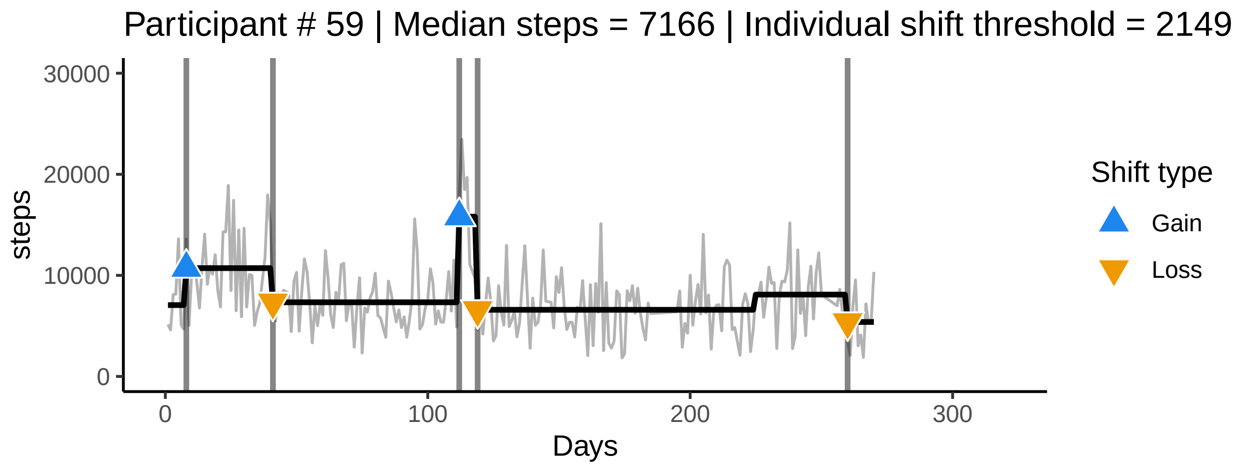
Participant # 59 | Median steps = 7166 | Individual shift threshold = 2149
steps
30000
20000
10000
0
0
100
200
300
Days
Shift type
Gain
Loss

```
##
## [[60]]
```

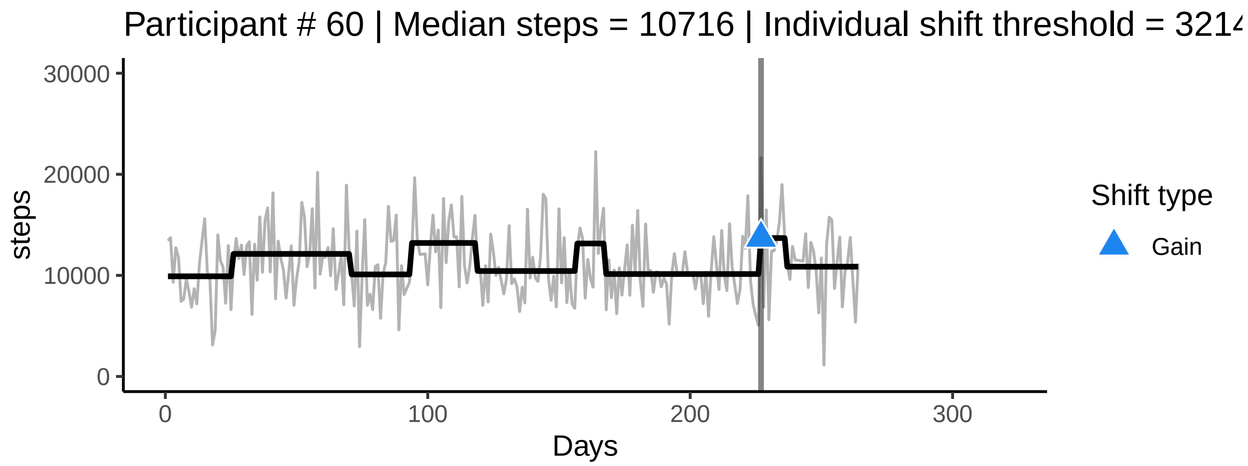
Participant # 60 | Median steps = 10716 | Individual shift threshold = 321
steps
30000
20000
10000
0
0
100
200
300
Days
Shift type
Gain

```
##
## [[61]]
```

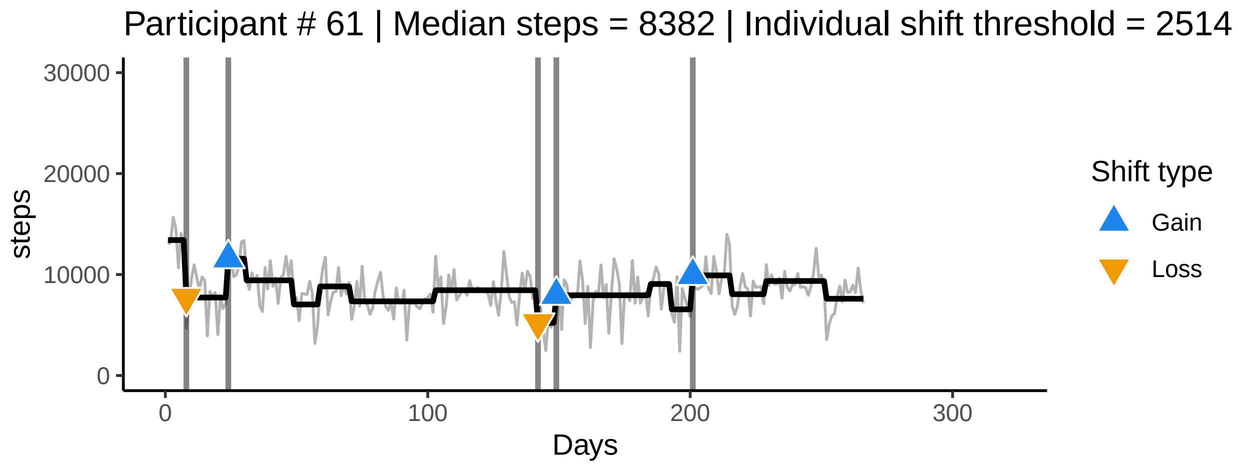

Participant # 61 | Median steps = 8382 | Individual shift threshold = 2514
steps
30000
20000
10000
0
0
100
200
300
Days
Shift type
Gain
Loss


```
##
## [[62]]
```

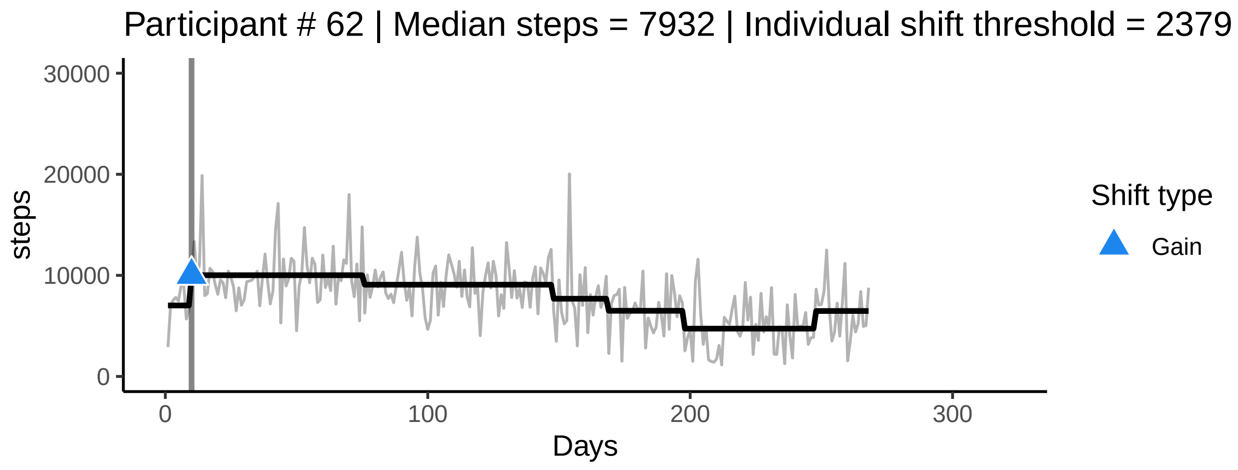

Participant # 62 | Median steps = 7932 | Individual shift threshold = 2379
steps
30000
20000
10000
0
0
100
200
300
Days
Shift type
Gain


```
##
## [[63]]
```

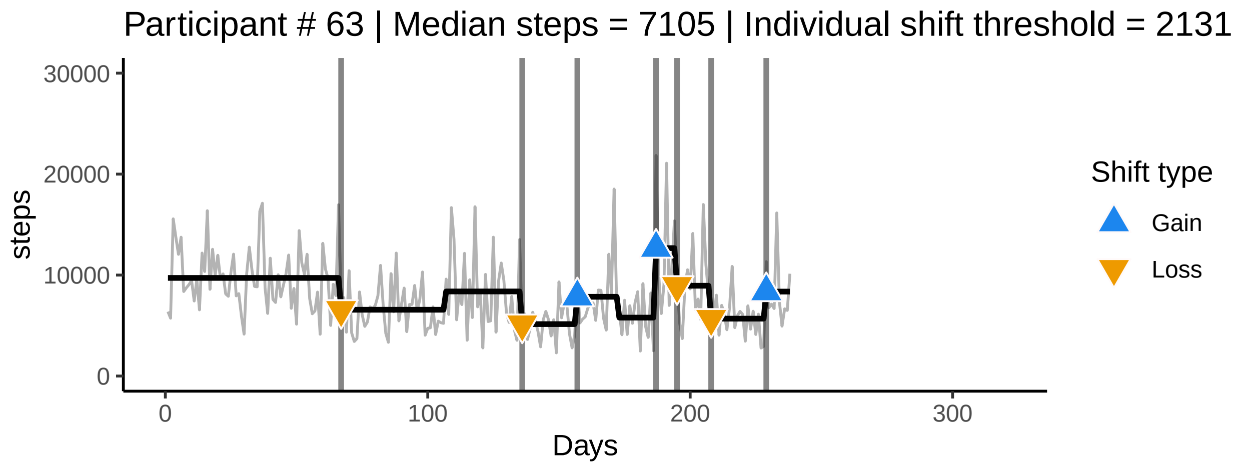

Participant # 63 | Median steps = 7105 | Individual shift threshold = 2131
steps
30000
20000
10000
0
0
100
200
300
Days
Shift type
Gain
Loss

```
##
## [[64]]
```

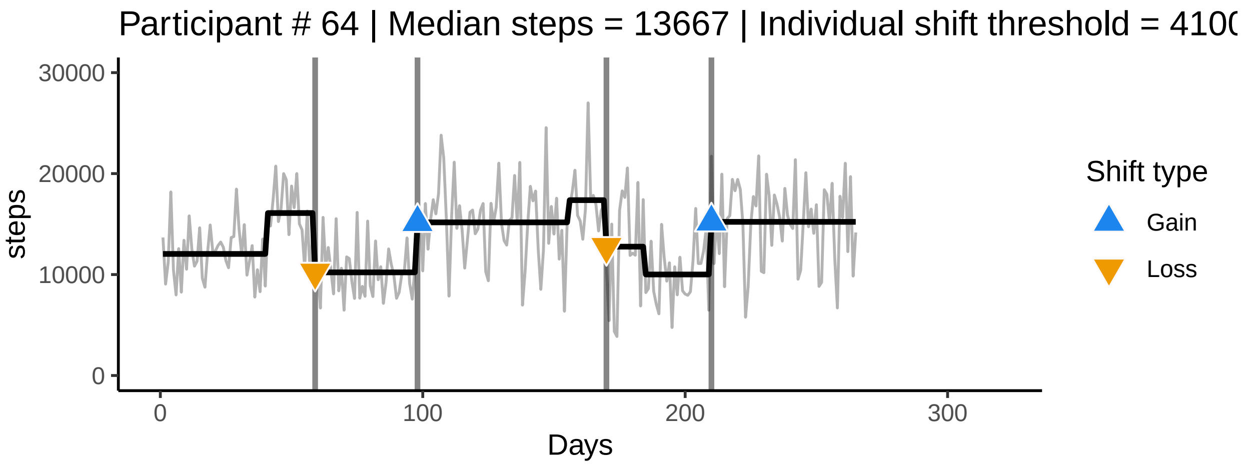
Participant # 64 | Median steps = 13667 | Individual shift threshold = 410(
steps
Days
Shift type
Gain
Loss

```
##
## [[65]]
```

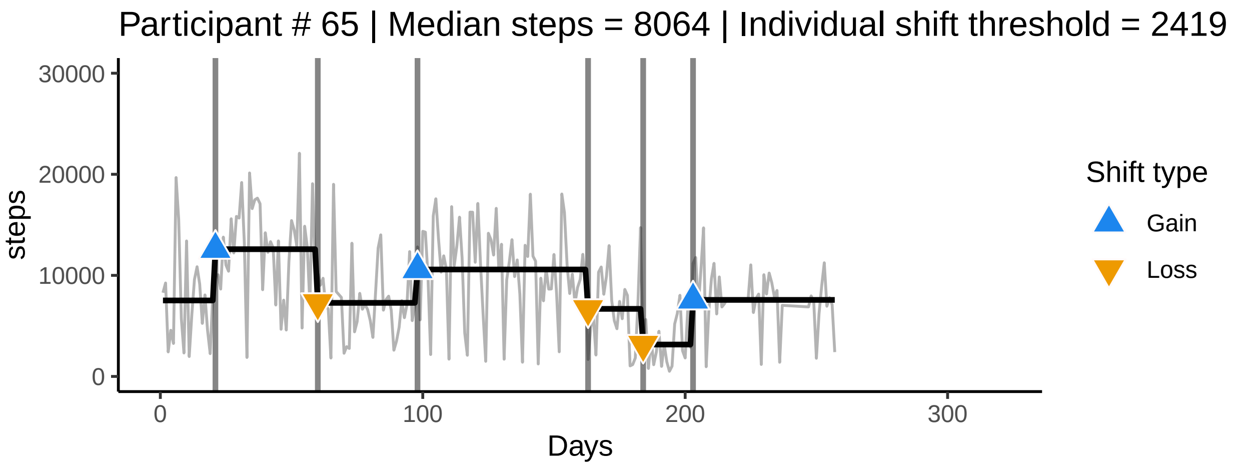
Participant # 65 | Median steps = 8064 | Individual shift threshold = 2419
steps
Days
Shift type
Gain
Loss

```
##
## [[66]]
```

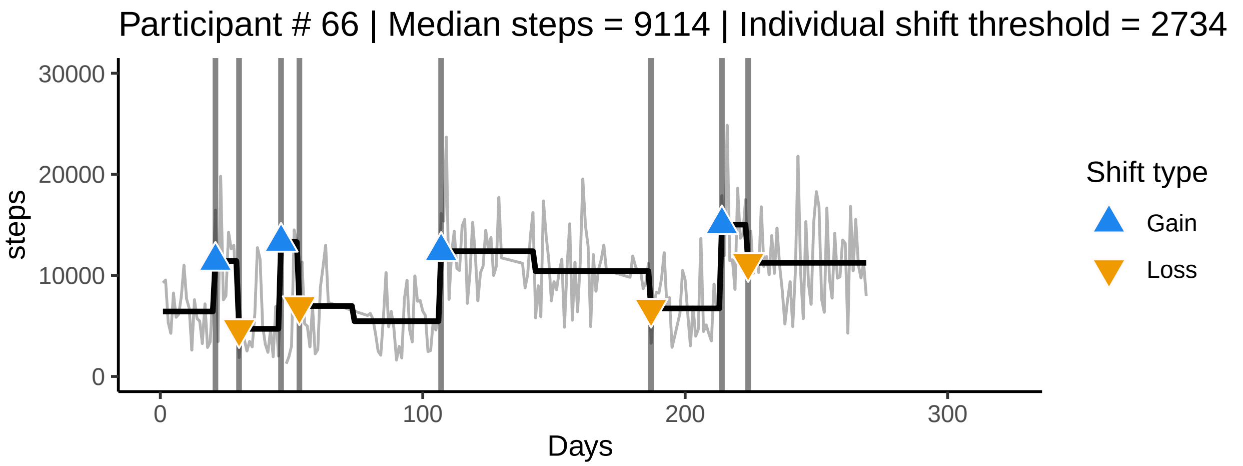
Participant # 66 | Median steps = 9114 | Individual shift threshold = 2734
steps
Days
Shift type
Gain
Loss

```
##
## [[67]]
```

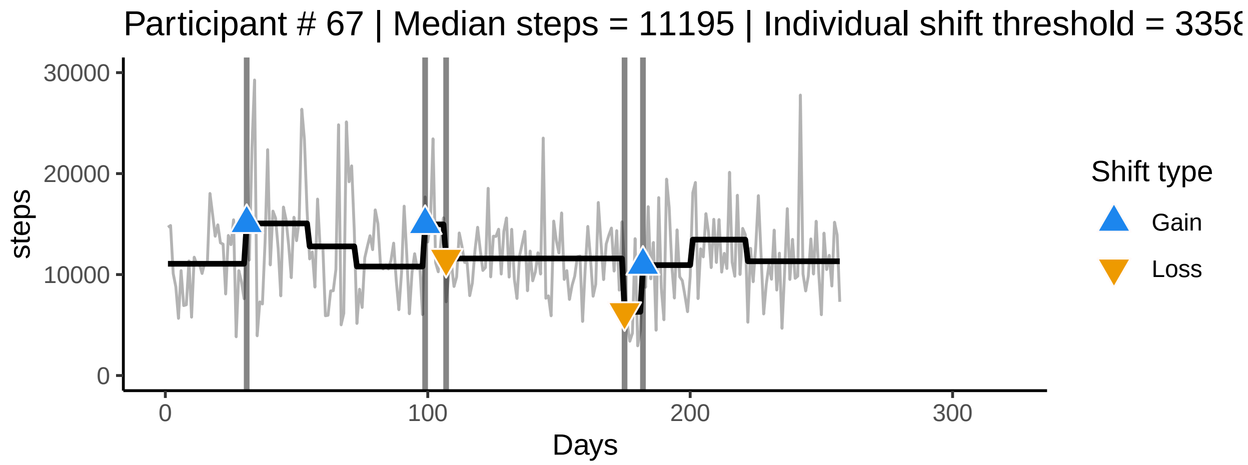
Participant # 67 | Median steps = 11195 | Individual shift threshold = 335
steps
30000
20000
10000
0
0
100
200
300
Days
Shift type
Gain
Loss

```
##
## [[68]]
```

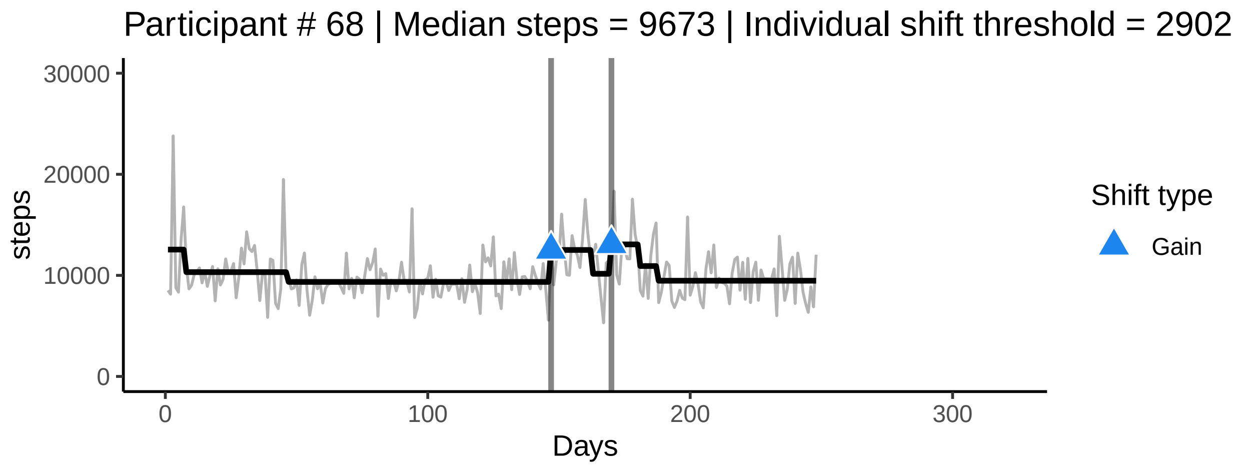
Participant # 68 | Median steps = 9673 | Individual shift threshold = 2902
steps
30000
20000
10000
0
0
100
200
300
Days
Shift type
Gain

```
##
## [[69]]
```

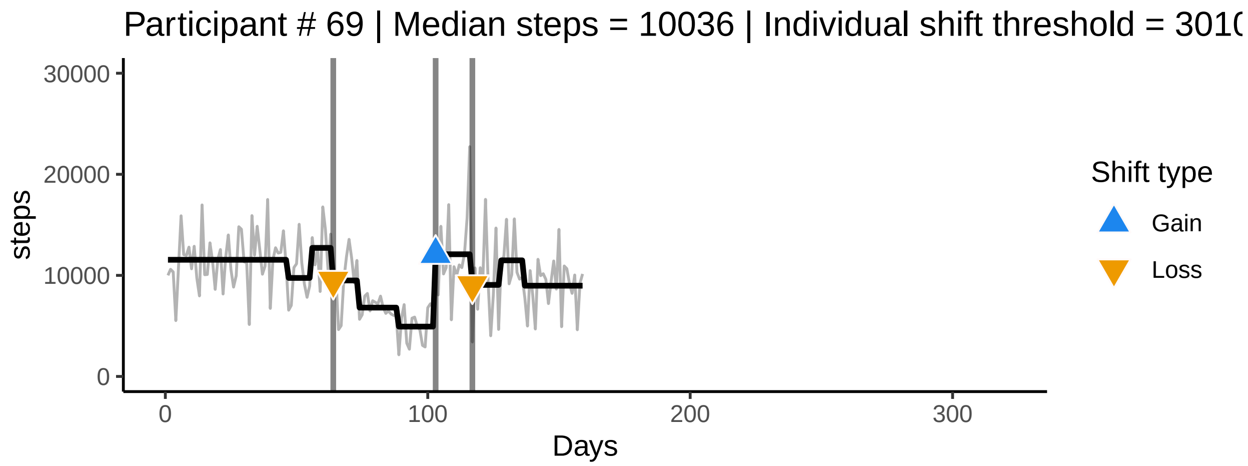
Participant # 69 | Median steps = 10036 | Individual shift threshold = 301
steps
30000
20000
10000
0
0
100
200
300
Days
Shift type
Gain
Loss

```
##
## [[70]]
```

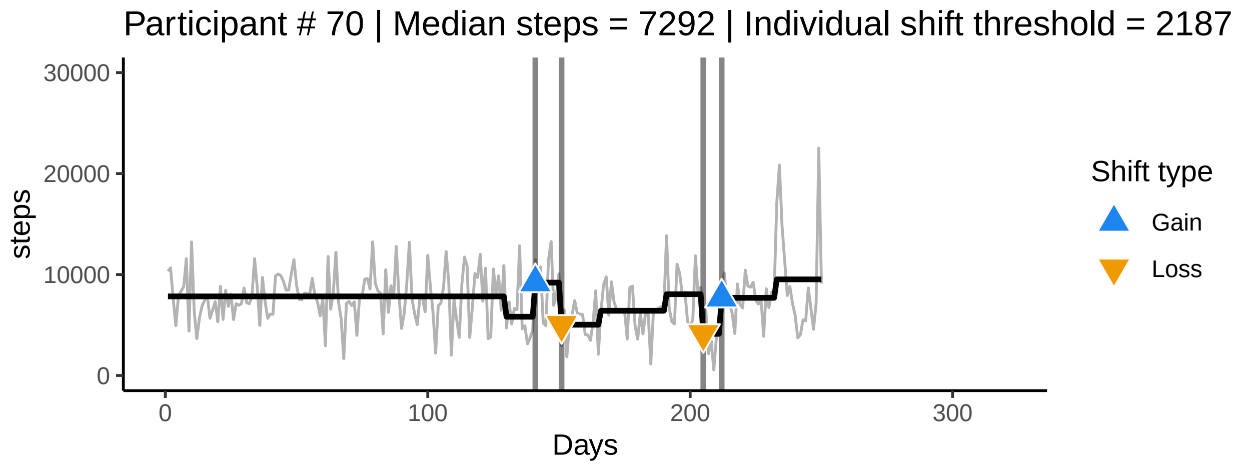
Participant # 70 | Median steps = 7292 | Individual shift threshold = 2187
steps
30000
20000
10000
0
0
100
200
300
Days
Shift type
Gain
Loss

```
##
## [[71]]
```

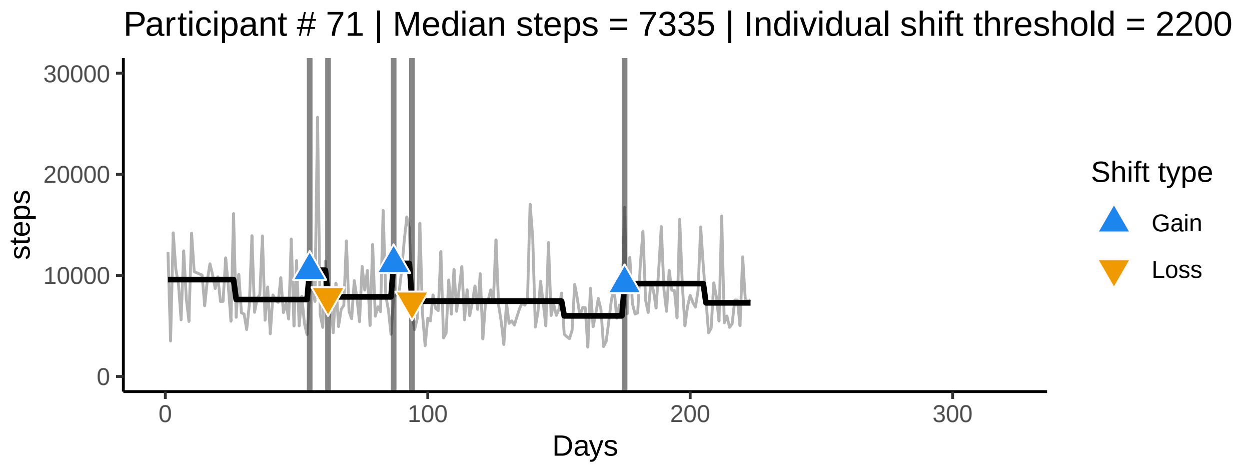
Participant # 71 | Median steps = 7335 | Individual shift threshold = 2200
steps
30000
20000
10000
0
0
100
200
300
Days
Shift type
Gain
Loss

```
##
## [[72]]
```

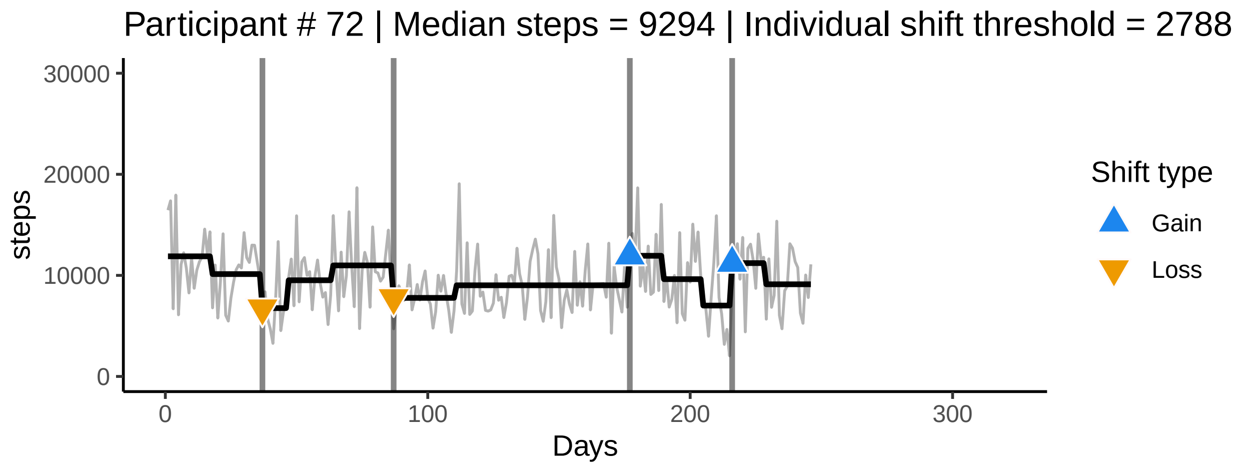
Participant # 72 | Median steps = 9294 | Individual shift threshold = 2788
steps
30000
20000
10000
0
0
100
200
300
Days
Shift type
Gain
Loss

```
##
## [[73]]
```

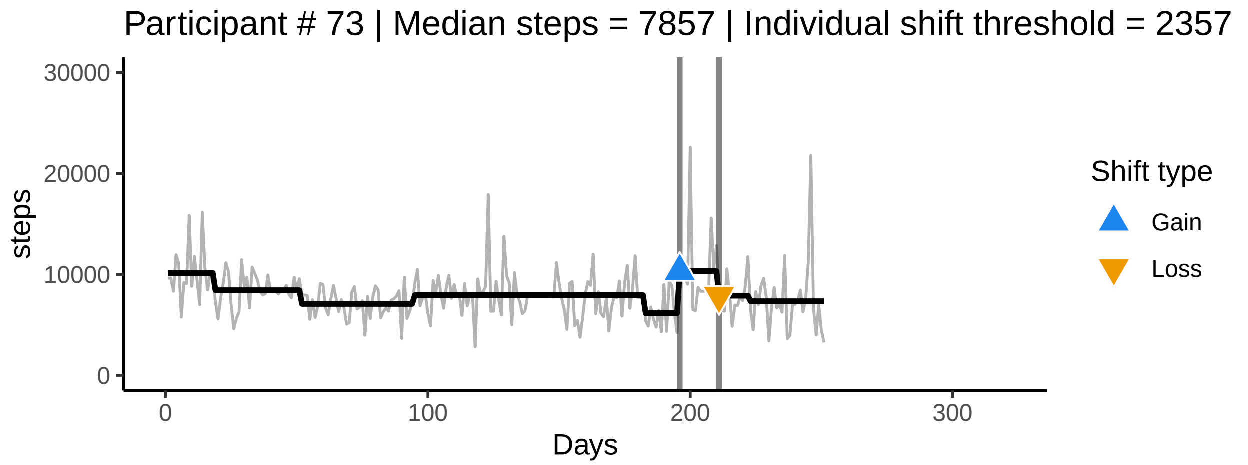

Participant # 73 | Median steps = 7857 | Individual shift threshold = 2357
30000
20000
10000
0
steps
0
100
200
300
Days
Shift type
Gain
Loss


```
##
## [[74]]
```

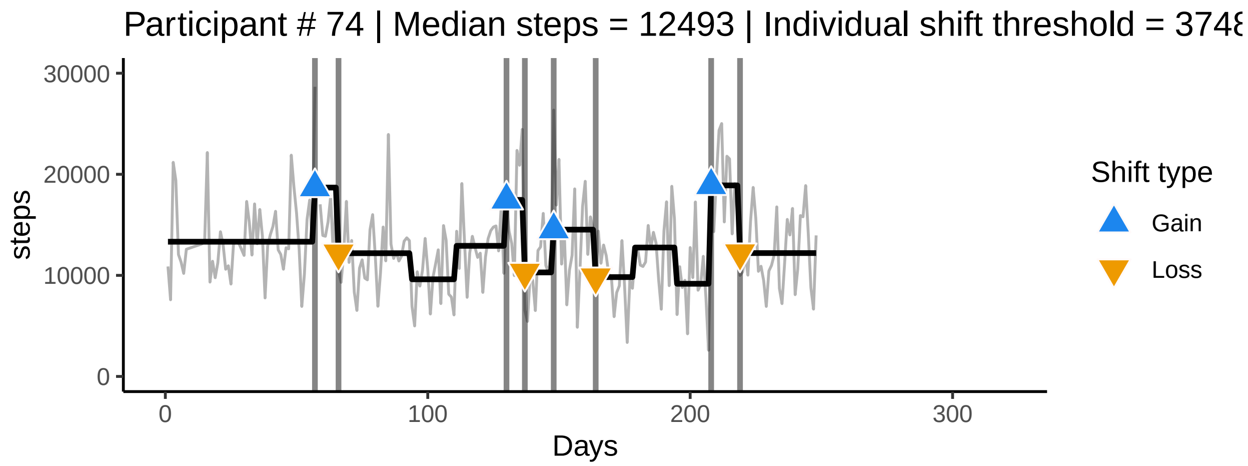

Participant # 74 | Median steps = 12493 | Individual shift threshold = 374
30000
20000
10000
0
steps
0
100
200
300
Days
Shift type
Gain
Loss


```
##
## [[75]]
```

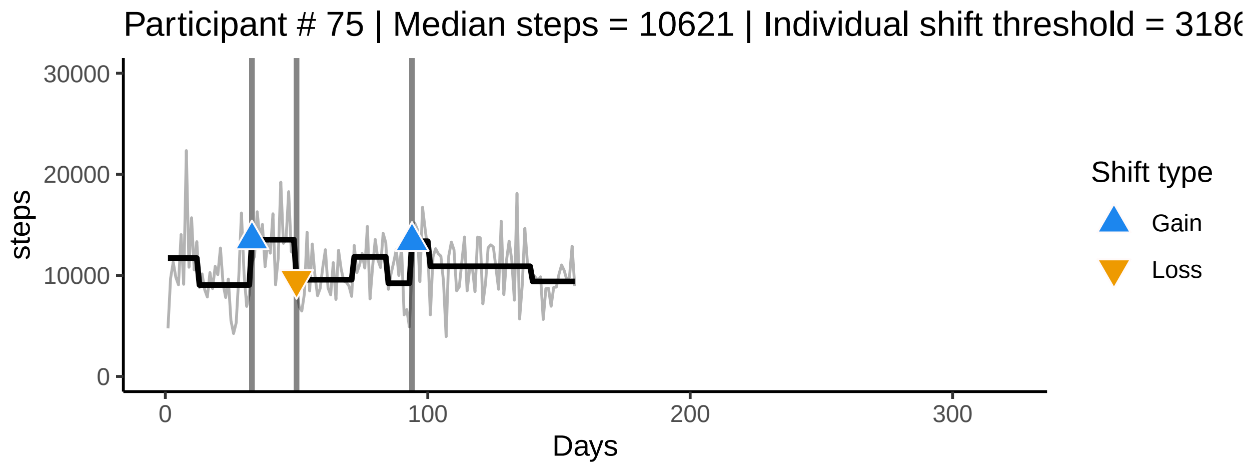

Participant # 75 | Median steps = 10621 | Individual shift threshold = 318
30000
20000
10000
0
steps
0
100
200
300
Days
Shift type
Gain
Loss

```
##
## [[76]]
```

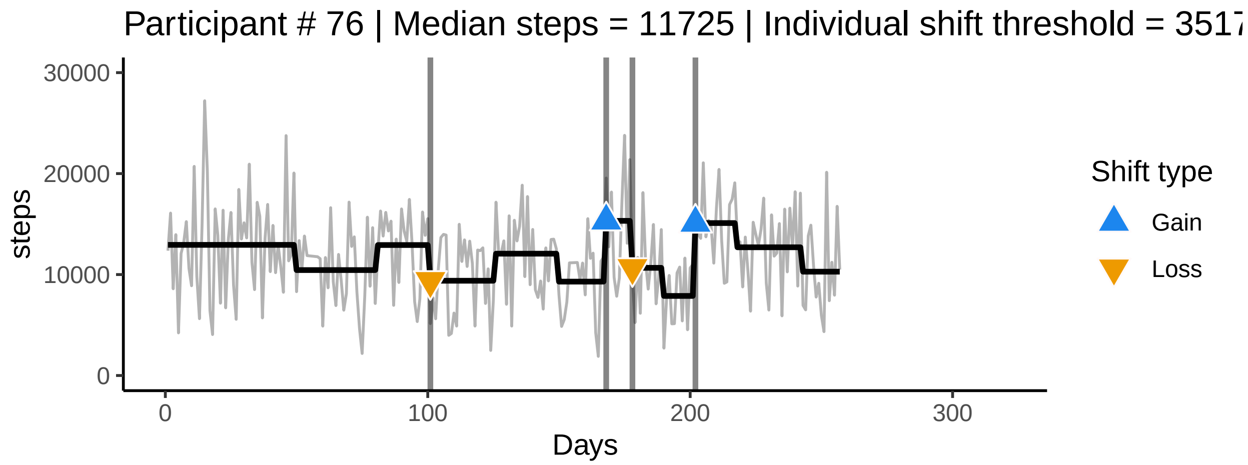

Participant # 76 | Median steps = 11725 | Individual shift threshold = 3517
steps
30000
20000
10000
0
0
100
200
300
Days
Shift type
Gain
Loss


```
##
## [[77]]
```

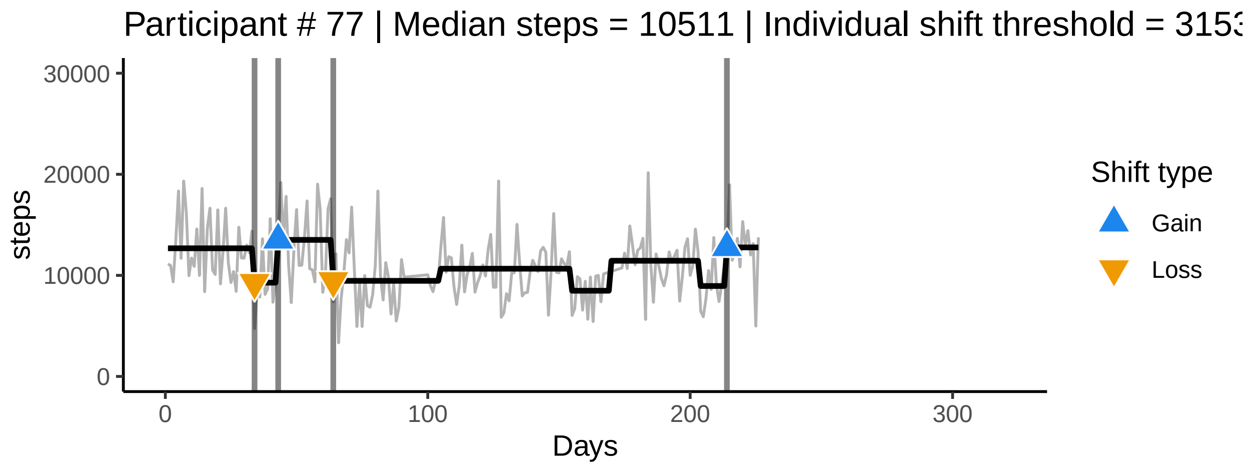

Participant # 77 | Median steps = 10511 | Individual shift threshold = 3153
steps
30000
20000
10000
0
0
100
200
300
Days
Shift type
Gain
Loss


```
##
## [[78]]
```

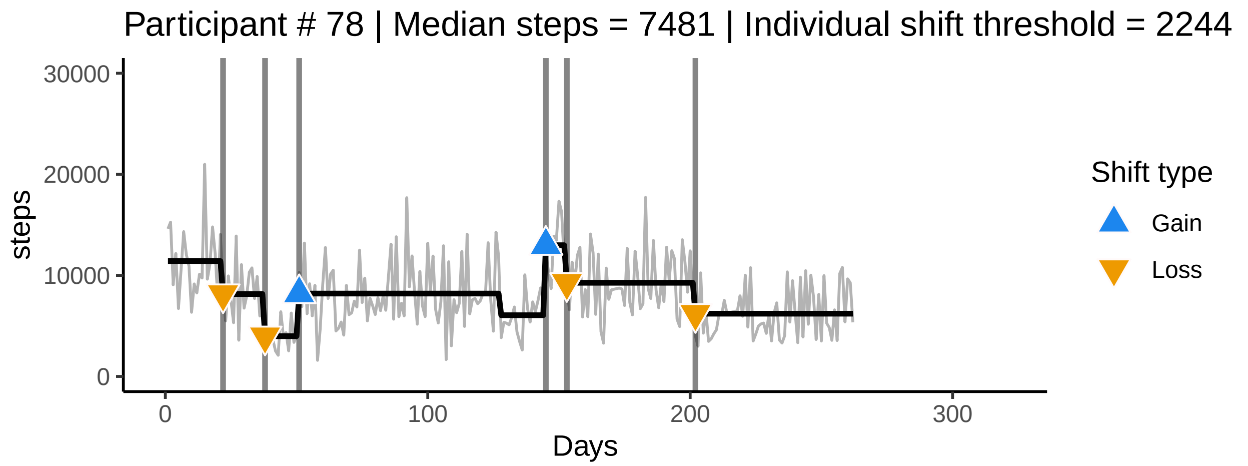

Participant # 78 | Median steps = 7481 | Individual shift threshold = 2244
steps
30000
20000
10000
0
0
100
200
300
Days
Shift type
Gain
Loss

```
##
## [[79]]
```

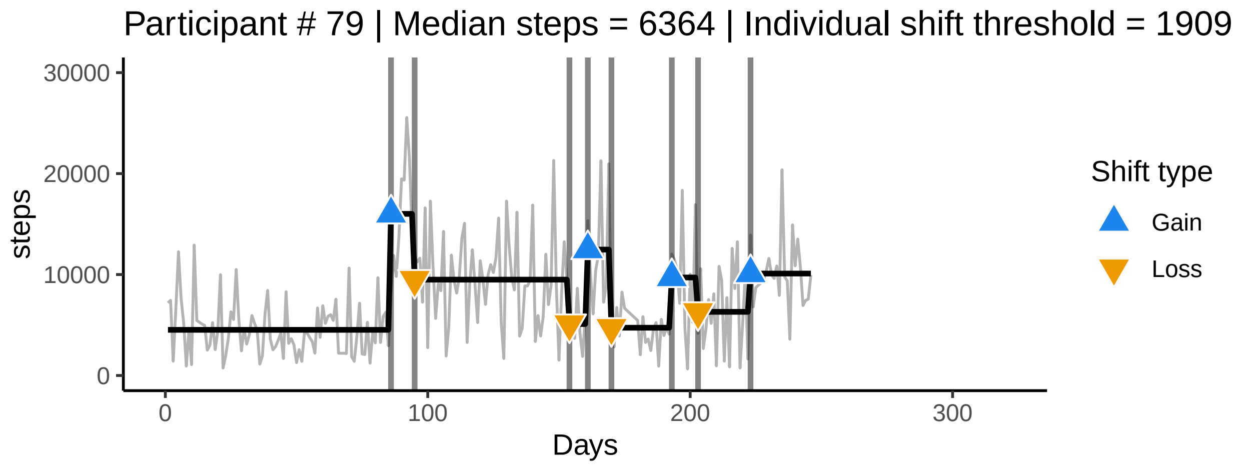

Participant # 79 | Median steps = 6364 | Individual shift threshold = 1909
30000
20000
10000
0
steps
0
100
200
300
Days
Shift type
Gain
Loss


```
##
## [[80]]
```

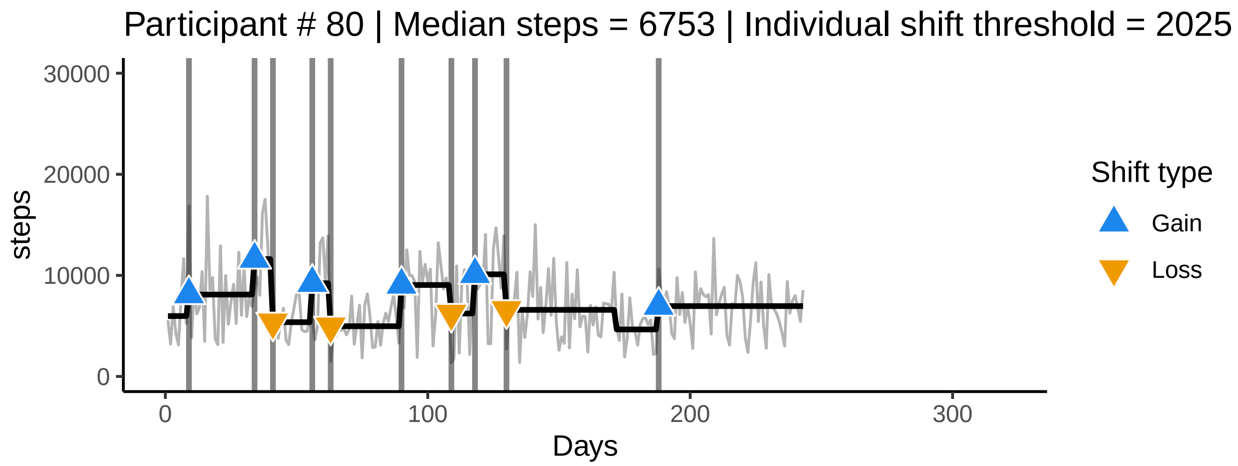

Participant # 80 | Median steps = 6753 | Individual shift threshold = 2025
30000
20000
10000
0
steps
0
100
200
300
Days
Shift type
Gain
Loss


```
##
## [[81]]
```

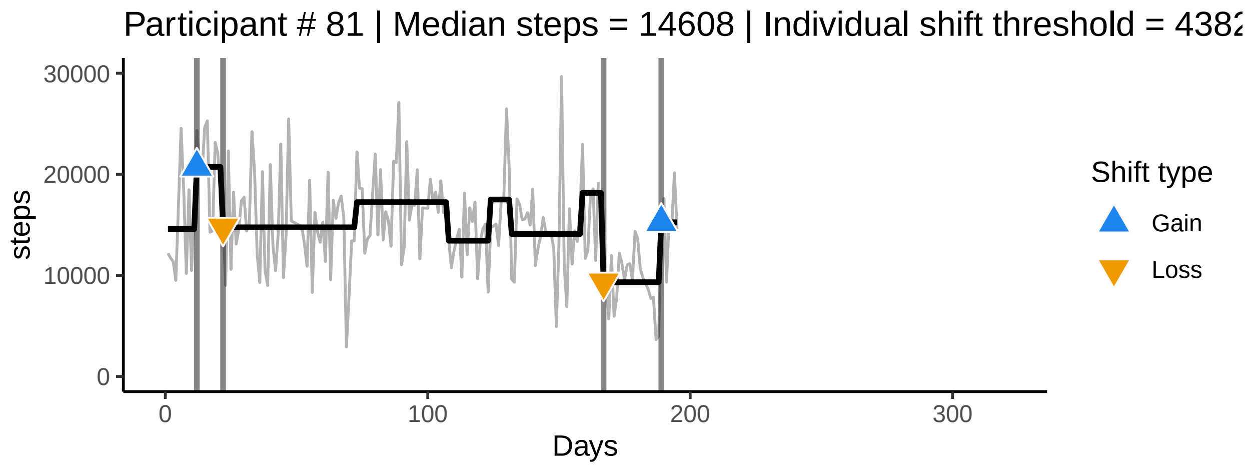

Participant # 81 | Median steps = 14608 | Individual shift threshold = 438
30000
20000
10000
0
steps
0
100
200
300
Days
Shift type
Gain
Loss

```
##
## [[82]]
```

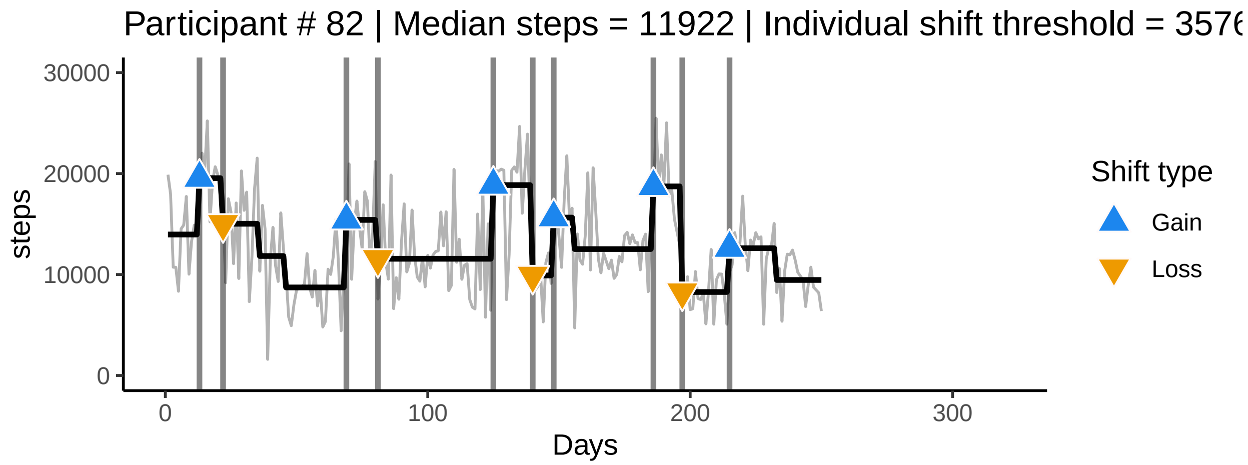
Participant # 82 | Median steps = 11922 | Individual shift threshold = 3576
30000
20000
10000
0
steps
0
100
200
300
Days
Shift type
Gain
Loss

```
##
## [[83]]
```

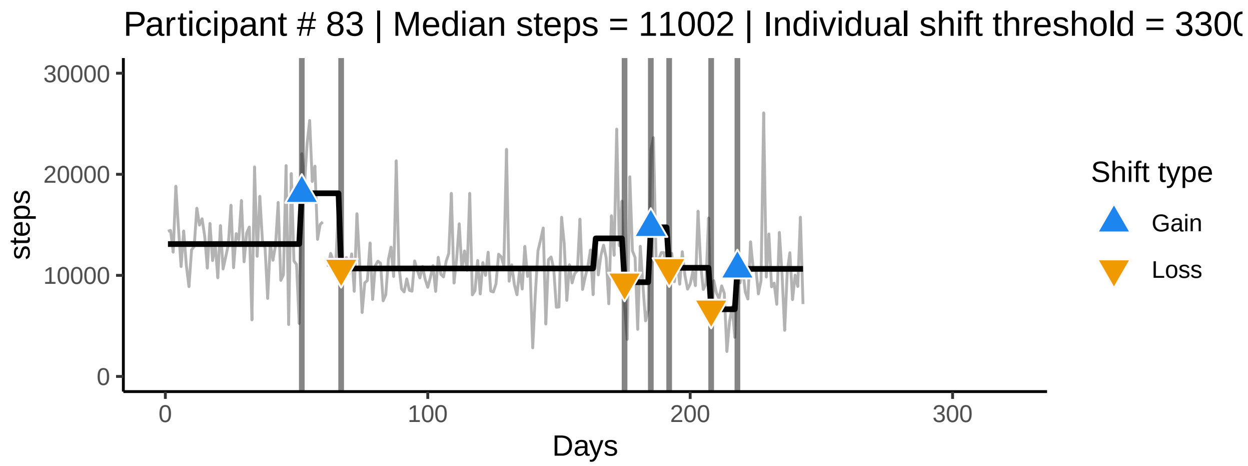
Participant # 83 | Median steps = 11002 | Individual shift threshold = 3300
30000
20000
10000
0
steps
0
100
200
300
Days
Shift type
Gain
Loss

```
##
## [[84]]
```

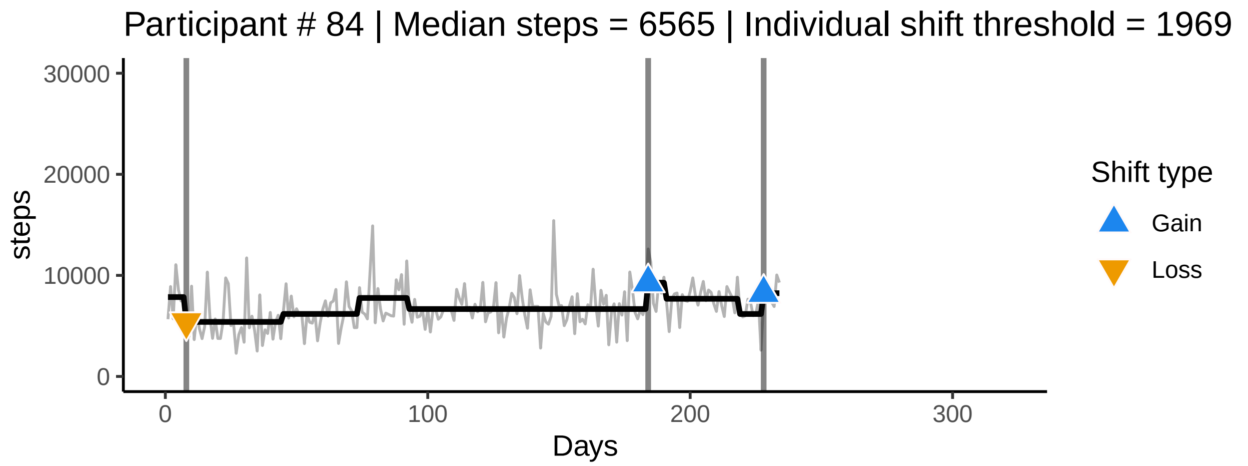
Participant # 84 | Median steps = 6565 | Individual shift threshold = 1969
30000
20000
10000
0
steps
0
100
200
300
Days
Shift type
Gain
Loss

```
##
## [[85]]
```

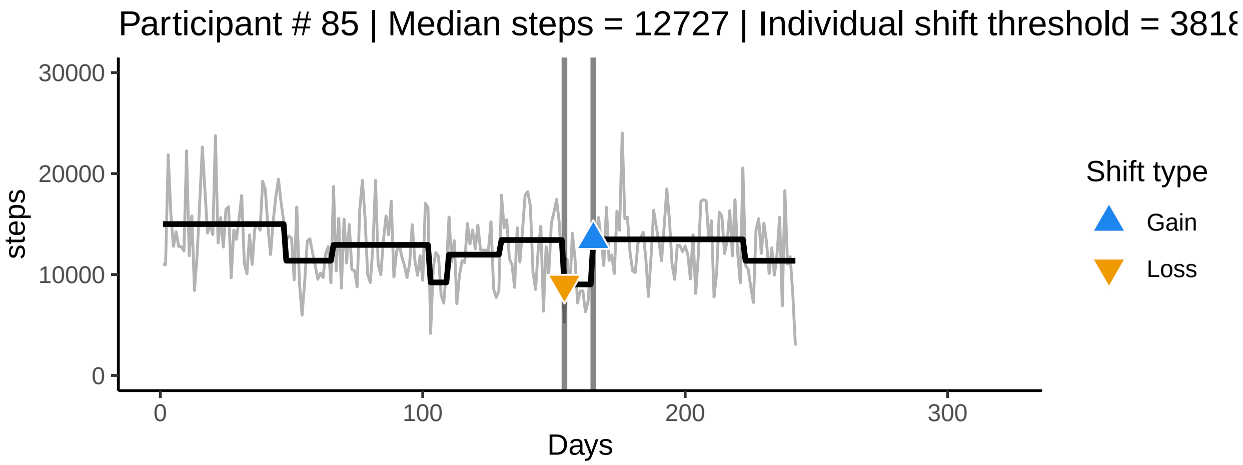

Participant # 85 | Median steps = 12727 | Individual shift threshold = 381
30000
20000
10000
0
steps
0
100
200
300
Days
Shift type
Gain
Loss


```
##
## [[86]]
```

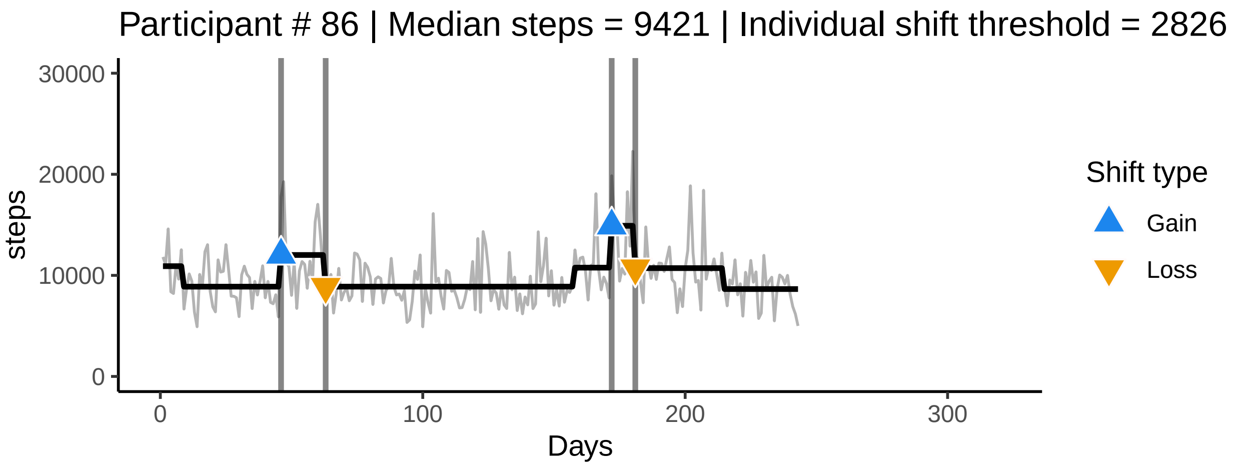

Participant # 86 | Median steps = 9421 | Individual shift threshold = 2826
30000
20000
10000
0
steps
0
100
200
300
Days
Shift type
Gain
Loss


```
##
## [[87]]
```

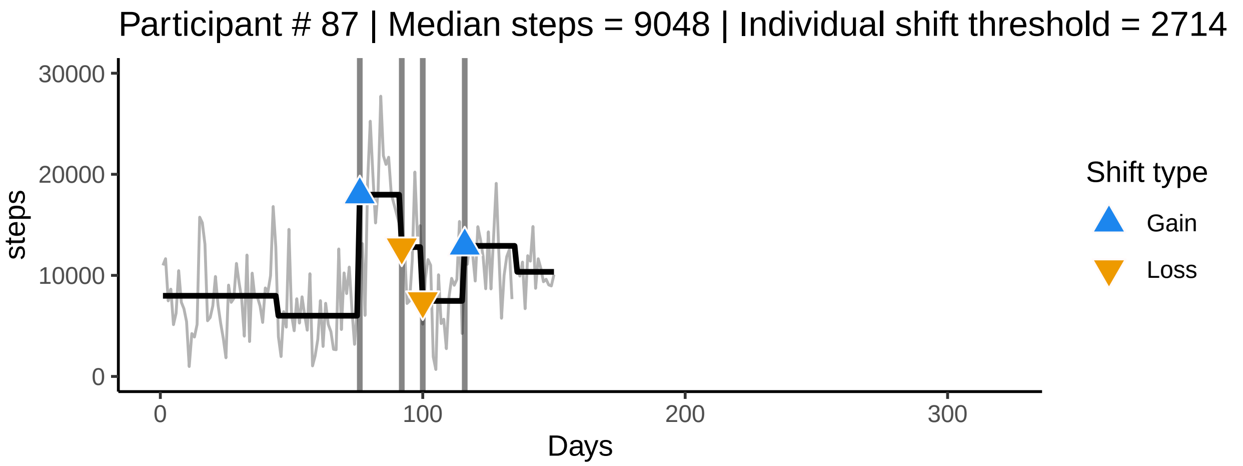

Participant # 87 | Median steps = 9048 | Individual shift threshold = 2714
30000
20000
10000
0
steps
0
100
200
300
Days
Shift type
Gain
Loss

```
##
## [[88]]
```

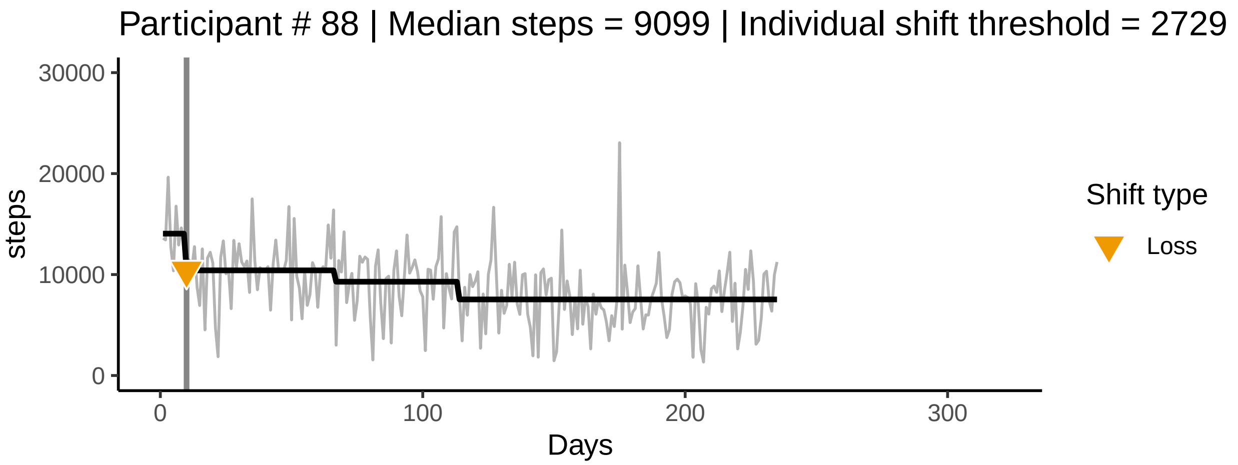
Participant # 88 | Median steps = 9099 | Individual shift threshold = 2729
steps
30000
20000
10000
0
0
100
200
300
Days
Shift type
Loss

```
##
## [[89]]
```

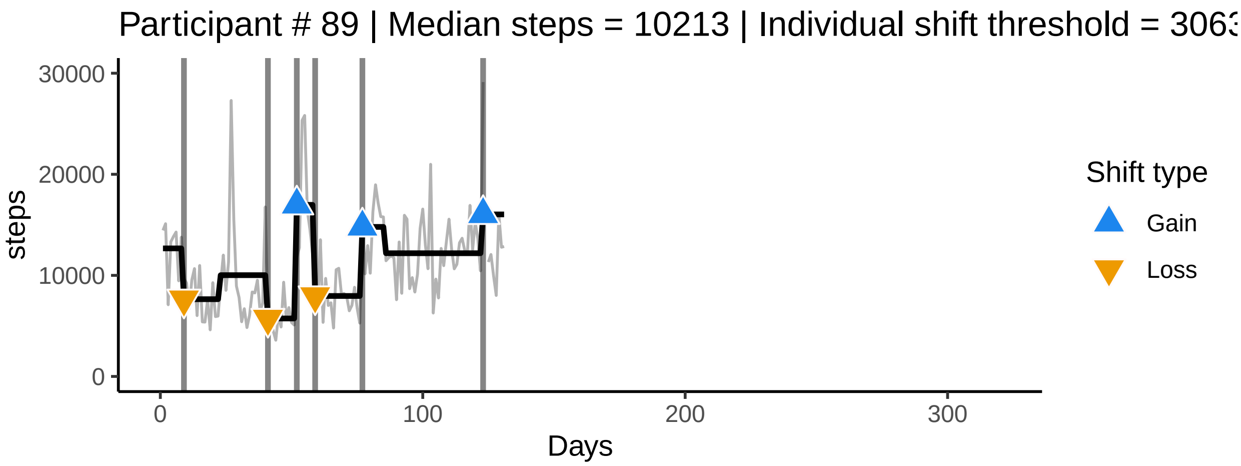
Participant # 89 | Median steps = 10213 | Individual shift threshold = 306
steps
30000
20000
10000
0
0
100
200
300
Days
Shift type
Gain
Loss

```
##
## [[90]]
```

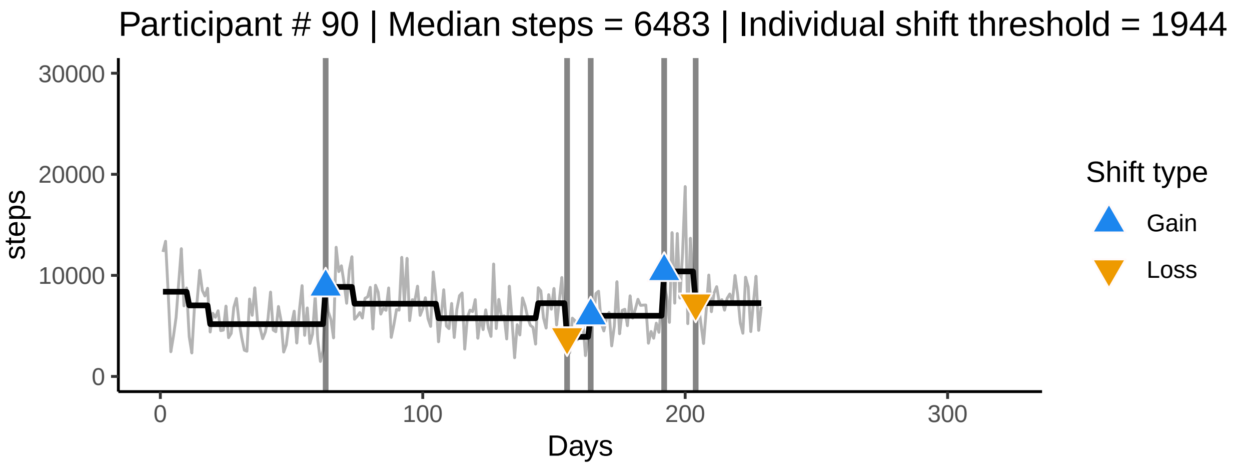
Participant # 90 | Median steps = 6483 | Individual shift threshold = 1944
steps
30000
20000
10000
0
0
100
200
300
Days
Shift type
Gain
Loss

```
##
## [[91]]
```

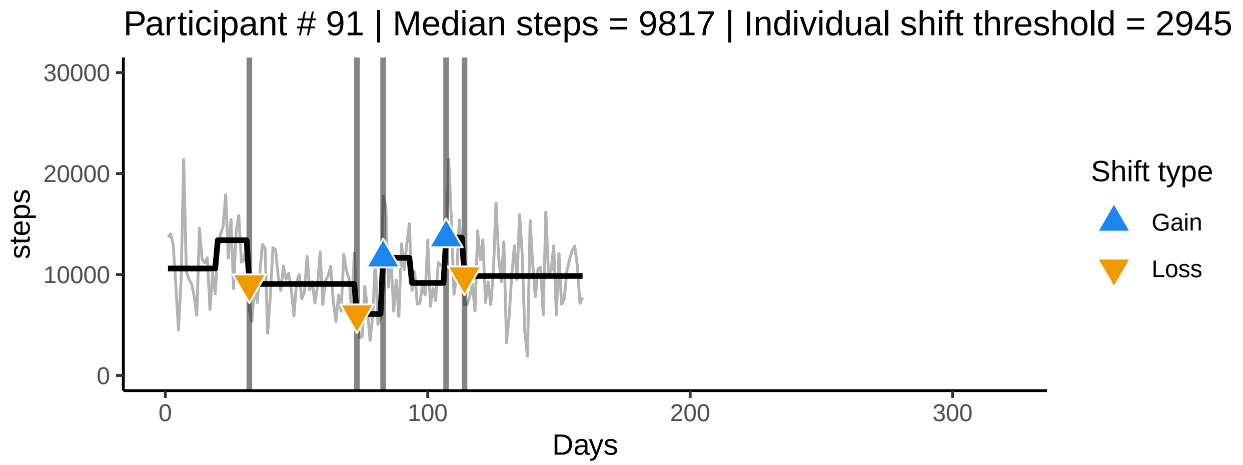

Participant # 91 | Median steps = 9817 | Individual shift threshold = 2945
30000
20000
10000
0
steps
0
100
200
300
Days
Shift type
Gain
Loss


```
##
## [[92]]
```

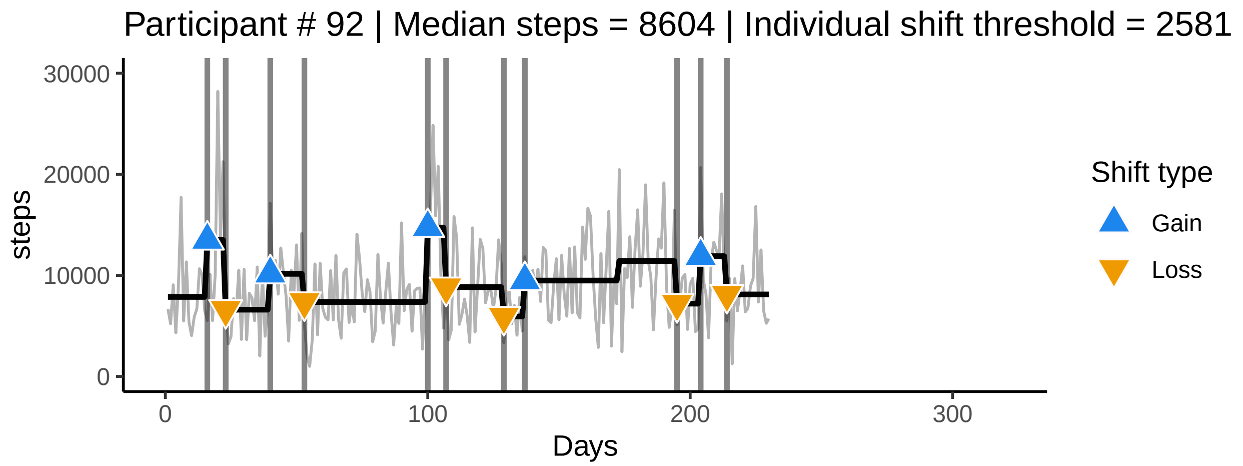

Participant # 92 | Median steps = 8604 | Individual shift threshold = 2581
30000
20000
10000
0
steps
0
100
200
300
Days
Shift type
Gain
Loss


```
##
## [[93]]
```

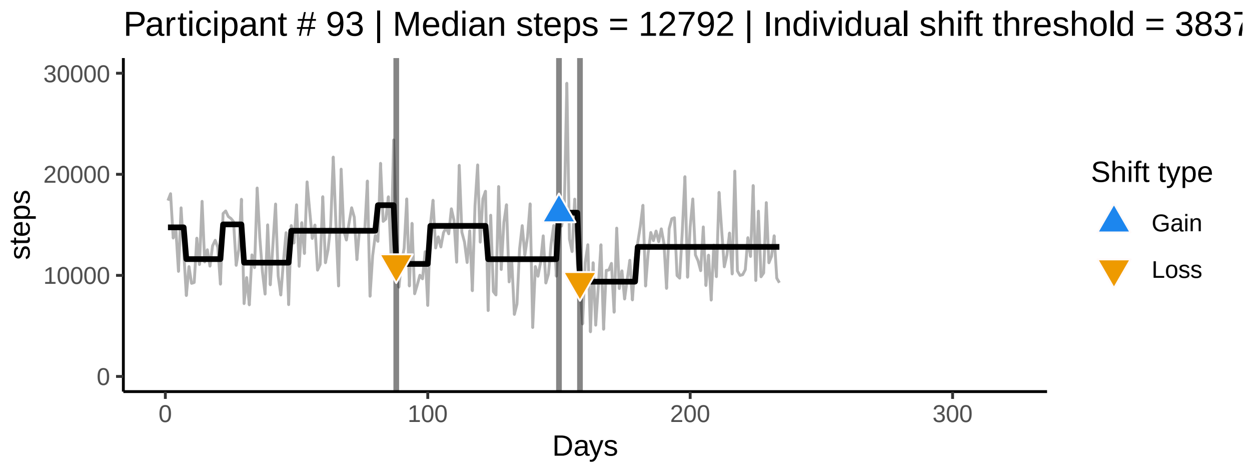

30000
20000
10000
0
steps
0
100
200
300
Days
Shift type
Gain
Loss

```
##
## [[94]]
```

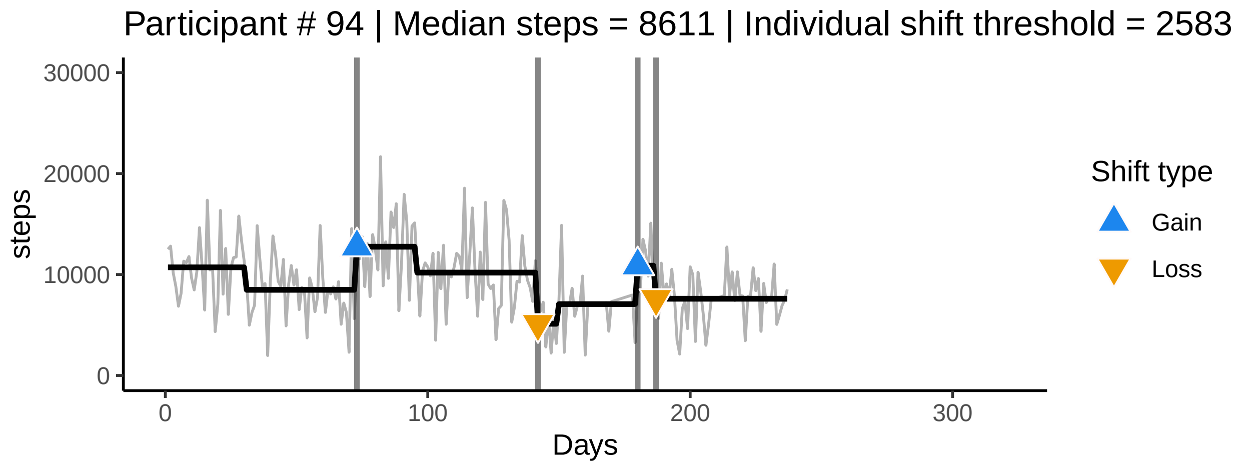

Participant # 94 | Median steps = 8611 | Individual shift threshold = 2583
30000
20000
10000
0
steps
0
100
200
300
Days
Shift type
Gain
Loss


```
##
## [[95]]
```

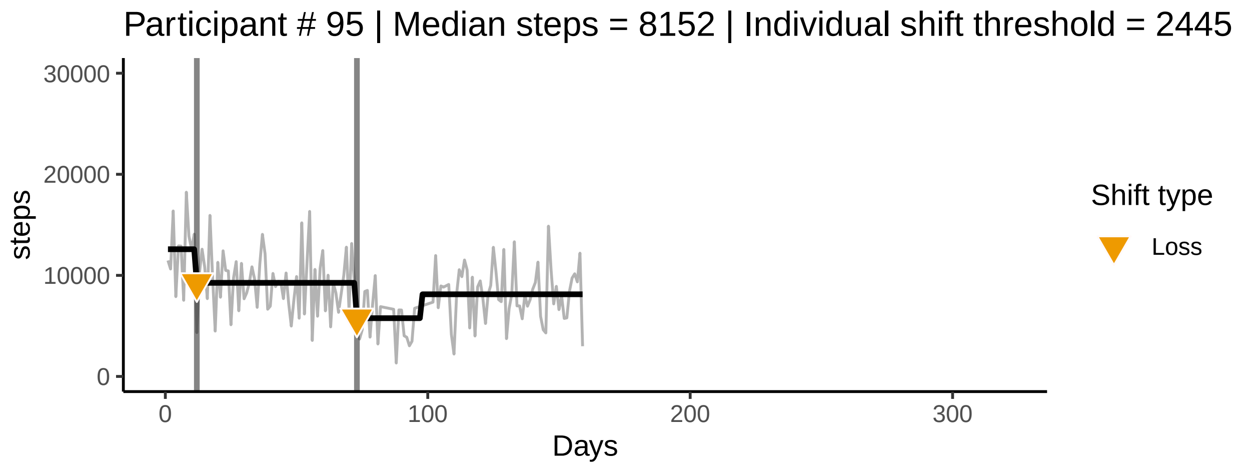

Participant # 95 | Median steps = 8152 | Individual shift threshold = 2445
30000
20000
10000
0
steps
0
100
200
300
Days
Shift type
Loss


```
##
## [[96]]
```

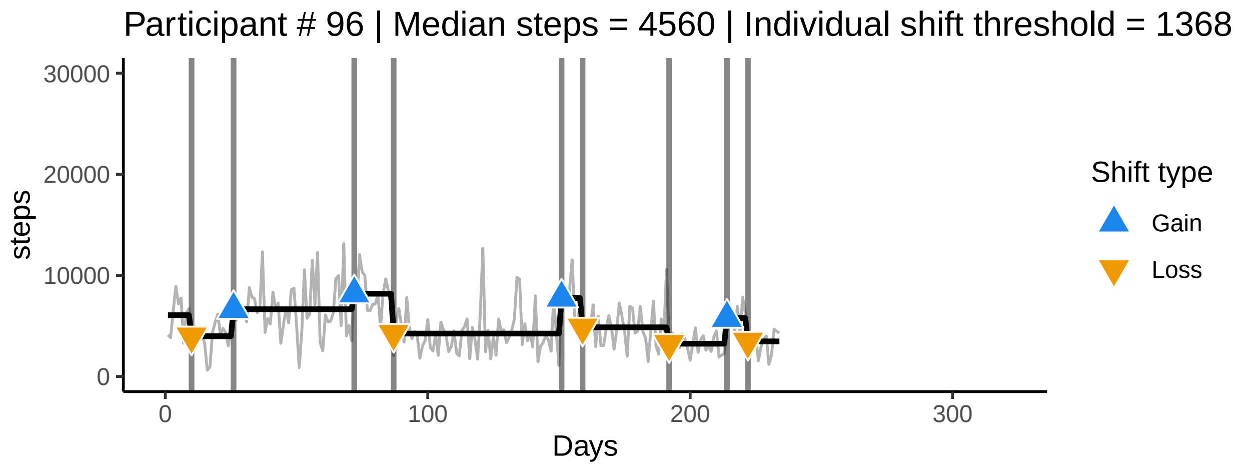

Participant # 96 | Median steps = 4560 | Individual shift threshold = 1368
30000
20000
10000
0
steps
0
100
200
300
Days
Shift type
Gain
Loss

```
##
## [[97]]
```

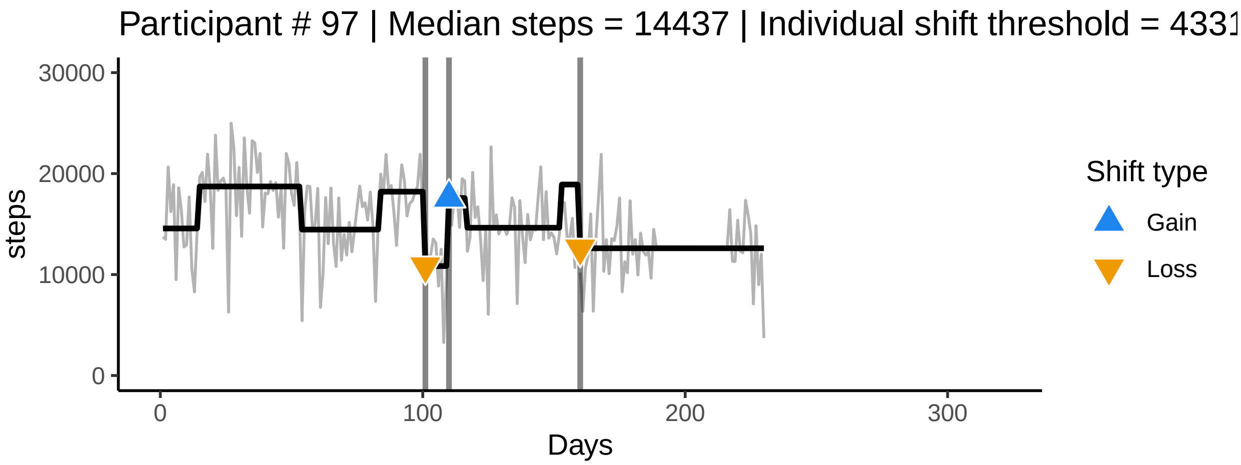
Participant # 97 | Median steps = 14437 | Individual shift threshold = 4331
steps
30000
20000
10000
0
0
100
200
300
Days
Shift type
Gain
Loss

```
##
## [[98]]
```

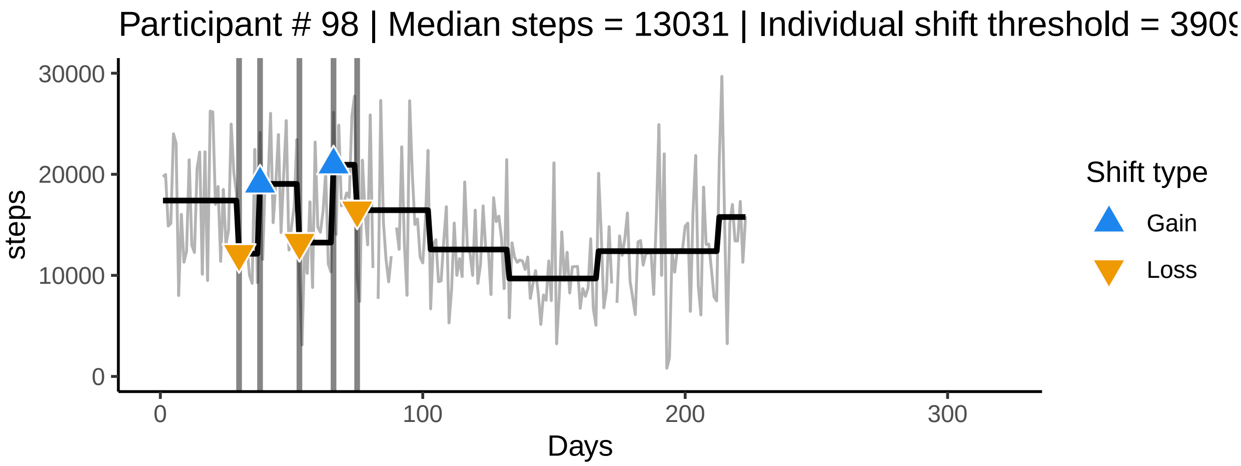
Participant # 98 | Median steps = 13031 | Individual shift threshold = 390
steps
30000
20000
10000
0
0
100
200
300
Days
Shift type
Gain
Loss

```
##
## [[99]]
```

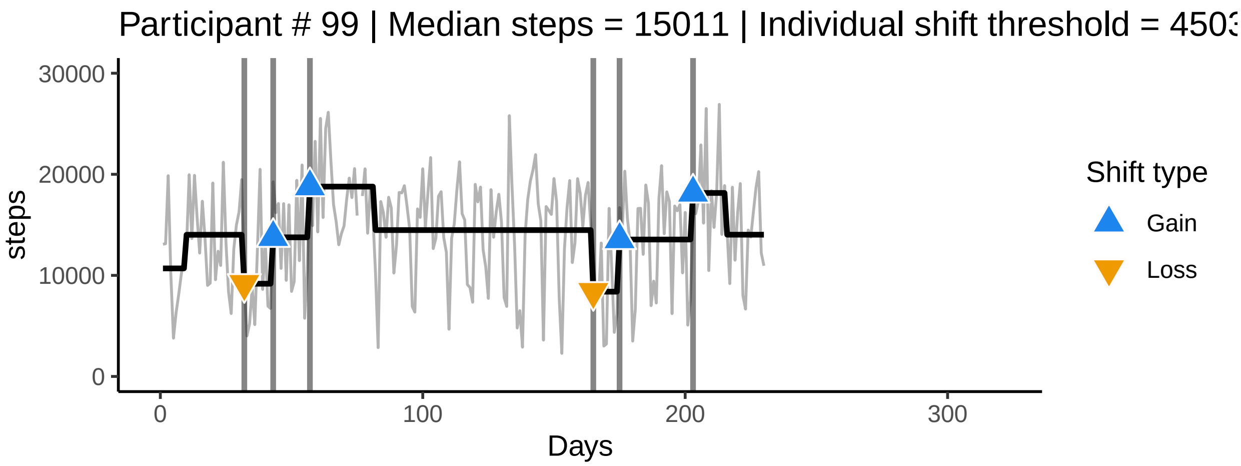
Participant # 99 | Median steps = 15011 | Individual shift threshold = 450
steps
30000
20000
10000
0
0
100
200
300
Days
Shift type
Gain
Loss

```
##
## [[100]]
```

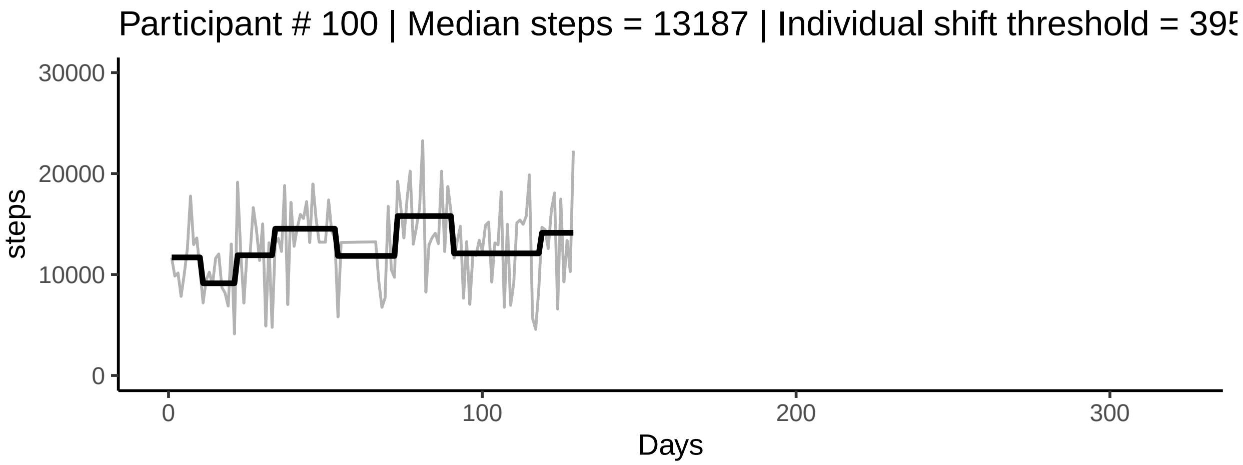

Participant # 100 | Median steps = 13187 | Individual shift threshold = 395
steps
30000
20000
10000
0
0
100
200
300
Days


```
##
## [[101]]
```

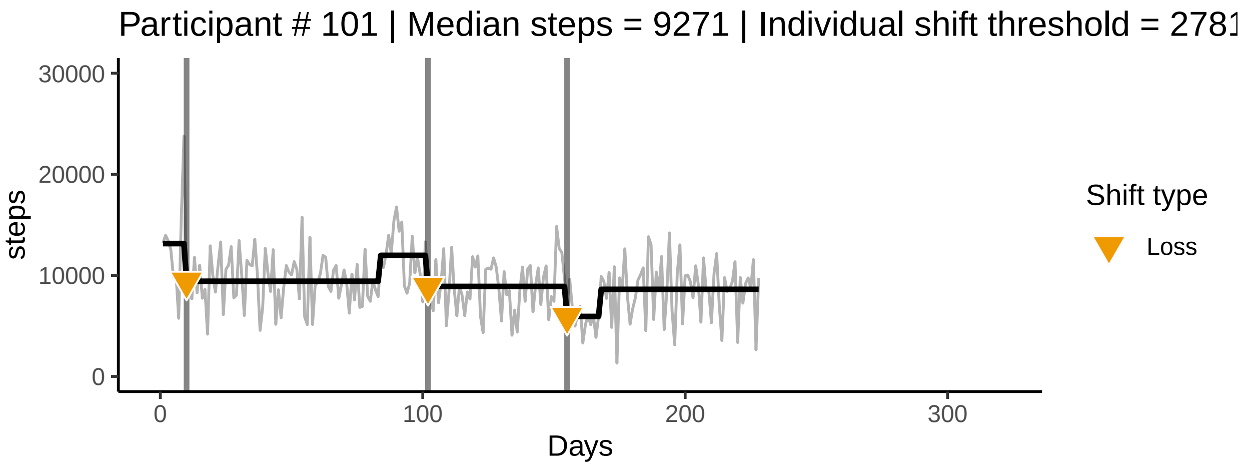

Participant # 101 | Median steps = 9271 | Individual shift threshold = 2781
steps
30000
20000
10000
0
0
100
200
300
Days
Shift type
Loss


```
##
## [[102]]
```

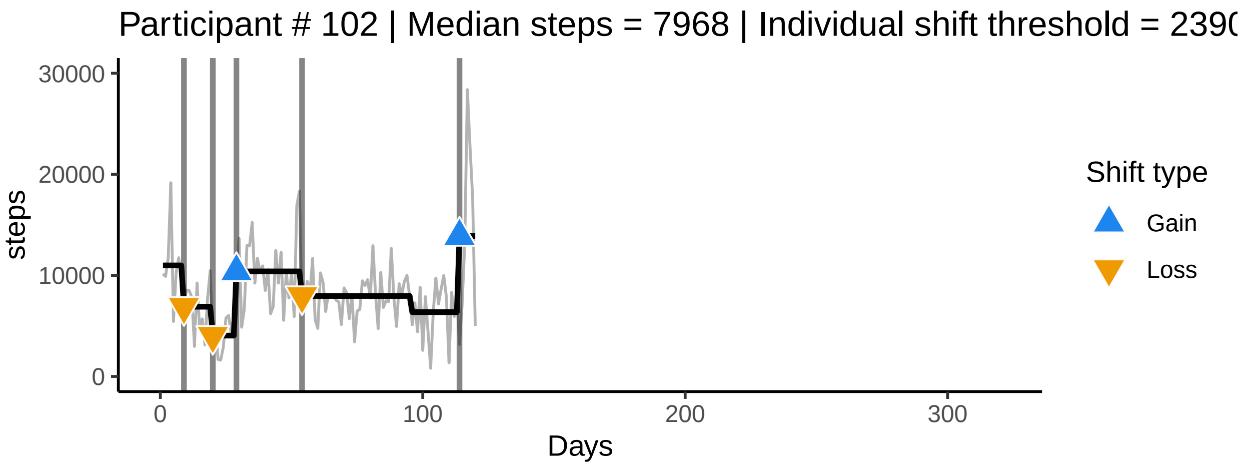

Participant # 102 | Median steps = 7968 | Individual shift threshold = 239
steps
30000
20000
10000
0
0
100
200
300
Days
Shift type
Gain
Loss

```
##
## [[103]]
```

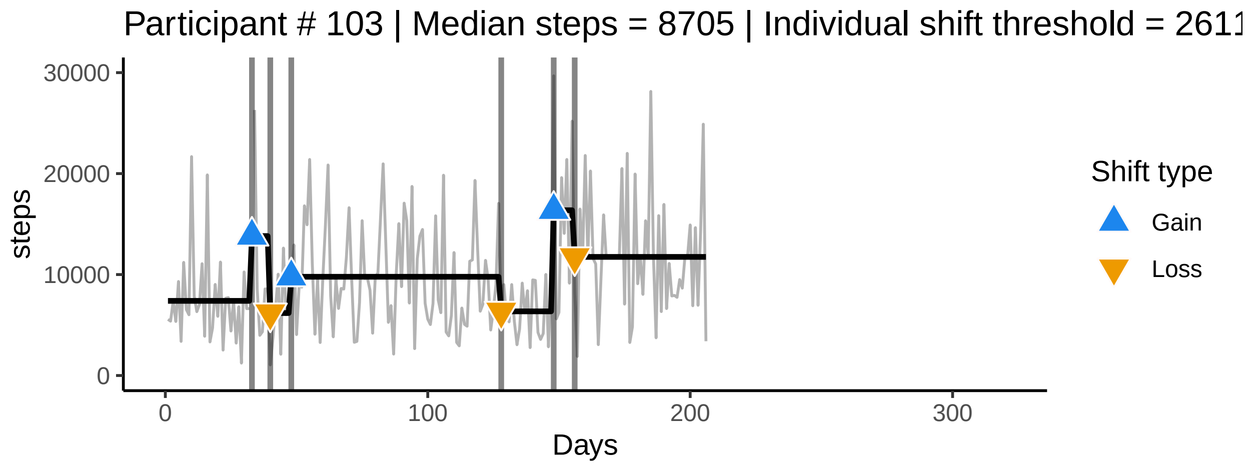

Participant # 103 | Median steps = 8705 | Individual shift threshold = 2611
steps
30000
20000
10000
0
0
100
200
300
Days
Shift type
Gain
Loss


```
##
## [[104]]
```

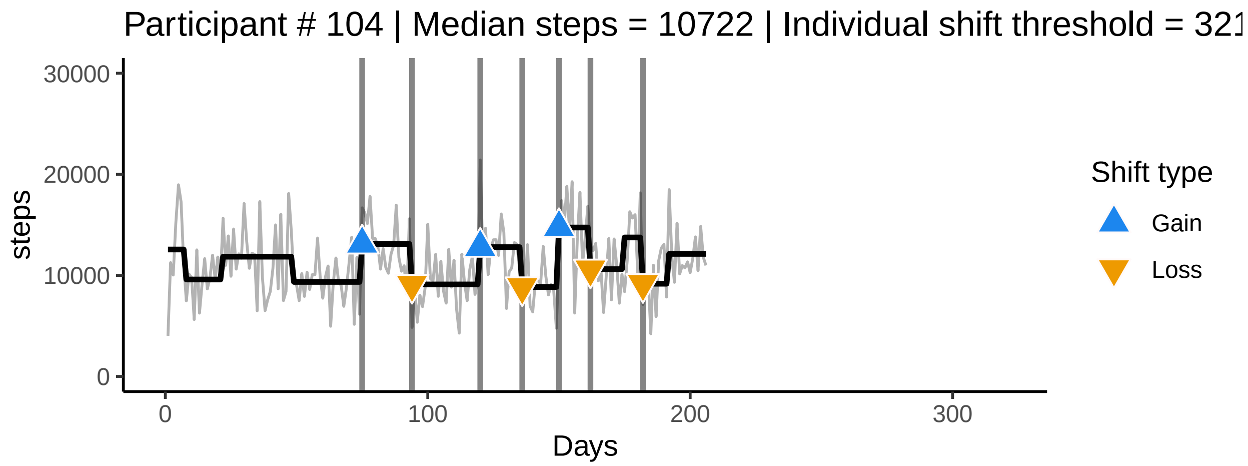

Participant # 104 | Median steps = 10722 | Individual shift threshold = 321
steps
30000
20000
10000
0
0
100
200
300
Days
Shift type
Gain
Loss


```
##
## [[105]]
```

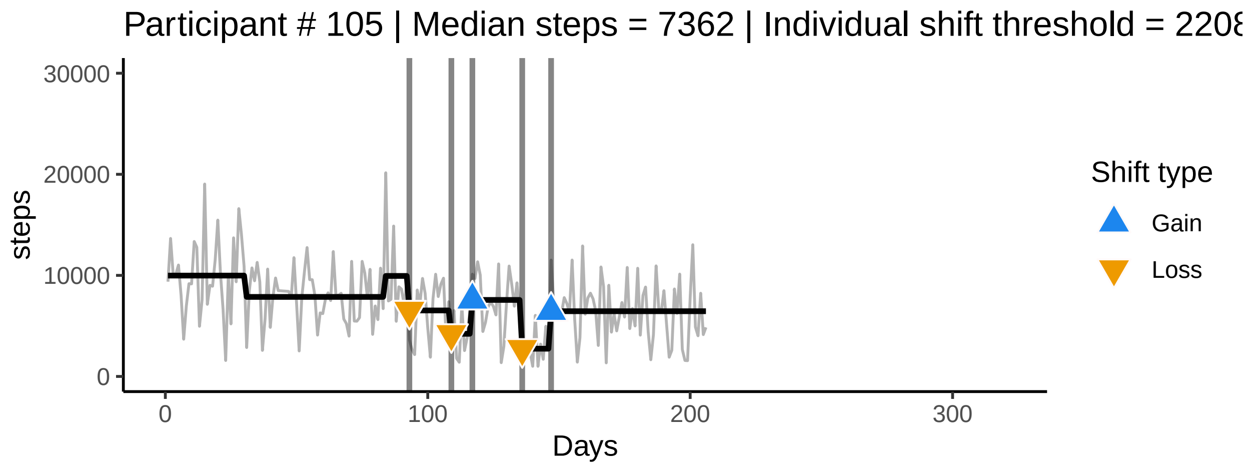

Participant # 105 | Median steps = 7362 | Individual shift threshold = 220
steps
30000
20000
10000
0
0
100
200
300
Days
Shift type
Gain
Loss

```
##
## [[106]]
```

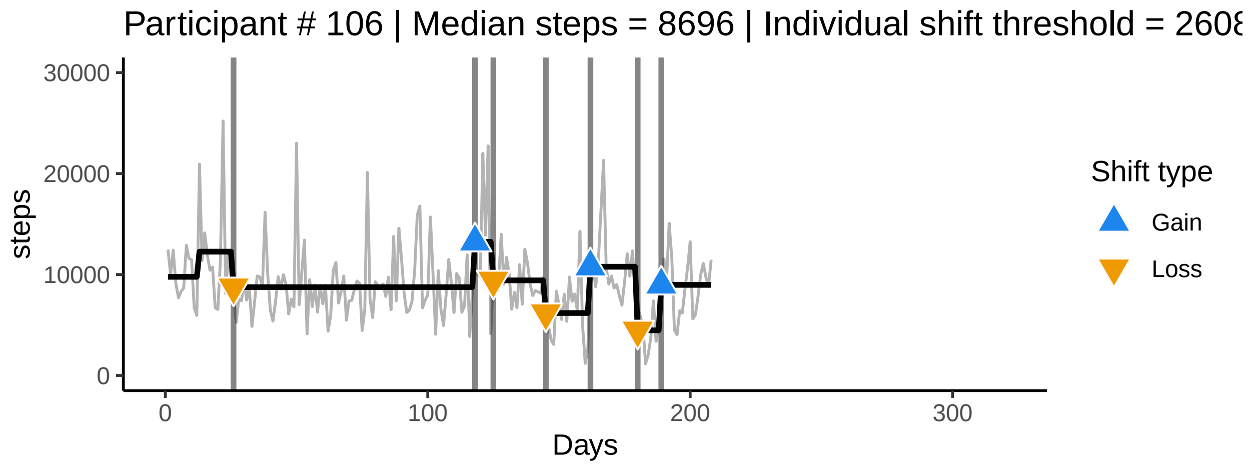
Participant # 106 | Median steps = 8696 | Individual shift threshold = 260
steps
30000
20000
10000
0
0
100
200
300
Days
Shift type
Gain
Loss

```
##
## [[107]]
```

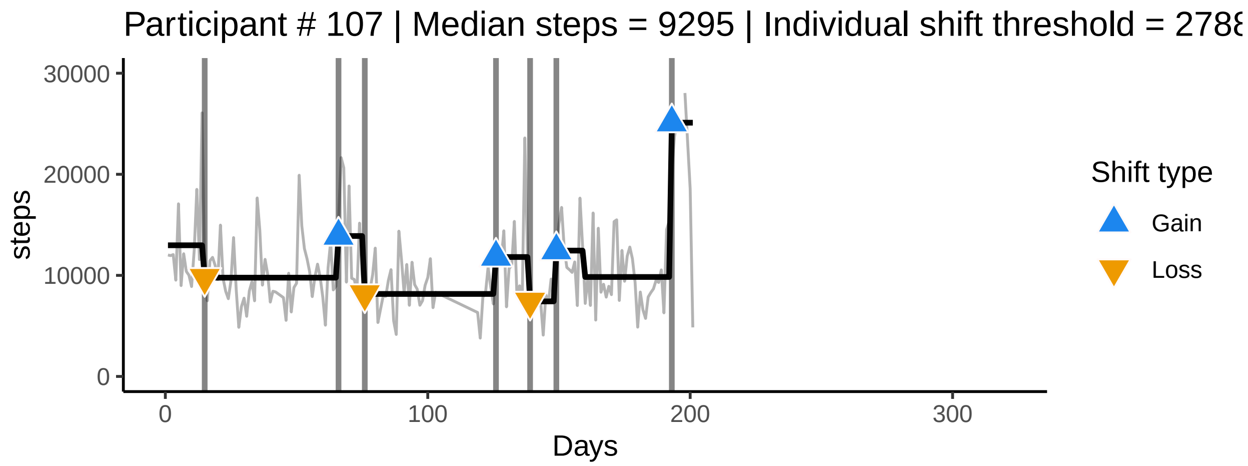
Participant # 107 | Median steps = 9295 | Individual shift threshold = 278
steps
30000
20000
10000
0
0
100
200
300
Days
Shift type
Gain
Loss

```
##
## [[108]]
```

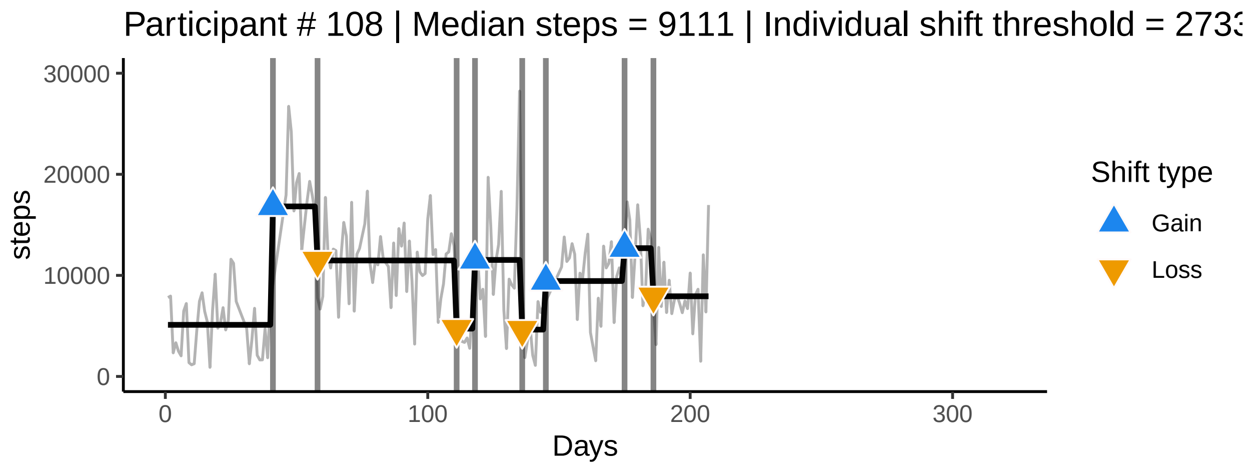
Participant # 108 | Median steps = 9111 | Individual shift threshold = 273
steps
30000
20000
10000
0
0
100
200
300
Days
Shift type
Gain
Loss

```
##
## [[109]]
```

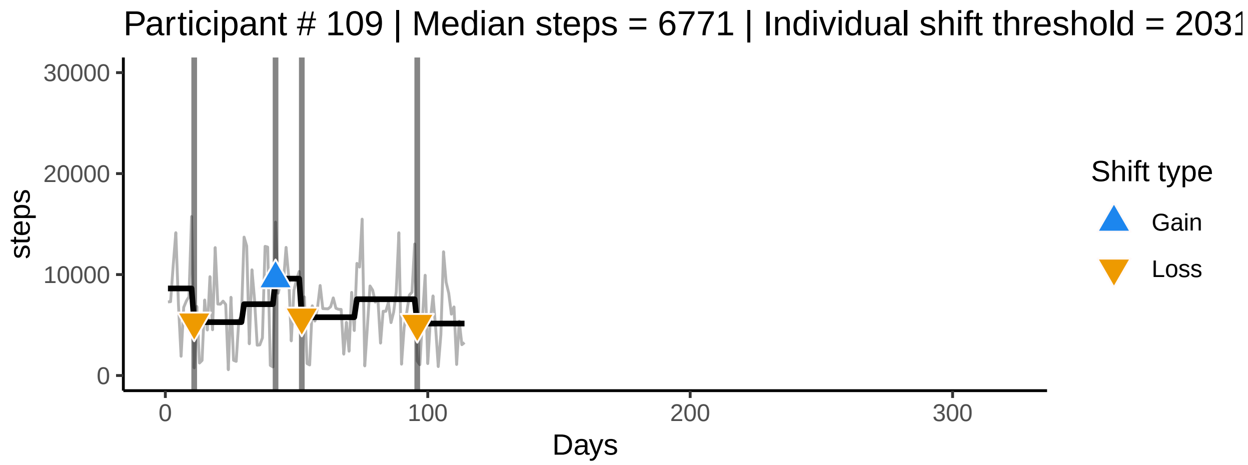
Participant # 109 | Median steps = 6771 | Individual shift threshold = 2031
30000
20000
10000
0
steps
0
100
200
300
Days
Shift type
Gain
Loss

```
##
## [[110]]
```

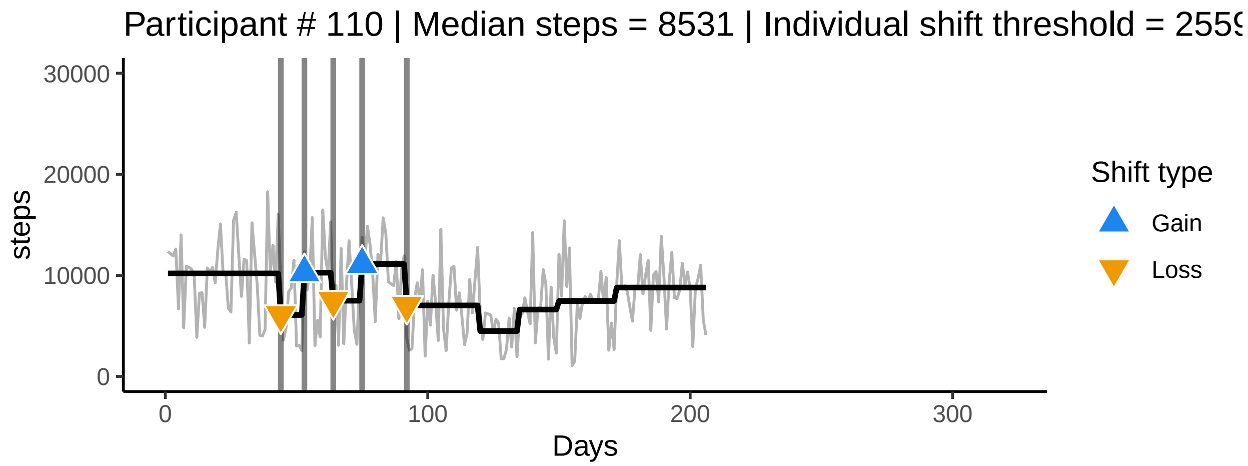
Participant # 110 | Median steps = 8531 | Individual shift threshold = 2559
30000
20000
10000
0
steps
0
100
200
300
Days
Shift type
Gain
Loss

```
##
## [[111]]
```

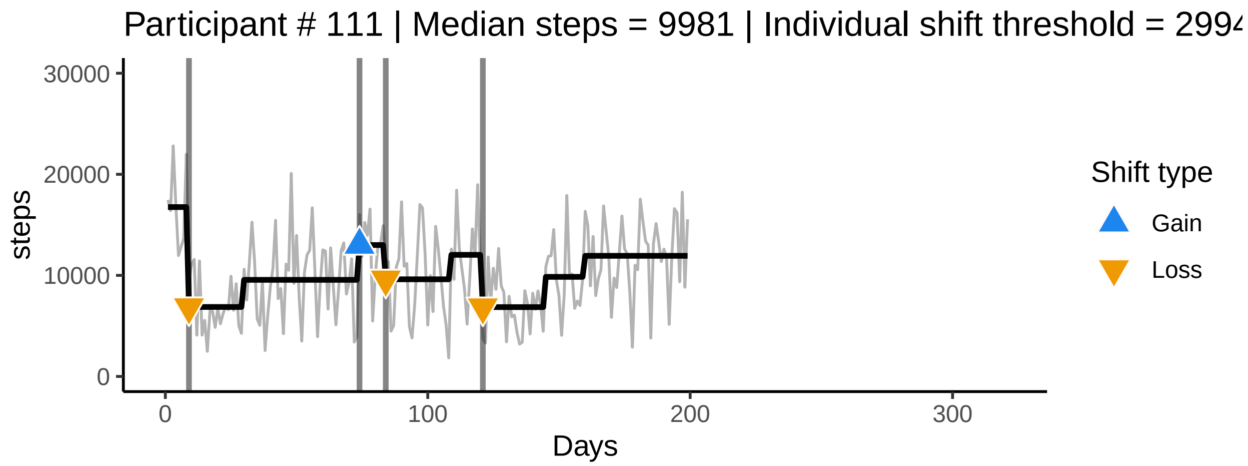
Participant # 111 | Median steps = 9981 | Individual shift threshold = 2994
30000
20000
10000
0
steps
0
100
200
300
Days
Shift type
Gain
Loss

```
##
## [[112]]
```

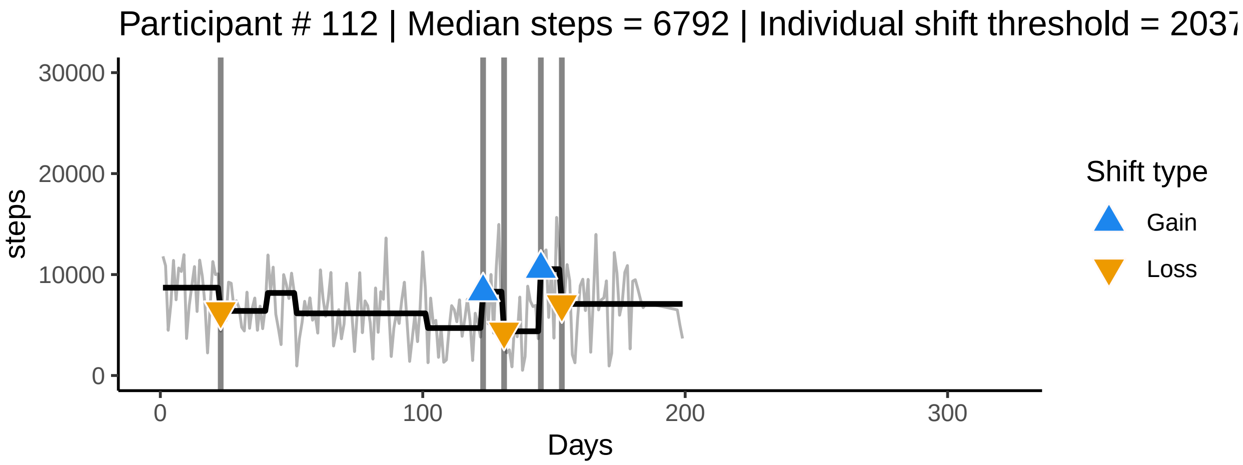

Participant # 112 | Median steps = 6792 | Individual shift threshold = 2037
steps
30000
20000
10000
0
0
100
200
300
Days
Shift type
Gain
Loss


```
##
## [[113]]
```

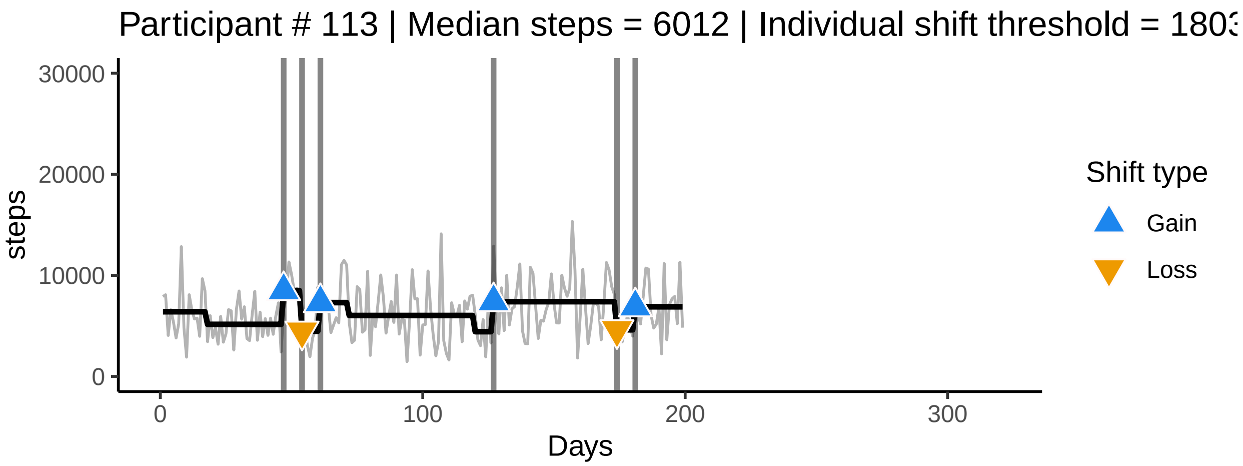

Participant # 113 | Median steps = 6012 | Individual shift threshold = 1803
steps
30000
20000
10000
0
0
100
200
300
Days
Shift type
Gain
Loss


```
##
## [[114]]
```

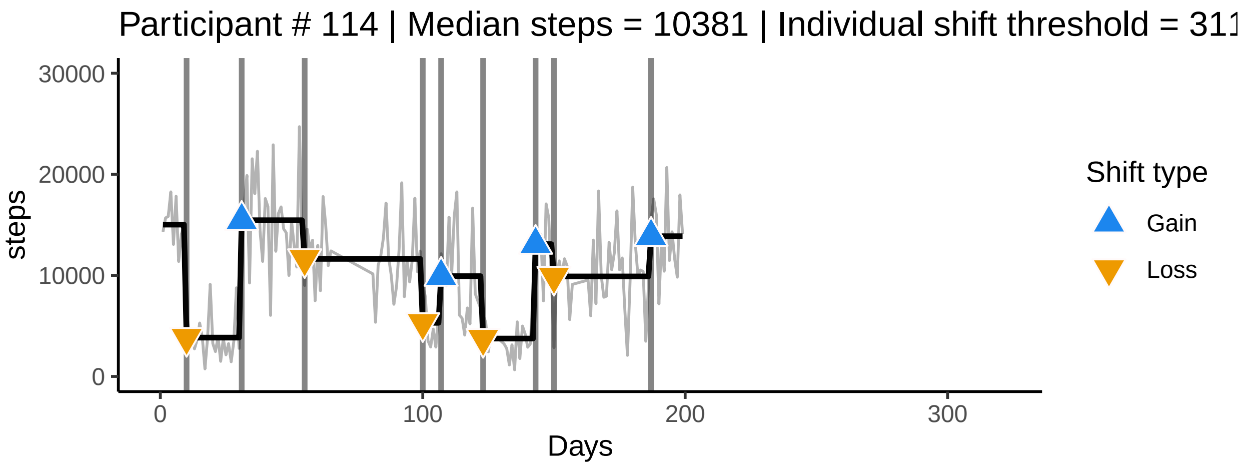

Participant # 114 | Median steps = 10381 | Individual shift threshold = 311
steps
30000
20000
10000
0
0
100
200
300
Days
Shift type
Gain
Loss

```
##
## [[115]]
```

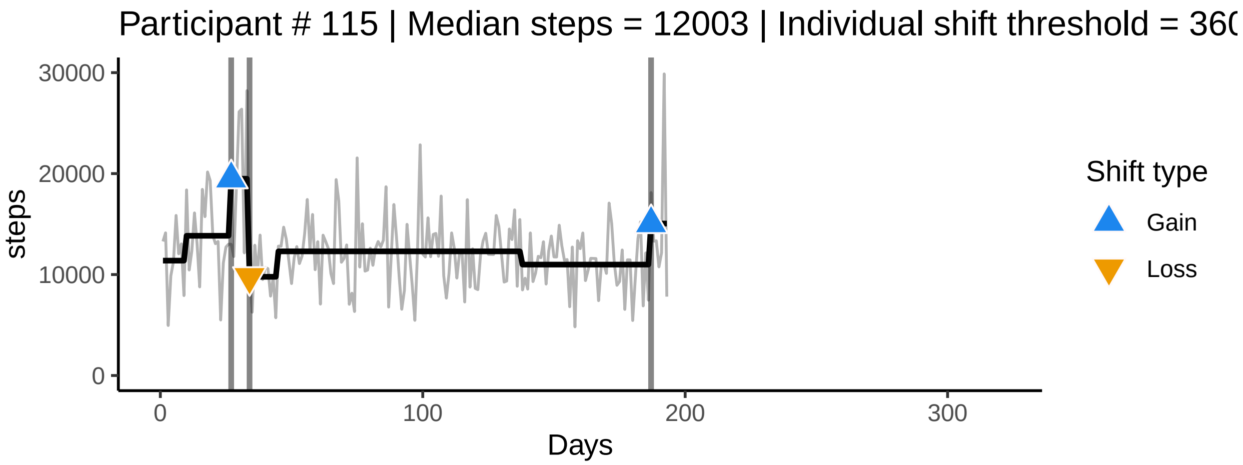
Participant # 115 | Median steps = 12003 | Individual shift threshold = 360
30000
20000
10000
0
steps
0
100
200
300
Days
Shift type
Gain
Loss

```
##
## [[116]]
```

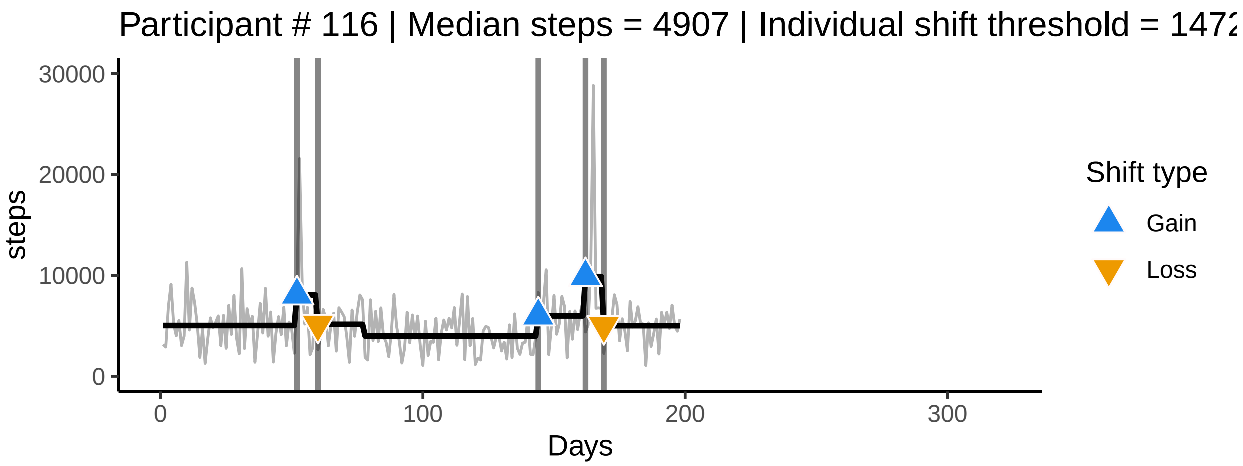
Participant # 116 | Median steps = 4907 | Individual shift threshold = 1472
30000
20000
10000
0
steps
0
100
200
300
Days
Shift type
Gain
Loss

```
##
## [[117]]
```

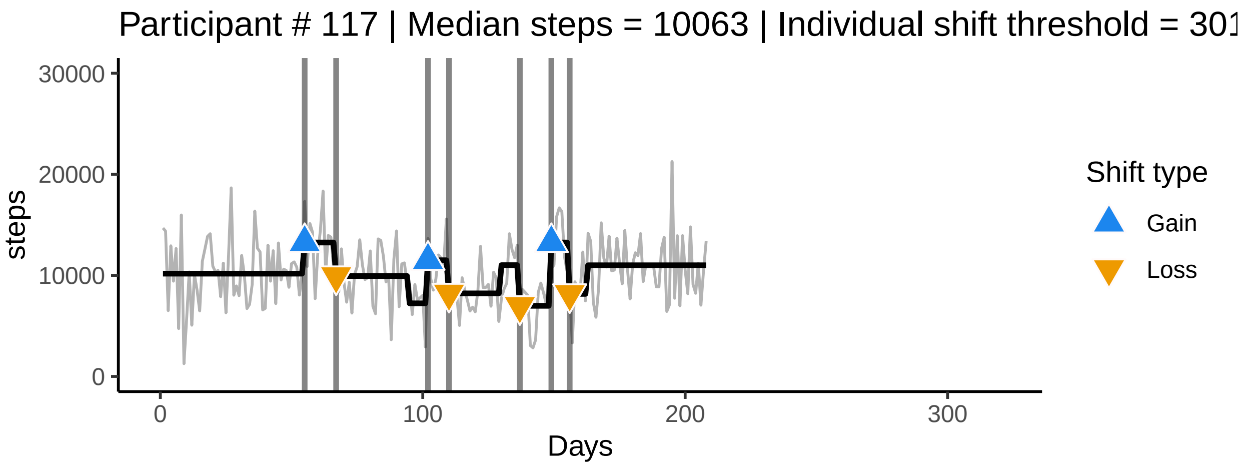
Participant # 117 | Median steps = 10063 | Individual shift threshold = 301
30000
20000
10000
0
steps
0
100
200
300
Days
Shift type
Gain
Loss

```
##
## [[118]]
```

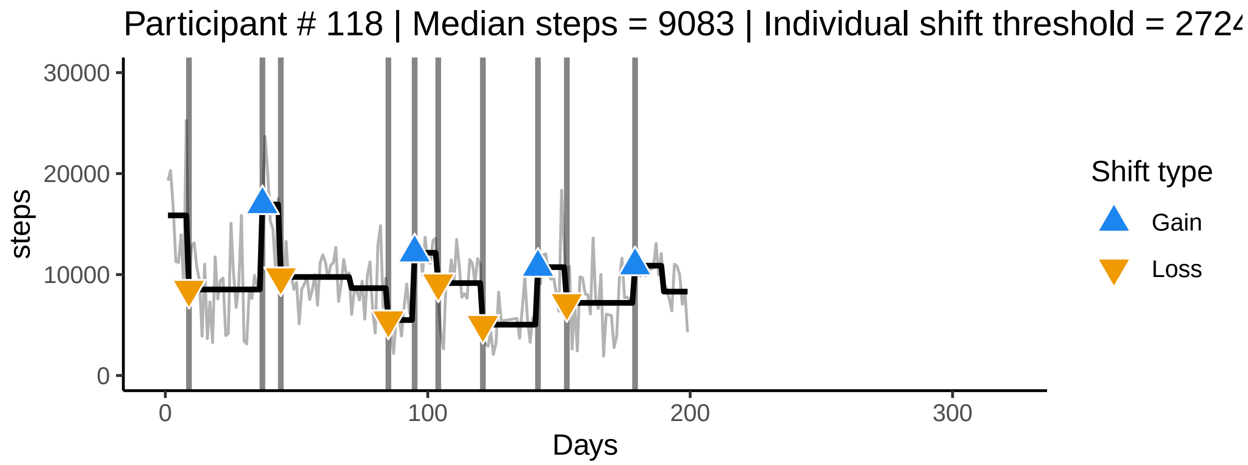
Participant # 118 | Median steps = 9083 | Individual shift threshold = 272
steps
30000
20000
10000
0
0
100
200
300
Days
Shift type
Gain
Loss

```
##
## [[119]]
```

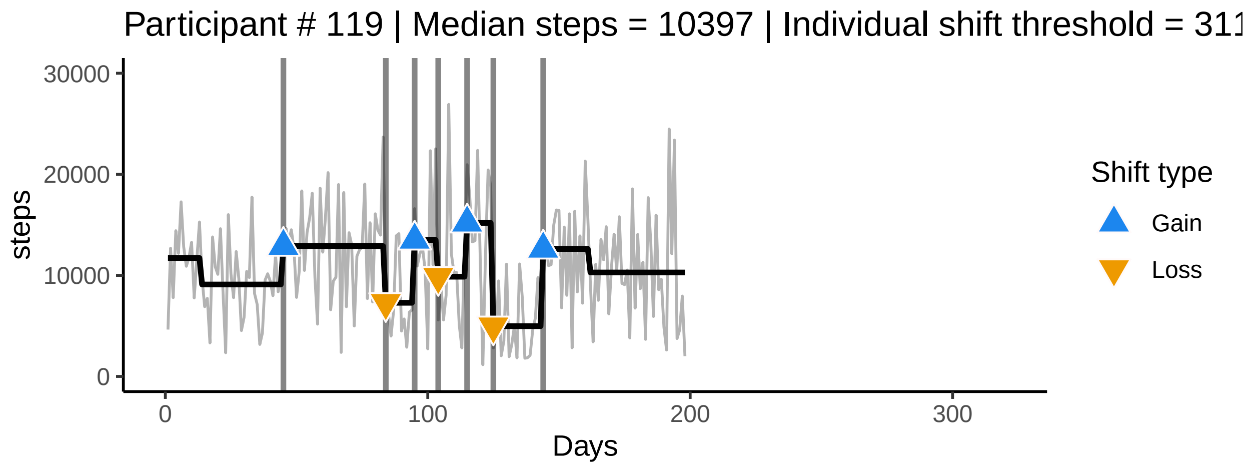
Participant # 119 | Median steps = 10397 | Individual shift threshold = 311
steps
30000
20000
10000
0
0
100
200
300
Days
Shift type
Gain
Loss

```
##
## [[120]]
```

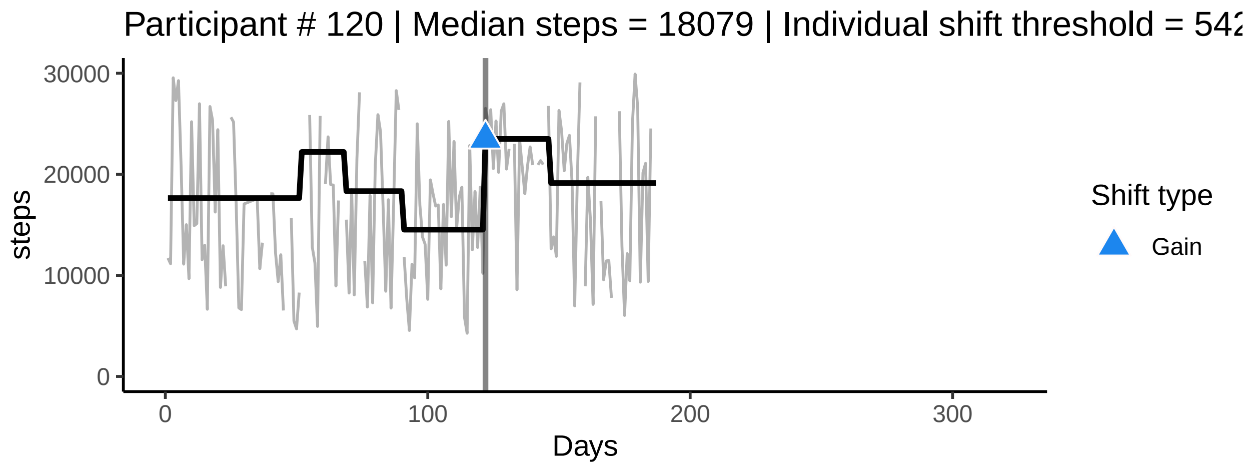
Participant # 120 | Median steps = 18079 | Individual shift threshold = 54
steps
30000
20000
10000
0
0
100
200
300
Days
Shift type
Gain

```
##
## [[121]]
```

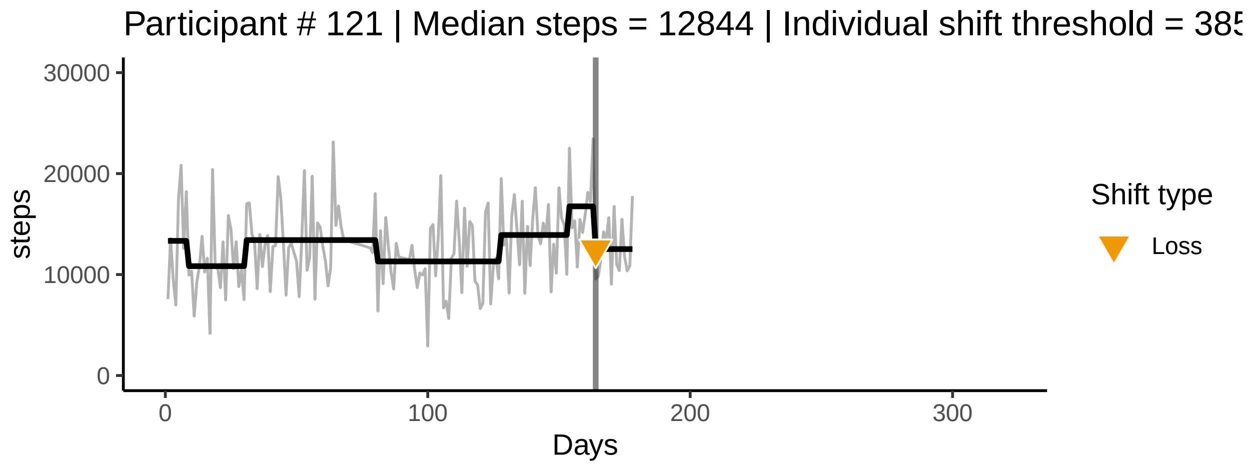

Participant # 121 | Median steps = 12844 | Individual shift threshold = 385
30000
20000
10000
0
steps
0
100
200
300
Days
Shift type
Loss


```
##
## [[122]]
```

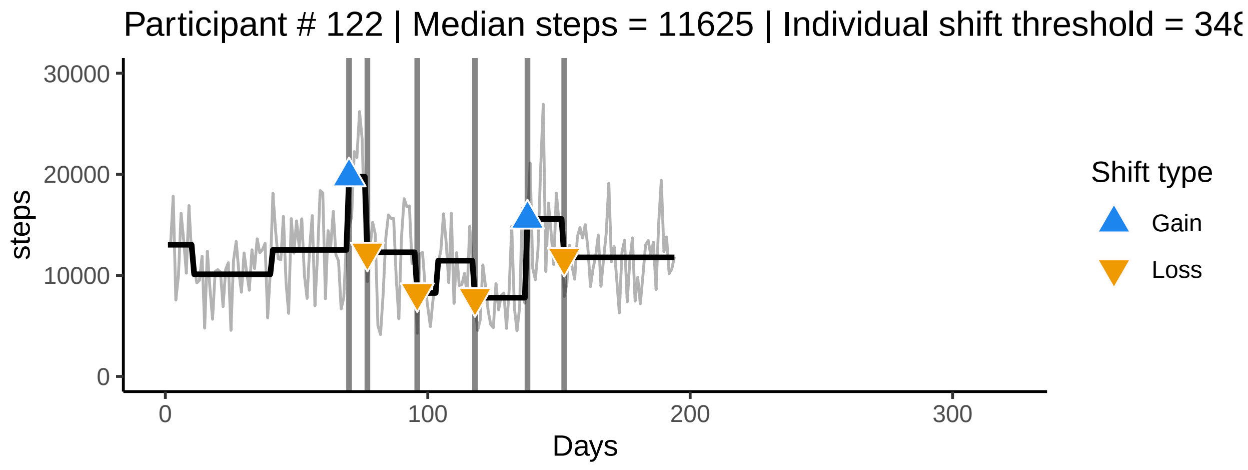

Participant # 122 | Median steps = 11625 | Individual shift threshold = 348
30000
20000
10000
0
steps
0
100
200
300
Days
Shift type
Gain
Loss


```
##
## [[123]]
```

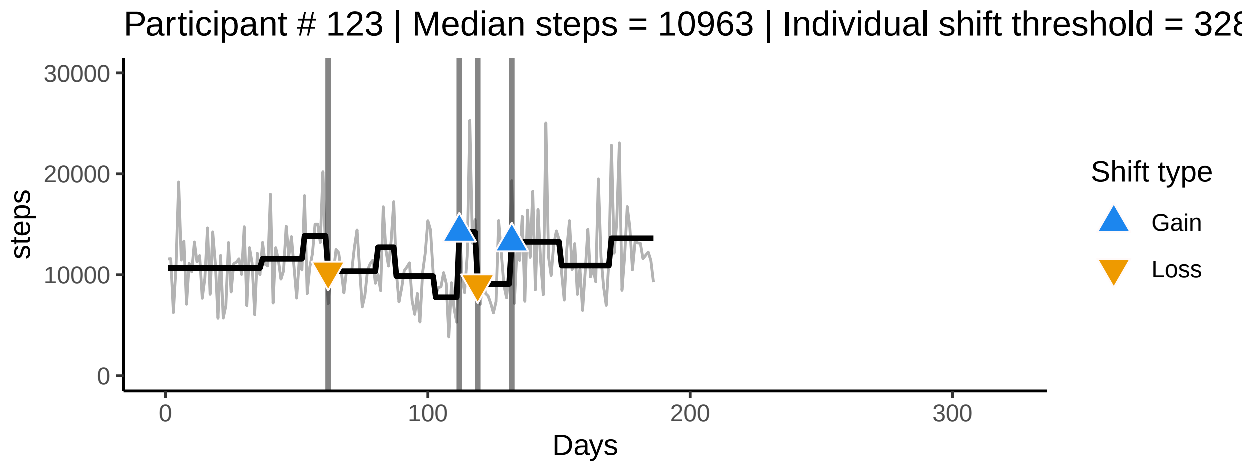

Participant # 123 | Median steps = 10963 | Individual shift threshold = 328
30000
20000
10000
0
steps
0
100
200
300
Days
Shift type
Gain
Loss

##
[[124]]

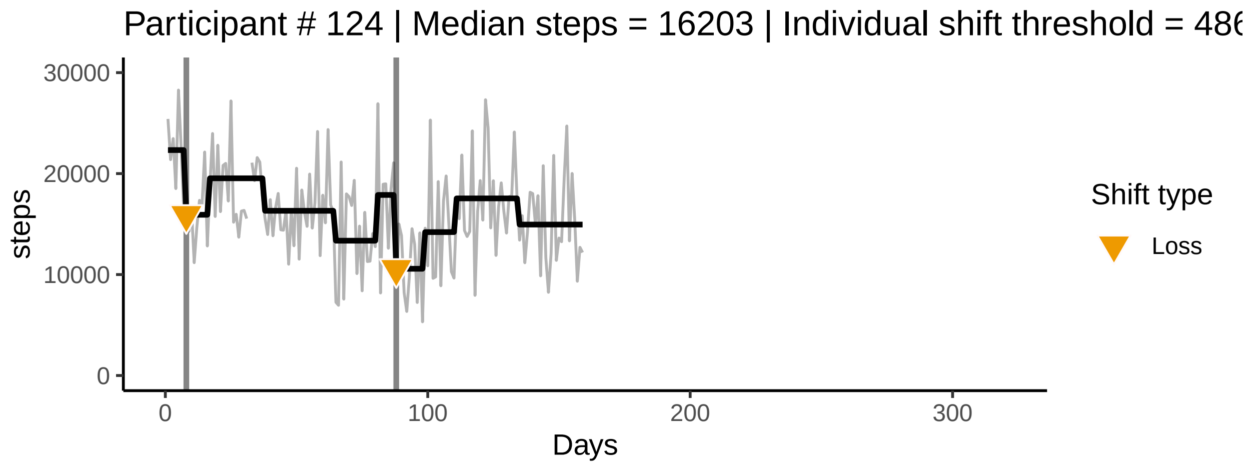
Participant # 124 | Median steps = 16203 | Individual shift threshold = 48[illegible]
30000
20000
10000
0
steps
0
100
200
300
Days
Shift type
Loss

##
[[125]]

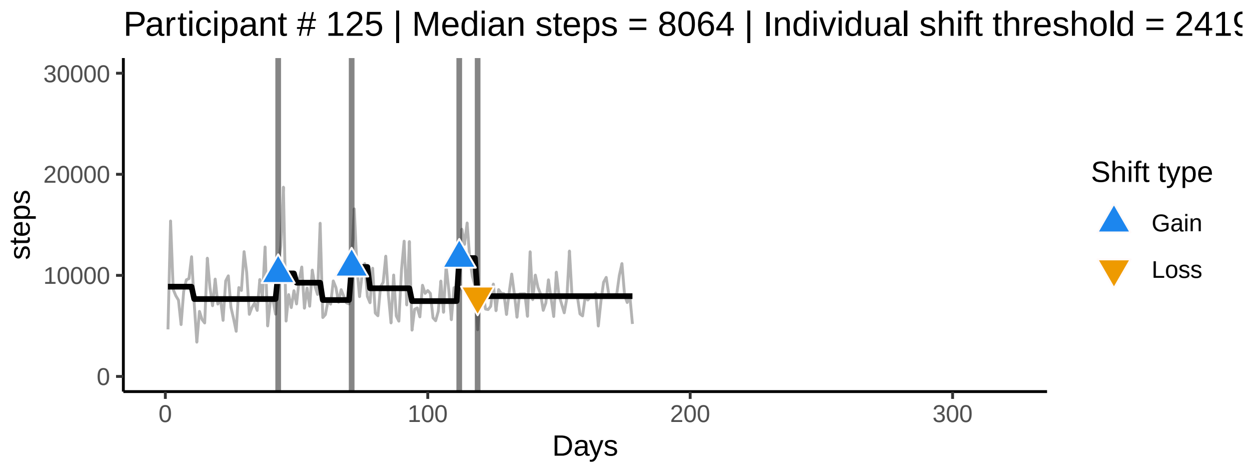
Participant # 125 | Median steps = 8064 | Individual shift threshold = 241[illegible]
30000
20000
10000
0
steps
0
100
200
300
Days
Shift type
Gain
Loss

##
[[126]]

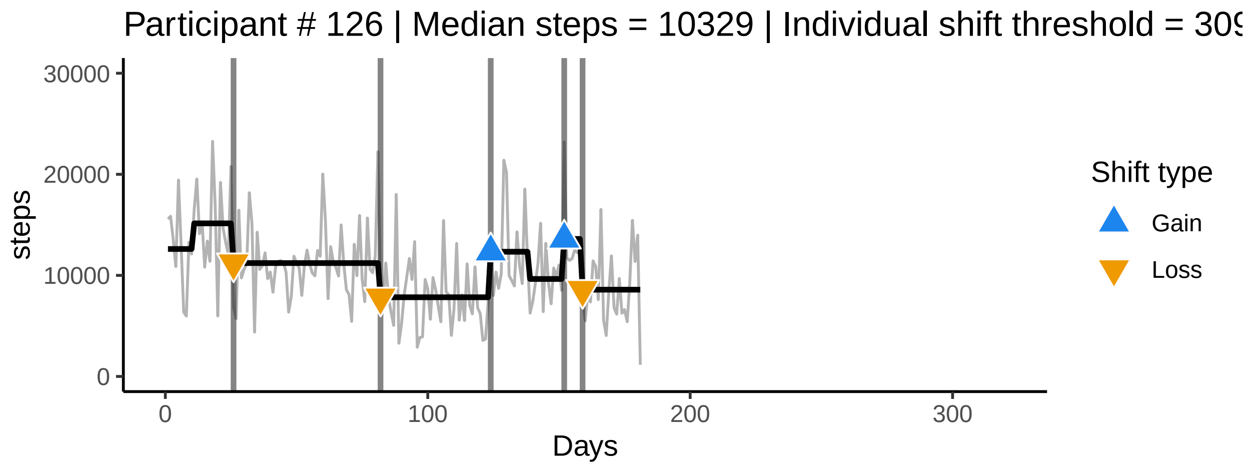
Participant # 126 | Median steps = 10329 | Individual shift threshold = 30[illegible]
30000
20000
10000
0
steps
0
100
200
300
Days
Shift type
Gain
Loss

```
##
## [[127]]
```

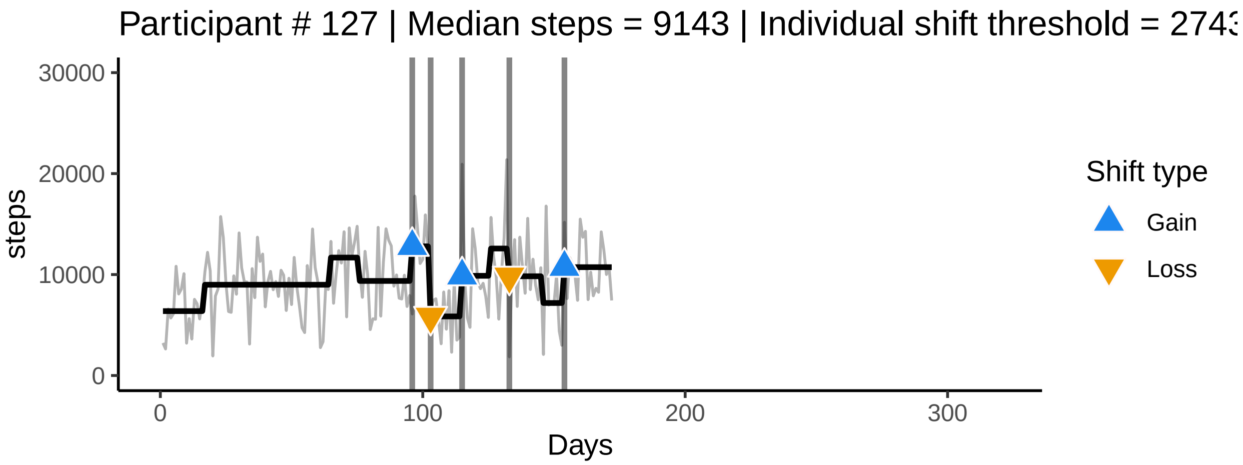

Participant # 127 | Median steps = 9143 | Individual shift threshold = 274
steps
30000
20000
10000
0
0
100
200
300
Days
Shift type
Gain
Loss


```
##
## [[128]]
```

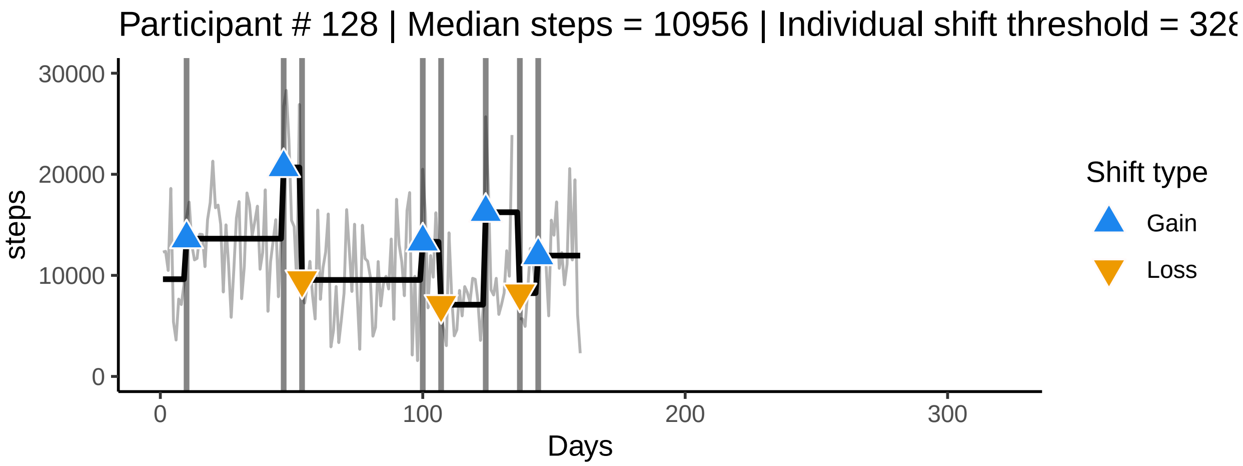

Participant # 128 | Median steps = 10956 | Individual shift threshold = 32
steps
30000
20000
10000
0
0
100
200
300
Days
Shift type
Gain
Loss


```
##
## [[129]]
```

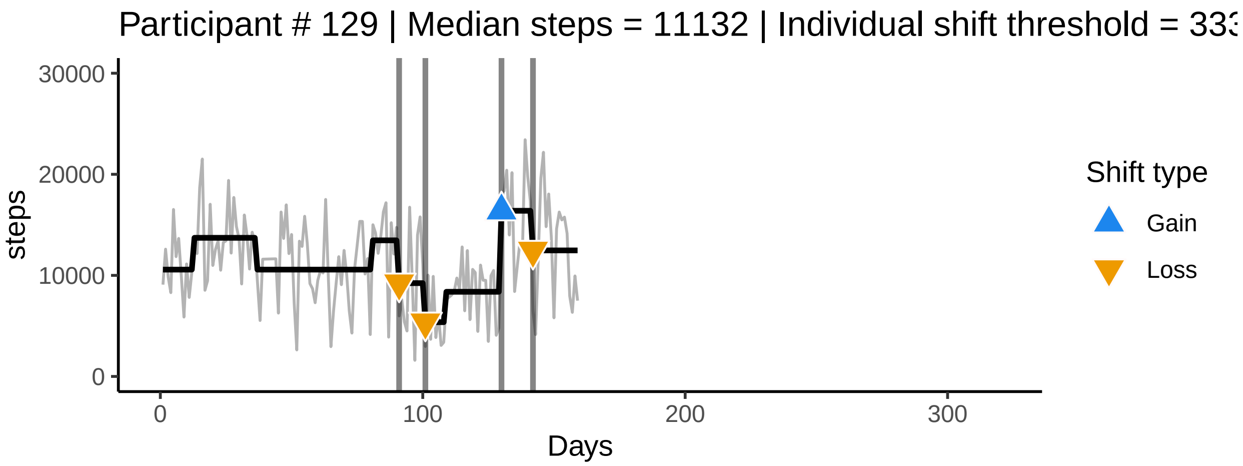

Participant # 129 | Median steps = 11132 | Individual shift threshold = 33
steps
30000
20000
10000
0
0
100
200
300
Days
Shift type
Gain
Loss

```
##
## [[130]]
```

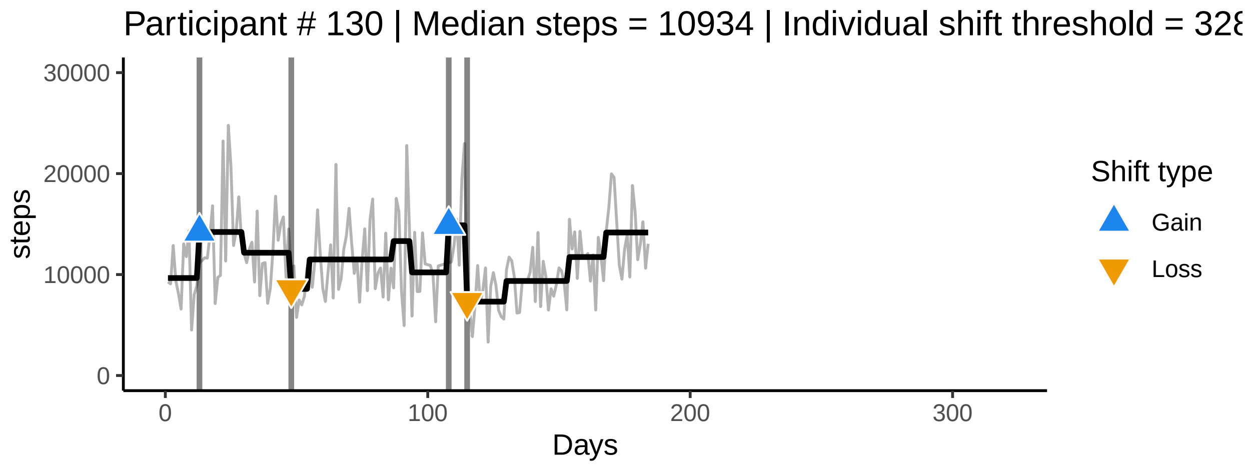

Participant # 130 | Median steps = 10934 | Individual shift threshold = 32
steps
30000
20000
10000
0
0
100
200
300
Days
Shift type
Gain
Loss


```
##
## [[131]]
```

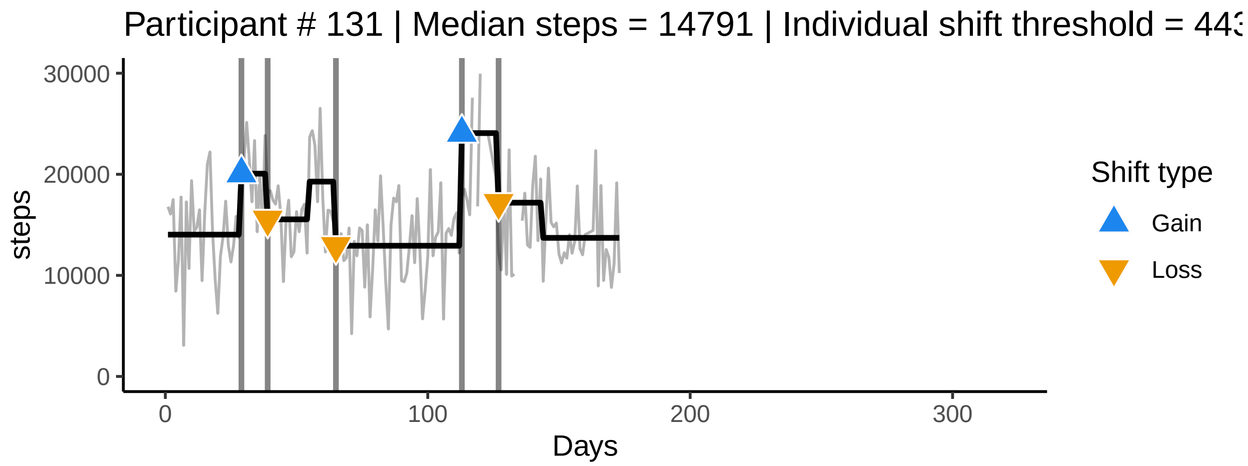

Participant # 131 | Median steps = 14791 | Individual shift threshold = 44
steps
30000
20000
10000
0
0
100
200
300
Days
Shift type
Gain
Loss


```
##
## [[132]]
```

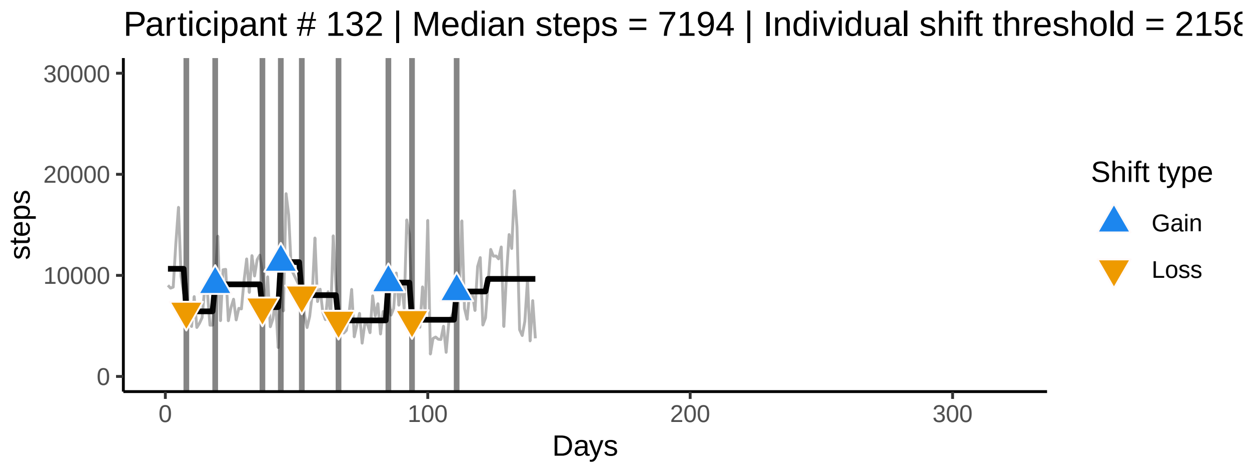

Participant # 132 | Median steps = 7194 | Individual shift threshold = 215
steps
30000
20000
10000
0
0
100
200
300
Days
Shift type
Gain
Loss

```
##
## [[133]]
```

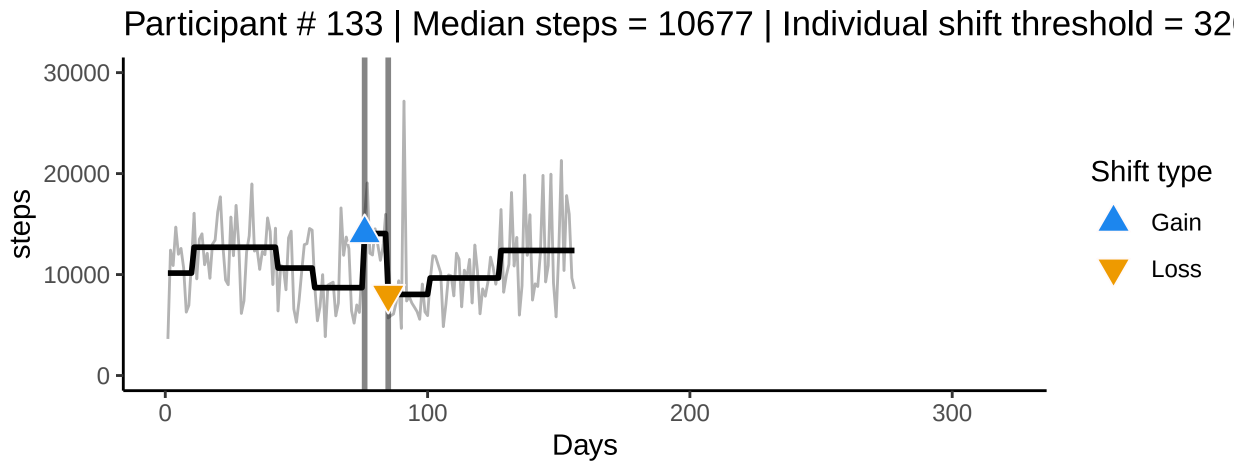
Participant # 133 | Median steps = 10677 | Individual shift threshold = 32(
steps
30000
20000
10000
0
0
100
200
300
Days
Shift type
Gain
Loss

```
##
## [[134]]
```

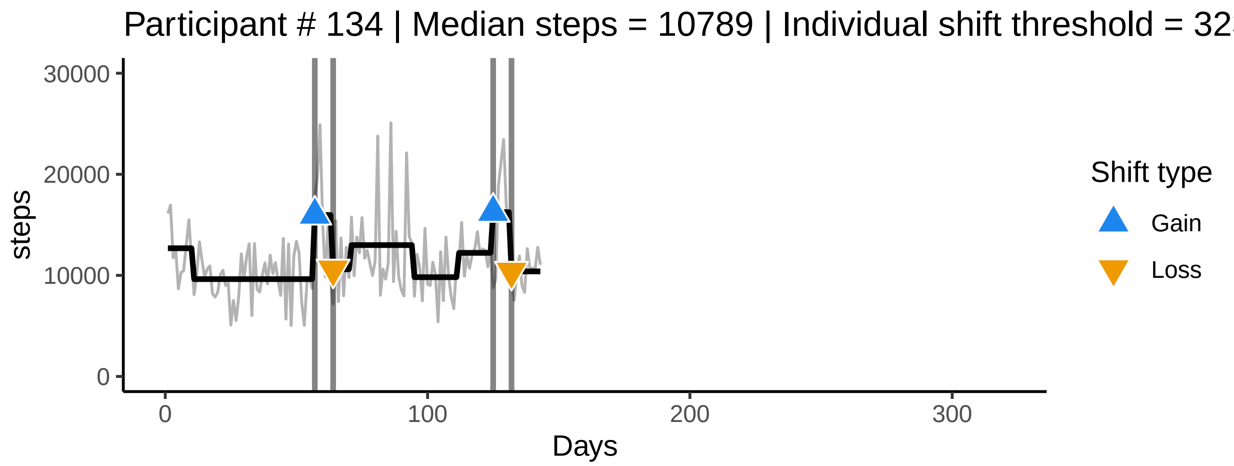
Participant # 134 | Median steps = 10789 | Individual shift threshold = 32:
steps
30000
20000
10000
0
0
100
200
300
Days
Shift type
Gain
Loss

```
##
## [[135]]
```

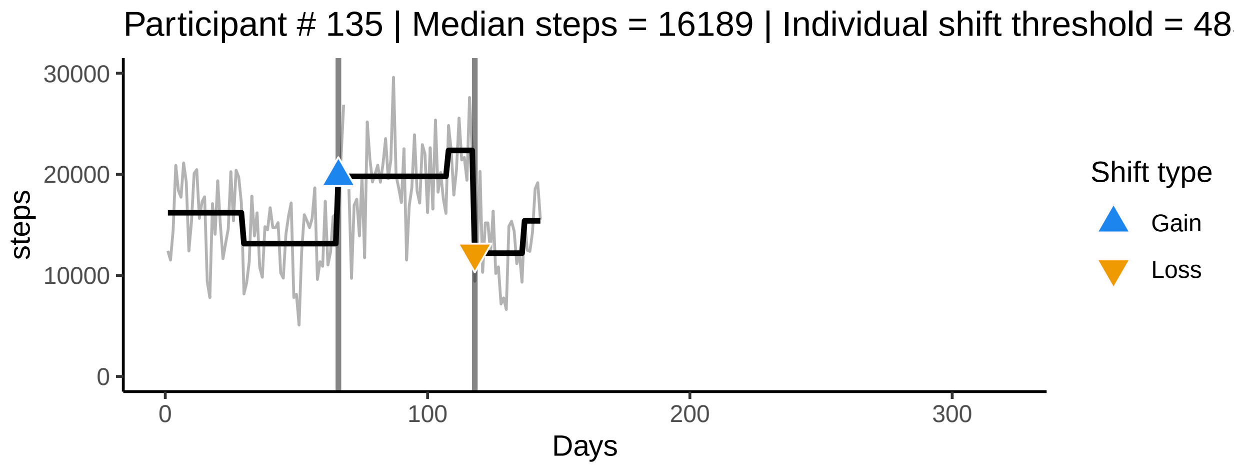
Participant # 135 | Median steps = 16189 | Individual shift threshold = 48
steps
30000
20000
10000
0
0
100
200
300
Days
Shift type
Gain
Loss

```
##
## [[136]]
```

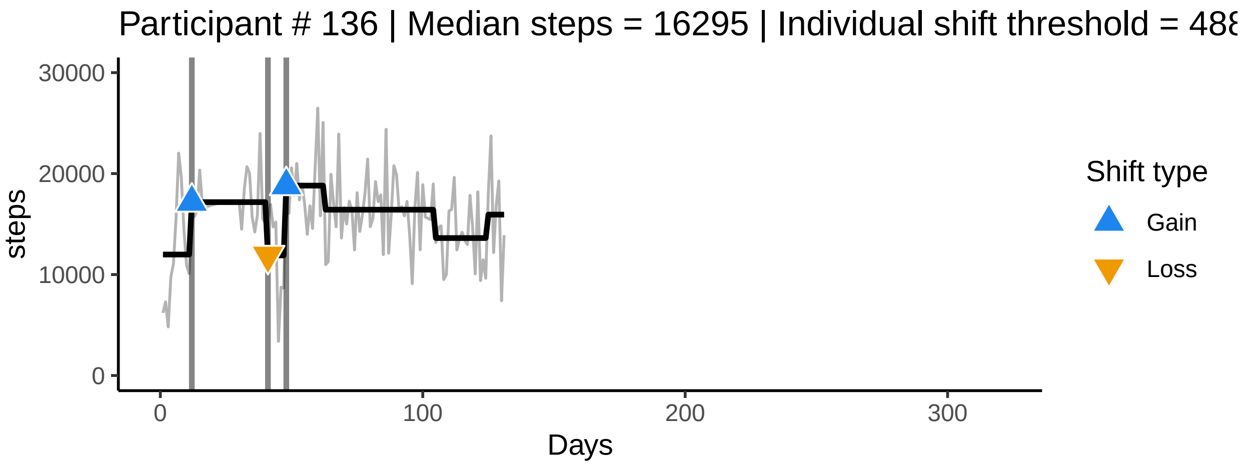
Participant # 136 | Median steps = 16295 | Individual shift threshold = 488
30000
20000
10000
0
steps
0
100
200
300
Days
Shift type
Gain
Loss

```
##
## [[137]]
```

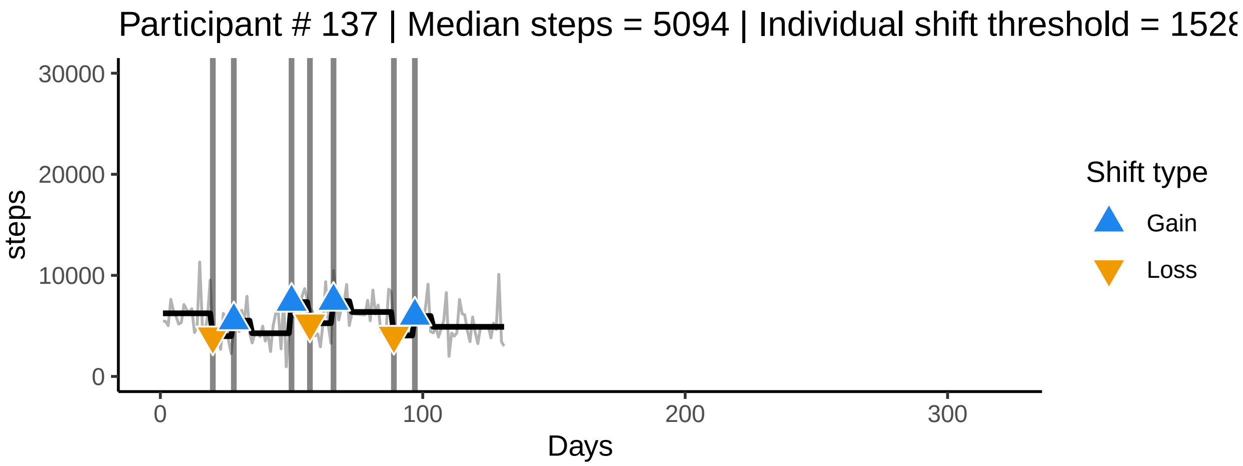
Participant # 137 | Median steps = 5094 | Individual shift threshold = 152
30000
20000
10000
0
steps
0
100
200
300
Days
Shift type
Gain
Loss

```
##
## [[138]]
```

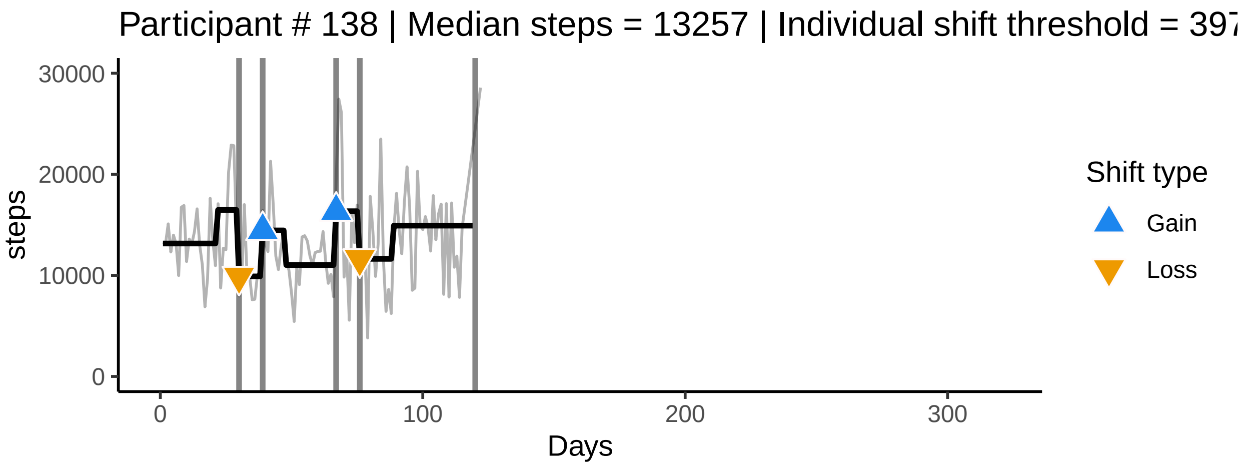
Participant # 138 | Median steps = 13257 | Individual shift threshold = 397
30000
20000
10000
0
steps
0
100
200
300
Days
Shift type
Gain
Loss

```
##
## [[139]]
```

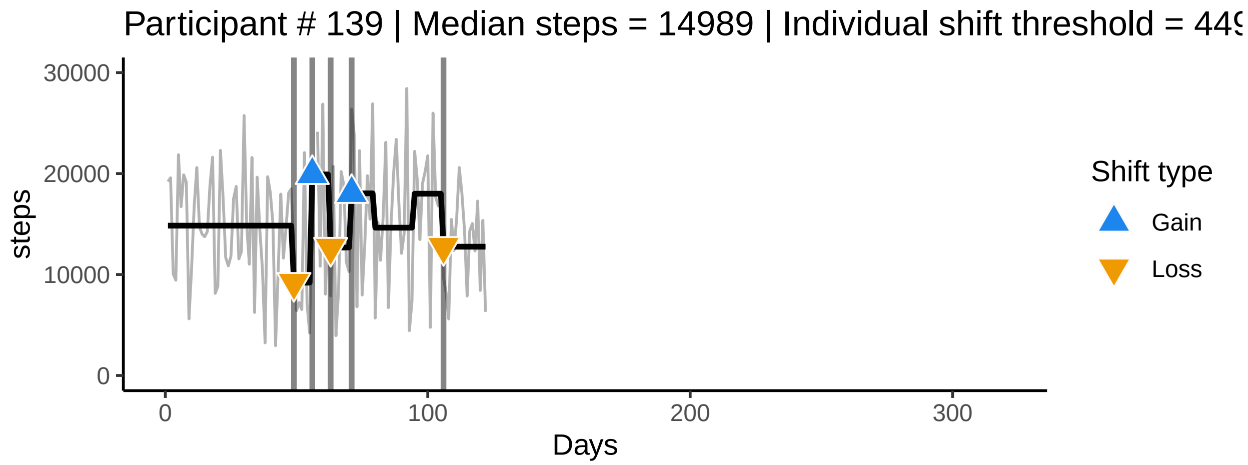
Participant # 139 | Median steps = 14989 | Individual shift threshold = 449
30000
20000
10000
0
steps
0
100
200
300
Days
Shift type
Gain
Loss

```
##
## [[140]]
```

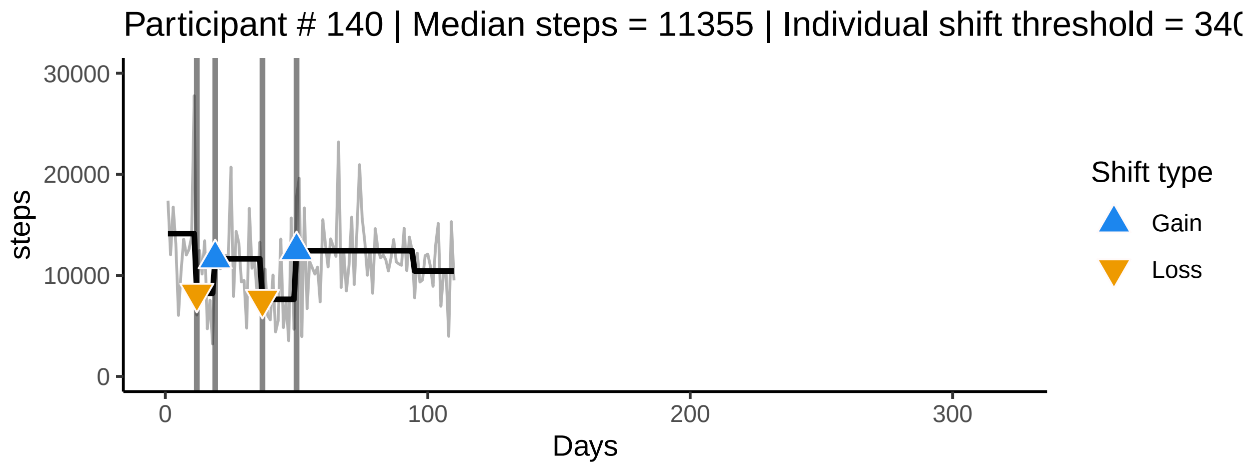
Participant # 140 | Median steps = 11355 | Individual shift threshold = 340
30000
20000
10000
0
steps
0
100
200
300
Days
Shift type
Gain
Loss

```
##
## [[141]]
```

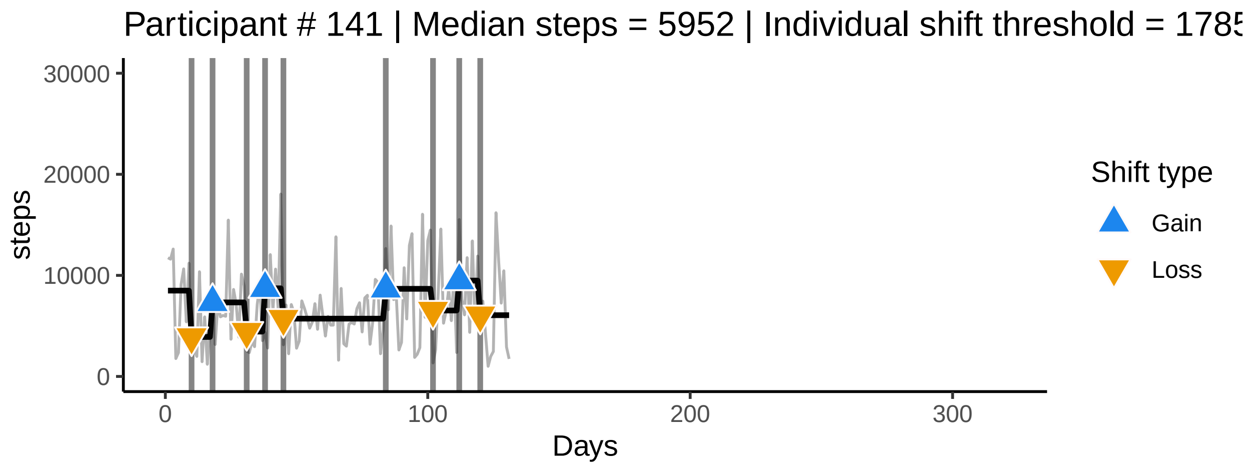
Participant # 141 | Median steps = 5952 | Individual shift threshold = 178
30000
20000
10000
0
steps
0
100
200
300
Days
Shift type
Gain
Loss

```
##
## [[142]]
```

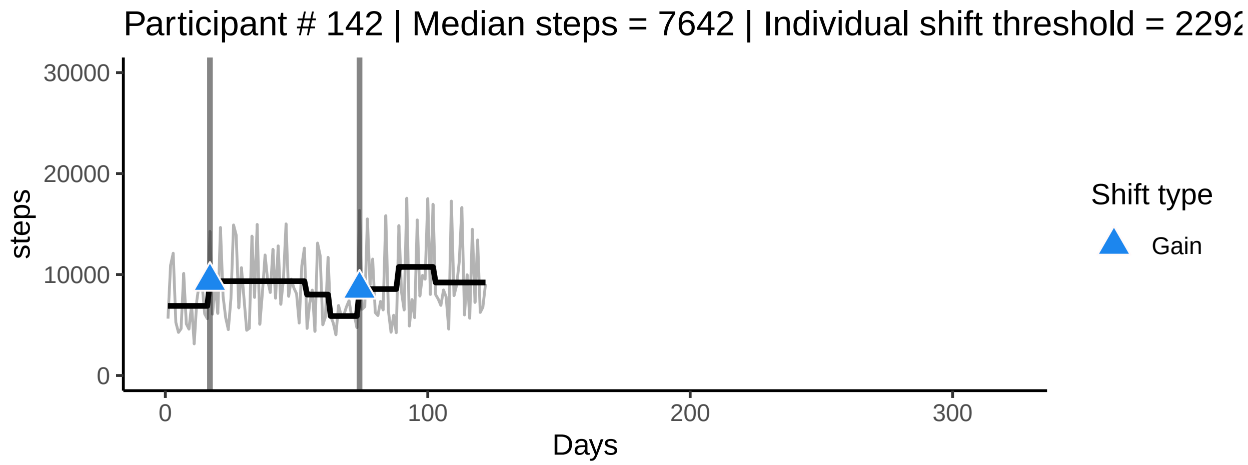
Participant # 142 | Median steps = 7642 | Individual shift threshold = 2292
steps
30000
20000
10000
0
0
100
200
300
Days
Shift type
Gain

```
##
## [[143]]
```

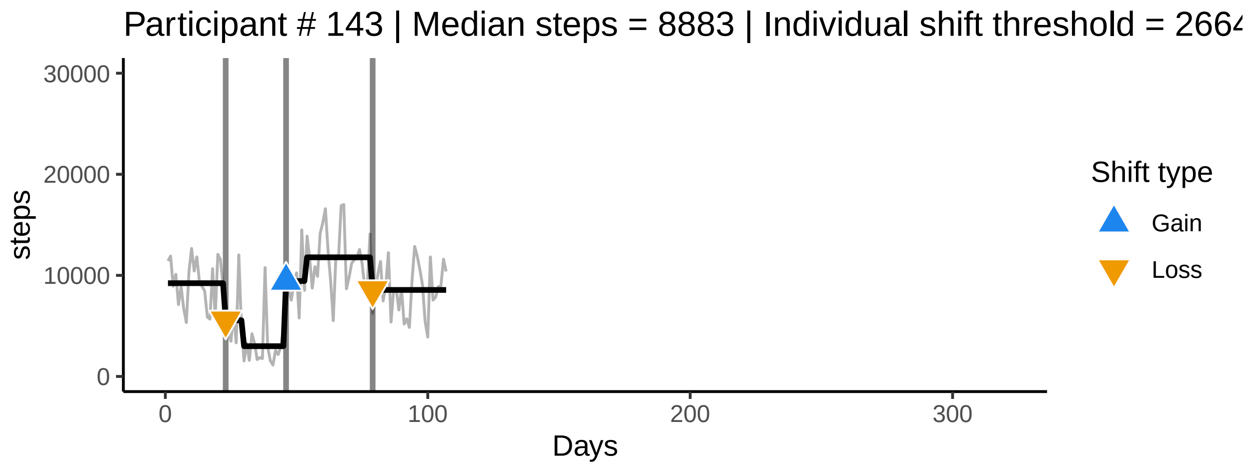
Participant # 143 | Median steps = 8883 | Individual shift threshold = 2664
steps
30000
20000
10000
0
0
100
200
300
Days
Shift type
Gain
Loss

```
##
## [[144]]
```

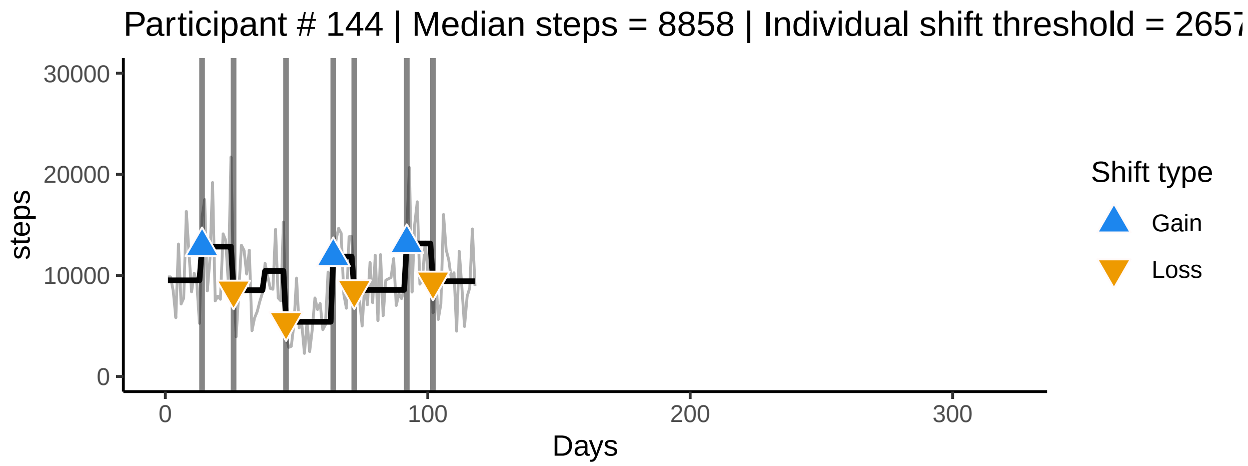
Participant # 144 | Median steps = 8858 | Individual shift threshold = 2657
steps
30000
20000
10000
0
0
100
200
300
Days
Shift type
Gain
Loss

```
##
## [[145]]
```

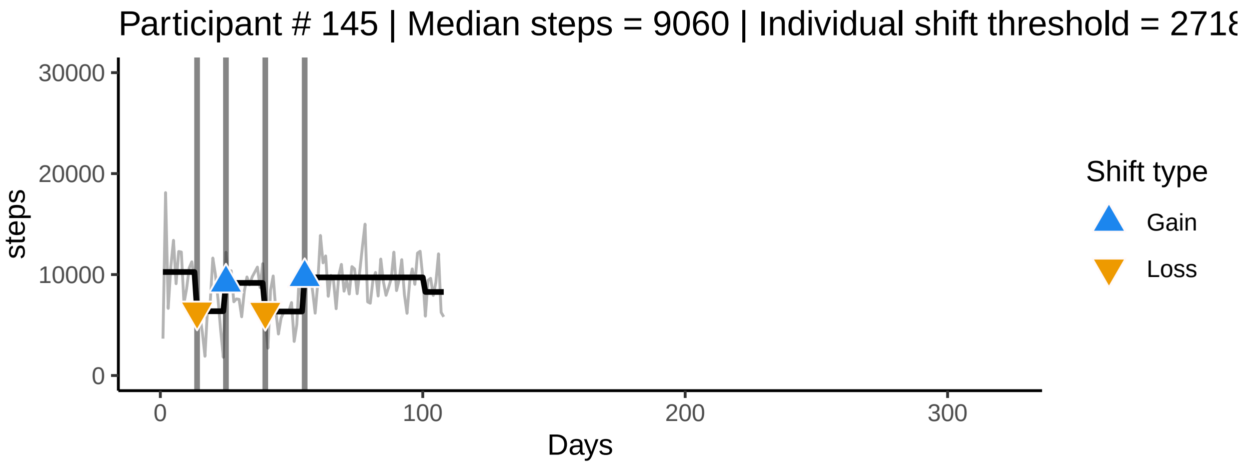

Participant # 145 | Median steps = 9060 | Individual shift threshold = 2718
30000
20000
10000
0
steps
0
100
200
300
Days
Shift type
Gain
Loss


```
##
## [[146]]
```

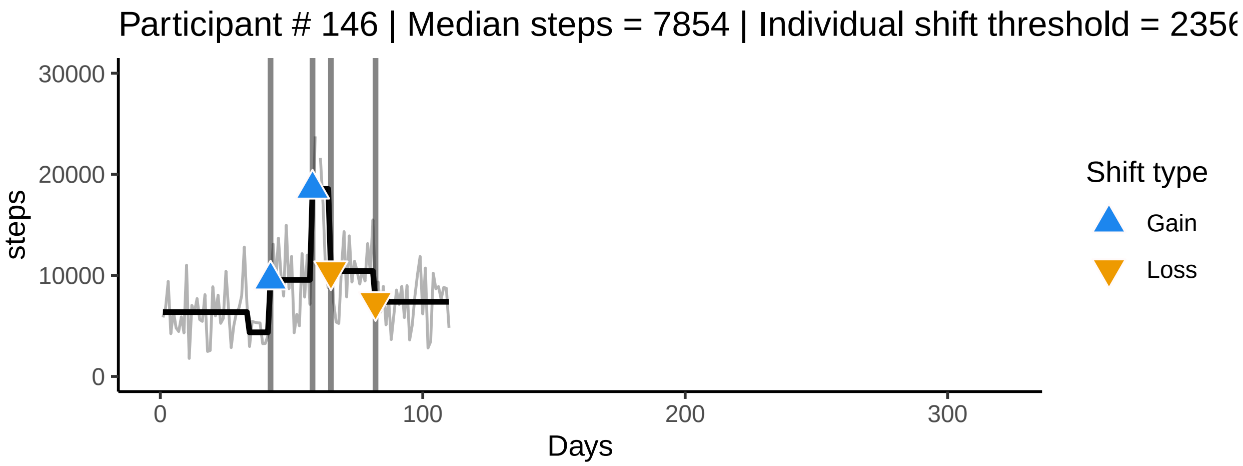

Participant # 146 | Median steps = 7854 | Individual shift threshold = 2356
30000
20000
10000
0
steps
0
100
200
300
Days
Shift type
Gain
Loss


```
##
## [[147]]
```

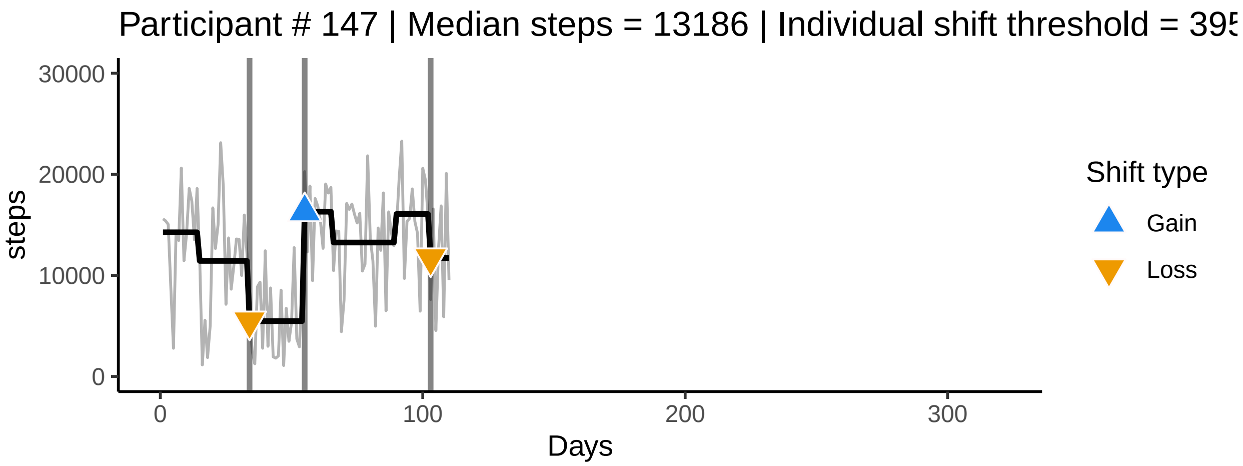

Participant # 147 | Median steps = 13186 | Individual shift threshold = 395
30000
20000
10000
0
steps
0
100
200
300
Days
Shift type
Gain
Loss

```
##
## [[148]]
```

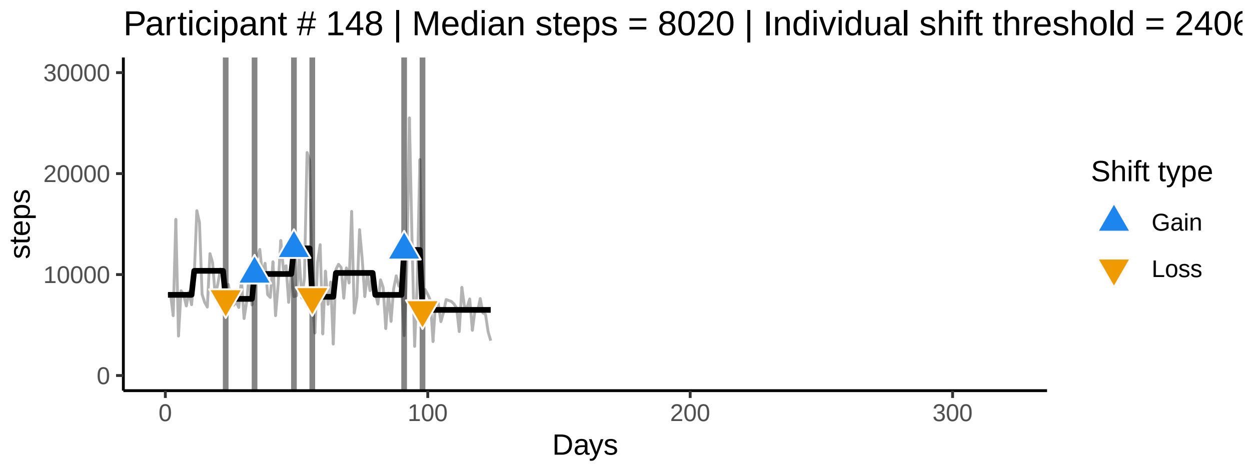
Participant # 148 | Median steps = 8020 | Individual shift threshold = 2406
30000
20000
10000
0
steps
0
100
200
300
Days
Shift type
Gain
Loss

```
##
## [[149]]
```

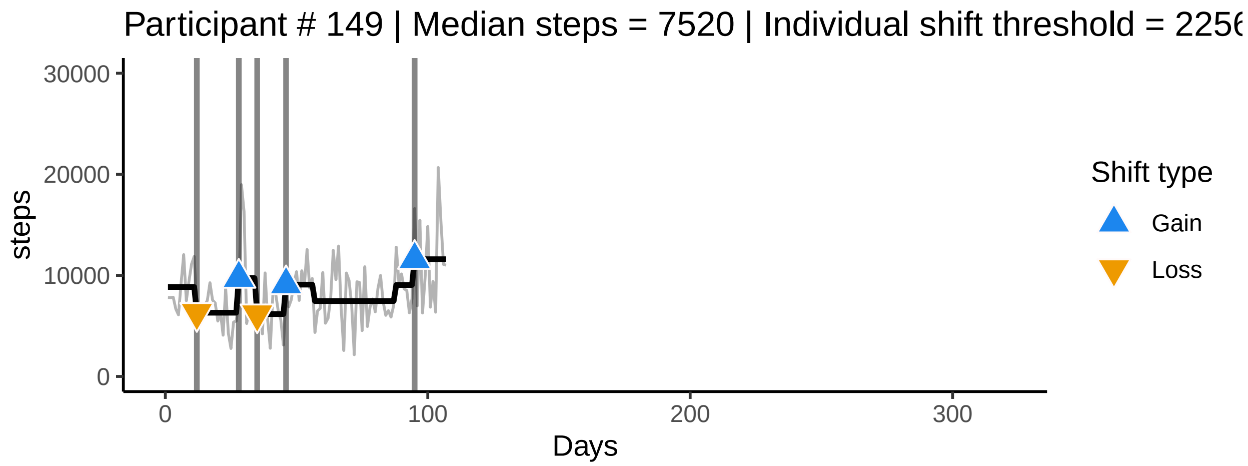
Participant # 149 | Median steps = 7520 | Individual shift threshold = 2256
30000
20000
10000
0
steps
0
100
200
300
Days
Shift type
Gain
Loss

```
##
## [[150]]
```

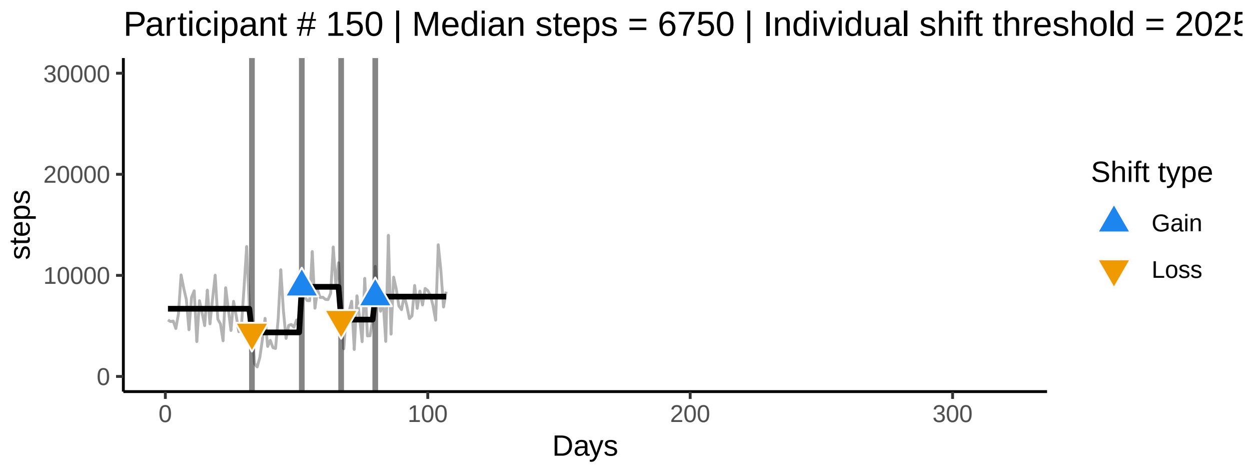
Participant # 150 | Median steps = 6750 | Individual shift threshold = 2025
30000
20000
10000
0
steps
0
100
200
300
Days
Shift type
Gain
Loss

```
##
## [[151]]
```

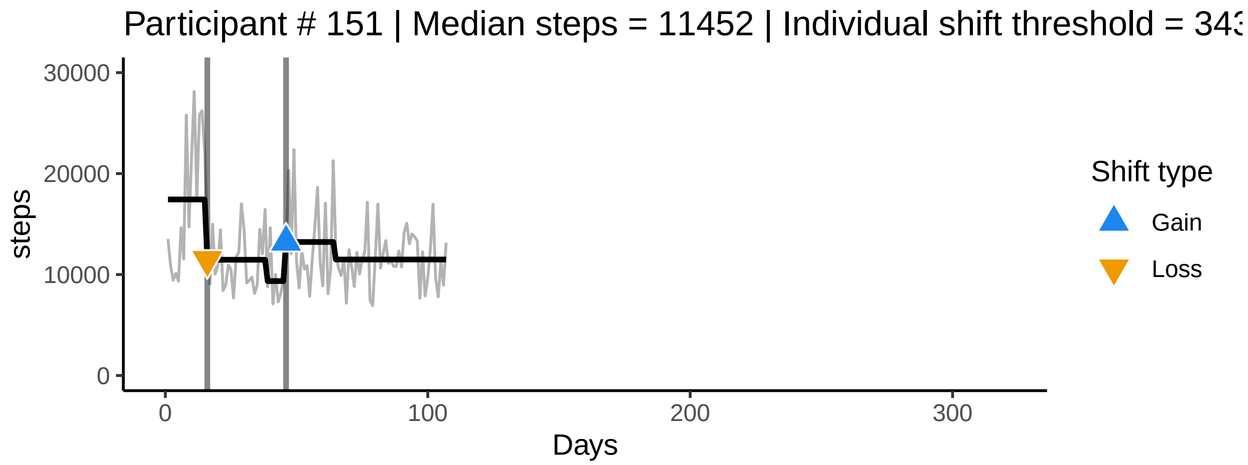
Participant # 151 | Median steps = 11452 | Individual shift threshold = 343
30000
20000
10000
0
steps
0
100
200
300
Days
Shift type
Gain
Loss
