# Supplementary material for: Characterizing and predicting person-specific, day-to-day, fluctuations in walking behavior
Source: PLoS One. 2021 May 14;16(5):e0251659. doi: 10.1371/journal.pone.0251659 (PMC8121346; doi:10.1371/journal.pone.0251659)
Supplement: S1 Table — Sensitivity analyses for varying a) thresholds for the identification of gains and losses, b) time-lapses for the computation of local dynamic complexity and c) gains and losses separately and together. (DOCX) [file pone.0251659.s002.docx]

**S1 Table.** sensitivity analyses for varying a) thresholds for the identification of gains and losses, b) time-lapses for the computation of local dynamic complexity and c) gains and losses separately and together.

| **Shift size**  **from median (%)** |  | **Gains and losses**  **(*N* = 151; *k* = 26882)** | | **Gains only**  **(*N* = 144; *k* = 25924)** | | **Losses only**  **(*N* = 140; *k* = 25031)** | |
| --- | --- | --- | --- | --- | --- | --- | --- |
|  | **Predictors** | ***OR*** | **95% CI** | ***OR*** | **95% CI** | ***OR*** | **95% CI** |
| > 20% | Time | .90 | [.83, .97] | .77 | [.70, .86] | .72 | [.64, .81] |
|  | Duration | 1.11 | [1.04, 1.19] | 1.75 | [1.60, 1.91] | 1.85 | [1.68, 2.04] |
|  | LDC_2days | 1.13 | [1.05, 1.20] | .89 | [.79, .99] | 1.38 | [1.26, 1.49] |
| > 30% | Time | .85 | [.77, .93] | .70 | [.62, .79] | .60 | [.51, .70] |
|  | Duration | 1.21 | [1.11, 1.31] | 2.04 | [1.83, 2.28] | 2.12 | [1.87, 2.42] |
|  | LDC_2days | 1.12 | [1.03, 1.21] | .86 | [.74, .98] | 1.40 | [1.24, 1.54] |
| > 40% | Time | .82 | [.73, .93] | .56 | [.47, .66] | .52 | [.41, .65] |
|  | Duration | 1.40 | [1.26, 1.54] | 2.52 | [2.19, 2.91] | 2.81 | [2.36, 3.35] |
|  | LDC_2days | 1.15 | [1.04, 1.26] | .82 | [.69, .96] | 1.48 | [1.30, 1.66] |
| > 20% | Time | .90 | [.83, .97] | .78 | [.70, .86] | .72 | [.64, .81] |
|  | Duration | 1.12 | [1.04, 1.20] | 1.76 | [1.61, 1.92] | 1.85 | [1.67, 2.04] |
|  | LDC_3days | 1.15 | [1.07, 1.22] | .93 | [.82, 1.03] | 1.40 | [1.28, 1.51] |
| **> 30%** | **Time** | **.85** | **[.77, .93]** | **.70** | **[.61, .79]** | **.60** | **[.51, .71]** |
|  | **Duration** | **1.21** | **[1.12, 1.32]** | **2.06** | **[1.84, 2.30]** | **2.11** | **[1.86, 2.40]** |
|  | **LDC_3days** | **1.14** | **[1.05, 1.24]** | **.89** | **[.77, 1.01]** | **1.43** | **[1.28, 1.58]** |
| > 40% | Time | .82 | [.73, .93] | .56 | [.47, .66] | .52 | [.41, .65] |
|  | Duration | 1.40 | [1.27, 1.55] | 2.54 | [2.21, 2.94] | 2.78 | [2.34, 3.31] |
|  | LDC_3days | 1.18 | [1.06, 1.29] | .88 | [.75, 1.02] | 1.51 | [1.33, 1.69] |
| > 20% | Time | .90 | [.83, .97] | .78 | [.70, .86] | .72 | [.64, .81] |
|  | Duration | 1.12 | [1.04, 1.20] | 1.75 | [1.61, 1.92] | 1.84 | [1.67, 2.04] |
|  | LDC_4days | 1.15 | [1.08, 1.23] | .95 | [.85, 1.04] | 1.40 | [1.28, 1.50] |
| > 30% | Time | .85 | [.77, .93] | .70 | [.61, .79] | .60 | [.51, .71] |
|  | Duration | 1.21 | [1.12, 1.32] | 2.06 | [1.84, 2.30] | 2.11 | [1.86, 2.40] |
|  | LDC_4days | 1.13 | [1.04, 1.23] | .91 | [.80, 1.03] | 1.42 | [1.27, 1.57] |
| > 40% | Time | .82 | [.73, .93] | .56 | [.47, .66] | .52 | [2.32, 3.30] |
|  | Duration | 1.40 | [1.27, 1.55] | 2.55 | [2.21, 2.94] | 2.76 | [.41, .66] |
|  | LDC_4days | 1.18 | [1.07, 1.29] | .91 | [.77, 1.04] | 1.51 | [1.33, 1.69] |

*Note:* target models are in bold
